# Sulfide regulation and catabolism in health and disease

**DOI:** 10.1038/s41392-025-02231-w

**Published:** 2025-05-30

**Authors:** Yuanyuan Hou, Boyang Lv, Junbao Du, Min Ye, Hongfang Jin, Yang Yi, Yaqian Huang

**Affiliations:** 1 Department of Pediatrics, Children’s Medical Center, Peking University First Hospital, Beijing, 100034 China; 2 State Key Laboratory of Natural and Biomimetic Drugs, School of Pharmaceutical Sciences, Peking University, Beijing, 100191 China; 3 Yunnan Baiyao International Medical Research Center, Peking University, 38 Xueyuan Road, Beijing, 100191 China; 4 State Key Laboratory of Vascular Homeostasis and Remodeling, Peking University, Beijing, 100191 China

**Keywords:** Drug screening, Cardiovascular diseases

## Abstract

The metabolic pathway of sulfur-containing amino acids in organisms begins with methionine, which is metabolized to produce important sulfur-containing biomolecules such as adenosylmethionine, adenosylhomocysteine, homocysteine, cystine, and hydrogen sulfide (H_2_S). These sulfur-containing biomolecules play a wide range of physiological roles in the body, including anti-inflammation, antioxidant stress, DNA methylation, protein synthesis, etc., which are essential for maintaining cellular function and overall health. In contrast, dysregulation of the metabolic pathway of sulfur-containing amino acids leads to abnormal levels of sulfur-containing biomolecules, which produce a range of pathological consequences in multiple systems of the body, such as neurodegenerative diseases, cardiovascular diseases, and cancer. This review traces the milestones in the development of these sulfur-containing biomolecules from their initial discovery to their clinical applications and describes in detail the structure, physiochemical properties, metabolism, sulfide signaling pathway, physiopathological functions, and assays of sulfur-containing biomolecules. In addition, the paper also explores the regulatory role and mechanism of sulfur-containing biomolecules on cardiovascular diseases, liver diseases, neurological diseases, metabolic diseases and tumors. The focus is placed on donors of sulfur-containing biological macromolecule metabolites, small-molecule drug screening targeting H_2_S-producing enzymes, and the latest advancements in preclinical and clinical research related to hydrogen sulfide, including clinical trials and FDA-approved drugs. Additionally, an overview of future research directions in this field is provided. The aim is to enhance the understanding of the complex physiological and pathological roles of sulfur-containing biomolecules and to offer insights into developing effective therapeutic strategies for diseases associated with dysregulated sulfur-containing amino acid metabolism.

## Introduction

Sulfur is an essential element in biological systems and is vital to life. In healthy individuals, sulfur is absorbed in the digestive tract in the form of sulfur-containing amino acids, of which methionine is an essential amino acid. Endogenous sulfur-containing amino acid metabolism begins with the dietary intake of methionine, and through methionine cycle pathway, S-adenosylmethionine (SAM), S-adenosylhomocysteine (SAH), homocysteine (Hcy) and methionine are produced successively. Cystathionine and cysteine are produced from Hcy via the Hcy trans-mercapto pathway. Cysteine on the one hand produces taurine and sulfur dioxide through cysteine oxidative metabolism pathway, and on the other hand produces cystathionine, cystine, cysteine and hydrogen sulfide (H_2_S) through cysteine trans-mercapto pathway. H_2_S is eventually metabolized to sulfate or dimethyl sulfide and excreted from the body. Therefore, this metabolic pathway leads to the production of key intermediate metabolites such as SAM, SAH, Hcy, cystathionine, cysteine, and cystine, as well as terminal metabolites such as H_2_S, taurine, and sulfur dioxide.

**Fig. 1:**
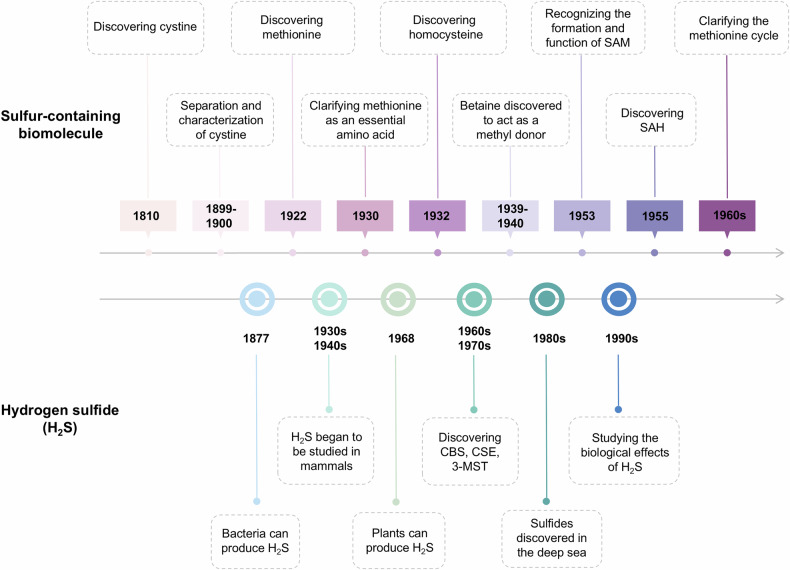
Timeline of major advancements in the study of sulfur-containing biomolecules produced by metabolism of sulfur-containing amino acids. In 1810, Wollaston identified cystine; in 1899-1900, Morner and Embden isolated cystine, showing cysteine’s interconvertibility; in 1922, Mueller isolated methionine; in the 1930s, methionine was recognized as essential for protein synthesis; in 1932, Du Vigneaud identified homocysteine; in 1953, Cantoni described SAM formation and Baddiley and Jamieson discovered SAH; and in the 1960s, methionine metabolism pathways were further elucidated. H_2_S, first observed by Ulysse Gayon in 1877 from bacteria in spoiled eggs, was linked to sulfur amino acid metabolism. In the 1930s and 1940s, Du Vigneaud discovered the transsulfuration pathway. By the 1960s and 1970s, enzymes like CBS, CSE, and 3-MST were identified. In the 1980s, sulfides were found in deep-sea vents. By the early 1990s, H_2_S production in mammals was known, with its biological effects explored later

A review of the research history reveals key milestones in the study of sulfur-containing amino acids and H_2_S (Fig. 1). In 1899-1900, Morner and Embden isolated cystine from scleroprotein, marking the identification of sulfur-containing amino acids. Later research demonstrated that cystine and cysteine are interconvertible through oxidation and reduction reactions.^1–3^ In the 1930s, methionine was recognized as an essential amino acid in protein synthesis.^4,5^ Hcy was identified in 1932 by Du Vigneaud and later recognized as an intermediate in the methionine metabolic pathway, forming cysteine through the transsulfuration pathway.^6–8^ In 1953, Giulio Cantoni described the formation and function of SAM, pivotal for methylation reactions.^9^ SAH, a product of SAM transmethylation, was synthesized by Baddiley and Jamieson in 1953.^10^ In the 1960s, scientists gradually elucidated the specific steps of methionine metabolism, revealing that methionine enters the methylation pathway via SAM, is then converted to Hcy through SAH, and subsequently re-enters the methionine cycle through remethylation reactions. H_2_S production in mammals was known biochemically by the early 1990s. Subsequent studies confirmed the presence of H_2_S-producing enzymes in the brain^11^ and blood vessels,^12^ gradually demonstrating that H_2_S plays important roles across various physiological systems. H_2_S is now recognized as the third gasotransmitter, following nitric oxide (NO) and carbon monoxide (CO).^13,14^

In a healthy state, these sulfur-containing biomolecules participate in protein synthesis, as well as the synthesis and degradation of fatty acids, and are involved in key energy metabolism processes such as the citric acid cycle. They exert neurotransmitter, modulatory, and hormone-like biological effects, protect cells from environmental toxins and drug-induced damage, and play a critical role in the oxidant-antioxidant homeostasis.^15–20^ Through these diverse functions, sulfur-containing biomolecules play a crucial role in maintaining life activities, regulating physiological processes, protecting cells from damage, and are important substances in regulating homeostasis in the body. And disruption of sulfur-containing amino acid metabolic pathways is closely linked to the development of many diseases. It is well known that long-term induced hypermethioninemia leads to hyperhomocysteinemia, which affects methylation and metabolic processes. Clinical studies have confirmed that hyperhomocysteinemia is not only an independent risk factor for the development of cardiovascular and cerebrovascular diseases, such as atherosclerosis, acute myocardial infarction, stroke, coronary artery lesions, and peripheral vascular disease, but also increases the risk of neurodegenerative disorders and tumors, and impairs the efficacy of antifolate drugs.^21^ In contrast, H_2_S has a protective effect on the development of many diseases. It prevents cardiovascular diseases such as myocardial infarction, myocardial ischemia-reperfusion (IR) injury, cardiac hypertrophy, and atherosclerosis,^22^ reduces renal IR injury,^23^ attenuates non-alcoholic fatty liver disease (NAFLD) and hepatic fibrosis,^24^ reduces brain injury, promotes neurofunctional recovery, alleviates neurodegenerative diseases,^25^ promotes dendritic development of Purkinje cells and protects the cerebellum from oxidative stress damage. H_2_S presents a complex regulatory mechanism in tumors. In tumors such as breast,^26^ colorectal,^27^ and prostate cancers,^28^ H_2_S may promote tumor growth through pro-angiogenic and anti-apoptotic effects. Nevertheless, it has also been found that H_2_S may have anti-tumor properties by inducing apoptosis and inhibiting cell proliferation.^29–31^ The role of H_2_S in diseases may vary due to individual differences, disease types, and disease stages. Therefore, further research is still needed to better understand the effect of H_2_S in the occurrence and progression of specific diseases.

This paper summarizes the structure, physicochemical properties, metabolic processes, signaling pathways, physiological and pathophysiological functions of sulfur-containing biomolecules. Furthermore, novel mechanisms such as disulfide stress, disulfidptosis, and protein sulfhydration are discussed, highlighting their diverse roles in cellular regulation and disease progression. Moreover, the detection methods were also described, with an emphasis on innovative techniques such as fluorescence probes and mass spectrometry, which allow for highly sensitive and real-time monitoring of H₂S in biological systems. In addition, this paper summarizes the currently developed donors of sulfur-containing biomacromolecule metabolites and the screening of small molecule drugs targeting hydrogen sulfide-producing enzymes. These advances open new therapeutic avenues, particularly in modulating the sulfide metabolism to address diseases like cancer, neurodegenerative disorders, and metabolic diseases. We also focus on recent advances in preclinical and clinical studies of sulfur-containing biomacromolecule metabolites, noting the critical need to overcome existing research gaps. To this end, we propose strategies such as advancing clinical trials and further exploring large animal models, which will help bridge the gap between preclinical findings and human therapeutic applications. This holistic approach provides a new research orientation in the treatment of sulfur-containing biomacromolecule metabolism imbalance-related diseases.

## Chemical biology of sulfides

### Methionine

**Fig. 2:**
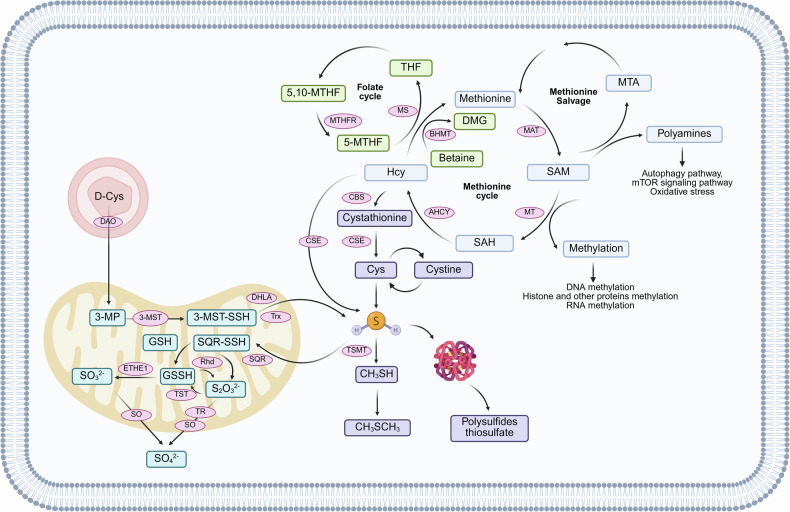
Generation and metabolism of sulfur-containing biomolecules. The metabolism of methionine begins with its breakdown by methionine adenosyltransferase (MAT), producing S-adenosylmethionine (SAM). Subsequently, SAM is converted to S-adenosylhomocysteine (SAH) through the action of methyltransferase (MT). SAH is then hydrolyzed by adenosylhomocysteinase (AHCY) to homocysteine (Hcy). Hcy can enter the transsulfuration pathway or be recycled back into methionine via the methionine synthase (MS) pathway or the betaine-homocysteine S-methyltransferase (BHMT), completing the methionine cycle. Within the MS pathway, Hcy also participates in the folate cycle. Additionally, SAM can contribute to methionine salvage pathways via MAT to replenish methionine levels. In the transsulfuration pathway, Hcy can be converted to cysteine (Cys) via cystathionine *β*-synthase (CBS) and cystathionine *γ*-lyase (CSE), leading to hydrogen sulfide (H_2_S) production. Hcy can also be directly converted to H_2_S by CSE. Furthermore, Cys and cystine can interchange with each other. D-amino acid oxidase (DAO), located in peroxisomes, can utilize D-cysteine (D-Cys) as a substrate to produce 3-mercaptopyruvate (3-MP), which can then be converted to H_2_S by 3-mercaptopyruvate sulfurtransferase (3-MST). The metabolism of H_2_S in the body can occur through simple gas exhalation, as well as via oxidation (which occurs in the mitochondria), methylation, and clearance by methemoglobin. Created with BioRender.com

Amino acids are the fundamental elements of proteins and are involved in various critical biological processes in the body, such as metabolism,^32–34^ growth and development,^35–37^ and immune function.^36,38–40^ In prokaryotes, nearly all proteins are initiated with N-formylmethionine, whereas in eukaryotes, protein synthesis starts with methionine.^41^ For humans, methionine is the only sulfur-containing essential amino acid. Methionine exists in two isomeric forms, L-methionine and D-methionine, with the former being the predominant form found in nature.^42^ Methionine has a slightly distinctive odor and is unstable in strong acids, which can lead to demethylation. Additionally, it is soluble in water, dilute acids, and dilute bases, while being sparingly soluble in ethanol.^42^ The metabolism of methionine begins with its breakdown by methionine adenosyltransferase (MAT), producing the universal methyl donor SAM. SAM then donates its methyl group via the action of methyltransferase (MT), resulting in the formation of SAH. SAH is hydrolyzed by adenosylhomocysteinase (AHCY) into Hcy and adenosine. Hcy can either enter the transsulfuration pathway or be remethylated back to methionine via the methionine synthase (MS) pathway or the betaine-homocysteine S-methyltransferase (BHMT) pathway, thereby completing the methionine cycle^41,43,44^ (Fig. 2). In addition to serving as a precursor for protein synthesis, methionine’s broader physiological functions are primarily mediated through the intermediate products of the methionine cycle, such as the methylation processes dependent on its derivative SAM. Furthermore, elevated levels of Hcy, a byproduct of methionine metabolism, are associated with cardiovascular diseases and neurodegenerative disorders.^45^ Methionine can also be converted into cysteine, which is involved in the synthesis of glutathione. Glutathione is a critical intracellular antioxidant that scavenges free radicals and protects cells from oxidative stress-induced damage. Therefore, methionine plays a key role in maintaining cellular redox balance.^16,46–48^ The current limitations in methionine research primarily lie in the incomplete understanding of its complex role in various diseases. While methionine is associated with several metabolic disorders and cancers, the specific pathological effects of its excess or deficiency remain unclear. Existing therapeutic strategies mainly focus on regulating homocysteine levels, with limited effective interventions targeting methionine itself.

### S-adenosylmethionine

As early as 1951, Giulio Cantoni^49^ discovered SAM which is formed when methionine binds to the adenosyl group of ATP. SAM is a typical sulfonium compound, where the sulfur atom is covalently bonded to three substituents arranged in a trigonal pyramidal geometry, with a lone pair of electrons also associated with the sulfur atom.^50^ The high-energy sulfonium ion present in SAM enables it to transfer its methyl group to a variety of substrates via substitution reactions, including proteins, DNA, RNA, and metals.^51^ The most prominent metabolic function of SAM is its role as a methyl donor, with over 90% of the SAM produced being consumed in methylation reactions.^52–54^ DNA methyltransferases (DNMTs) transfer the methyl group from SAM to DNA, typically adding the methyl group to the 5^th^ carbon of cytosine, forming 5-methylcytosine. This DNA methylation plays a crucial role in the regulation of gene expression and is often associated with gene silencing.^55^ SAM is also involved in RNA methylation modifications, such as N6-methyladenosine (m6A) modification in mRNA. This modification influences mRNA stability and translation efficiency, thereby regulating gene expression.^56^ SAM is also a critical methyl donor for histone methylation. Histone methylation is carried out by histone methyltransferases (HMTs) and typically occurs on lysine or arginine residues. This modification affects chromatin structure and regulates gene expression.^57^ SAM also participates in the synthesis of polyamines. In this pathway, SAM is decarboxylated by SAM decarboxylase, generating decarboxylated SAM (dcSAM). Subsequently, spermidine synthase catalyzes the addition of the first aminopropyl group from dcSAM to putrescine, forming spermidine (SPD). SPD is then converted into spermine (SPM) by spermine synthase through the addition of a second aminopropyl group. Both reactions generate 5′-methylthioadenosine as a byproduct.^58–60^ SPD and SPM are involved in various cellular processes, such as the regulation of autophagy pathways, mTOR signaling, and oxidative stress and antioxidant pathways.^61–63^ Methylthioadenosine (MTA) inhibits both spermidine synthase and spermine synthase. As a result, MTA can be recycled into methionine through the methionine salvage pathway.^64^ MTA is phosphorylated by MTA phosphorylase (MTAP), and subsequently converted into adenine and 5-methylthioribose-1-phosphate, the latter of which is further metabolized into methionine. In the absence of MTAP, endogenous MTA cannot be salvaged into methionine, leading to the accumulation of dcSAM and MTA, both of which inhibit methylation reactions.^65–67^ In addition, SAM mediates radical-based chemical reactions. Several SAM-dependent radical enzymes exist in the body, and the SAM radical enzyme family can form characteristic [4Fe-4S] clusters. These clusters provide the electrons necessary for the reductive cleavage of SAM, generating a 5’-deoxyadenosyl radical, which initiates radical mechanisms.^68,69^ Despite its critical involvement in methylation reactions, the precise mechanisms by which SAM dysregulation contributes to diseases such as cancer, neurodegenerative disorders, and liver diseases are still not fully elucidated. Moreover, while SAM supplementation has shown promise in some clinical settings, its therapeutic application is limited by the lack of standardized dosing and concerns over potential side effects.

### S-adenosylhomocysteine

AHCY is the only enzyme that catalyzes the reversible hydrolysis of SAH into adenosine and homocysteine, thereby maintaining methylation homeostasis within the methionine cycle.^70^ Excessive accumulation of SAH within the cell can inhibit the activity of SAM-dependent methyltransferases.^71^ Therefore, the ratio of SAM/SAH is often used as an indicator of cellular methylation capacity, with a decrease in this ratio signaling a reduction in cellular methylation potential.^72^ Previous Study has shown that SAH can reduce the expression of DNA methyltransferase DNMT1 protein, lead to demethylation of the CpG islands in the NF-κB gene promoter region, and increase NF-κB expression, trigger the expression of pro-inflammatory senescence-associated secretory phenotype (SASP) factors, and thus induce cellular senescence in rat aortic smooth muscle cells.^73^ Additionally, inhibition of AHCY can induce endothelial cell senescence by downregulating hTERT expression, which is linked to reduced histone methylation in the hTERT promoter region.^74^ Notably, AHCY inhibition can also promote the development of atherosclerosis; it leads to downregulation of DNMT1 expression, which subsequently triggers the Drp1-mitochondrial reactive oxygen species (mtROS) pathway, ultimately resulting in atherosclerosis.^75^ Conversely, overexpression of AHCY can rescue cell function by activating the Nrf2-HO-1 signaling pathway. Transplantation of diabetic bone marrow mesenchymal stem cells with AHCY overexpression can enhance angiogenesis and mitigate adverse cardiac remodeling in rats.^76^

### Homocysteine

Hcy is a sulfur-containing, non-proteinogenic amino acid derived from methionine and is a homolog of cysteine,^77^ with an additional methylene group (-CH2-) preceding the thiol group (-SH) on its side chain. Homocysteine is converted back to methionine via the MS pathway and the BHMT pathway, thus fulfilling a biological function of protecting methionine. In the MS pathway, 5,10-methylene tetrahydrofolate (5,10-MTHF) is converted into 5-methyl tetrahydrofolate (5-MTHF) by methylenetetrahydrofolate reductase (MTHFR). 5-MTHF then donates a methyl group to Hcy under the catalysis of MS, forming methionine. Subsequently, 5-MTHF is converted into tetrahydrofolate (THF). THF can be reconverted into 5,10-MTHF to complete the folate cycle, a process that requires normal levels of folate and vitamin B12.^78,79^ This also demonstrates the role of Hcy as an essential substrate in the folate cycle. In the BHMT pathway, Hcy uses betaine as a methyl donor, ultimately producing methionine and dimethylglycine (DMG).^80^ Betaine is a metabolite of choline, and the process is also a necessary step in the catabolism of choline.^81^ Another important physiological function of Hcy is its entry into the transsulfuration pathway. As early as the 1930s and 1940s, Du Vigneaud began studying the oxidation of sulfur-containing amino acids in tissues and whole animals, ultimately discovering the transsulfuration pathway.^7,8^ Through this pathway, Hcy can produce cysteine as well as the important gaseous signaling molecule H_2_S. Notably, SAM indirectly participates in the transsulfuration pathway by inhibiting MTHFR^82^ and activating CBS.^83^ When SAM is depleted, Hcy is remethylated back to methionine to regenerate SAM. Conversely, when SAM levels are high, Hcy is directed toward the transsulfuration pathway. Elevated levels of Hcy are considered an independent risk factor for the development of cardiovascular diseases. Hcy can induce endothelial dysfunction and is associated with atherosclerotic vascular diseases and ischemic heart attacks.^84–86^ Additionally, high Hcy levels are well-established risk factors for neurological disorders such as dementia and cognitive impairment.^87–89^ While elevated Hcy levels are associated with cardiovascular, neurodegenerative, and metabolic disorders, the direct causality between Hcy and these diseases remains unclear. Furthermore, therapeutic approaches targeting Hcy metabolism, such as supplementation with B vitamins, show inconsistent results, highlighting the need for more effective and standardized treatments.^90,91^

### Cystine

Cystine is a dimer formed by the linkage of two cysteine molecules through a disulfide bond. Cystine has better solubility in acidic and alkaline solutions but is relatively unstable, easily reduced back to two cysteine molecules in a reductive environment. The primary physiological function of cystine is its reduction to cysteine, which then participates in the synthesis of glutathione. Under the catalysis of γ-glutamylcysteine synthetase (GCL) and in the presence of ATP, glutamate and cysteine form γ-glutamylcysteine. This step is the rate-limiting step in glutathione synthesis and serves as a key regulatory point in the process.^92^ Subsequently, γ-glutamylcysteine combines with glycine under the catalysis of glutathione synthetase, forming glutathione. This reaction also requires ATP for energy.^93^ The function of the cystine/glutamate antiporter (system x(c)(-)) is crucial for maintaining glutathione levels,^94^ and plays a significant role in ferroptosis.^95–97^

### Hydrogen sulfide

H_2_S is a colorless, rotten egg flavor, soluble in water gas.^98^ H_2_S is a weak acid with an equilibrium state: in a 140 mM NaCl solution at pH 7.4, 14% of the free sulfide is H_2_S gas, 86% is HS,^-^ and a trace amount of S.^2–122^ CBS and CSE mediate the transsulfuration pathway to produce H_2_S (Fig. 2). CBS first catalyzes the β-substitution reaction of L-homocysteine (L-Hcy) with L-serine to produce cystathionine, followed by α, γ-elimination reactions of CSE, which catalyzes the production of cystathiones to produce L-cysteine (L-Cys), α-ketobutyric acid, and NH_3_.^123^ L-Cys and water can further react to generate H_2_S. It should be noted that in this reaction, CBS generates H_2_S and serine through β-elimination reaction, while CSE generates H_2_S and pyruvate through α, β-elimination reaction.^124^ Additionally, both CBS and CSE can catalyze β-substitution reactions, resulting in the condensation of two L-Cys molecules to produce H_2_S and lanthionine, or the condensation of L-Hcy and L-Cys through β, γ-substitution reactions to produce H_2_S and cystathionine. Among them, CBS catalyzed the condensation of L-Hcy and L-Cys more predominantly, and L-Hcy and L-Cys were the most suitable substrates for the enzymatic reaction of CB.^29^ CSE can also catalyze the reaction of L-Hcy with water via α, γ-elimination reactions to produce homoserine, α-ketobutyrate, and NH_3_, or it can condense two molecules of L-Hcy through γ-substitution reaction to ultimately generate H_2_S.^125^ CBS is considered the main H_2_S synthesizing enzyme in the central nervous system, but it is also expressed in other organs such as the kidneys, liver, and lymphocyte.^126^ Compared to CBS, CSE has a wider distribution in mammalian tissues, mainly expressed in the periphery, responsible for H_2_S production in peripheral tissues,^127^ and is highly expressed in the cardiovascular and respiratory system.^128^ The third key enzyme for H_2_S generation is 3-MST, which catalyzes the synthesis of endogenous H_2_S using 3-mercaptopyruvate (3-MP) as a substrate^129^ (Fig. 2). 3-MP is produced from L-Cys by cysteine aminotransferase (CAT) with α-ketoglutarate as a coenzyme. 3-MST can remove sulfur from 3-MP, resulting in the creation of a persulfide on the enzyme (3-MST-ssh). H_2_S can be liberated from 3-MST-SSH through endogenous reducing agents like thioredoxin (Trx) or dihydrolipoic acid (DHLA).^130,131^ 3-MST exhibits high activity in various tissues, such as kidney proximal renal tubular epithelial cells, cardiac cells and liver cells. Currently, 3-MST is believed to be the main enzyme in mitochondria that catalyzes H_2_S production.^131,132^

Studies^129^ have shown that D-amino acid oxidase (DAO) also contributes to endogenous H_2_S production (Fig. 2). DAO utilizes D-cysteine (D-Cys) as a substrate to produce 3-MP, which is then converted to H_2_S by the action of 3-MS.^133^ It is worth noting that, unlike the best H_2_S production under alkaline conditions using L-Cys as a substrate, D-Cys has the best H_2_S production under neutral conditions, specifically at pH 7.4. Furthermore, the endogenous H_2_S generation pathway using D-Cys as a substrate primarily functions in the cerebellum and kidneys, which are 7-and 80-fold higher than the source of L-Cys as a substrate generation pathway, respectively. Meanwhile, DAO is localized in peroxisomes, while 3-MST mainly exists in mitochondria. The two exchange various metabolites through specific forms of vesicular transport,^134^ that is, 3-MST and DAO produce H_2_S through organelle interactions.

Metabolism of H_2_S can occur through simple gaseous exhalation,^135^ or through oxidation, methylation, and scavenging by methemoglobin (Fig. 2). Research have shown that after intravenous injection of sodium sulfide in rats, a significant amount of exhaled H_2_S gas can be detected. This finding was subsequently confirmed in humans, where an increase in exhaled H_2_S gas was observed during intravenous injection of sodium sulfide.^136,137^ The final products of protein hydrolysis by oral microbial organisms often include H_2_S gas, which is also considered a potential underlying cause of halitosis.^138^ Increased levels of H_2_S in exhaled gas have also been observed in newborns and children with sepsis. Therefore, endogenous H_2_S can be eliminated in the form of gas. Considering that the production and metabolism of endogenous H_2_S can alter under various pathological and physiological conditions, it may be worth exploring exhaled H_2_S gas as one of the diagnostic indicators. However, it is important to note that exhaled H_2_S is a minor (<1%) route of elimination in the human bod,^137^ and attention should still be paid to other metabolic pathways.

The metabolism of H_2_S primarily occurs through mitochondrial oxidation. Sulfide:quinone oxidoreductase (SQR) is located in the inner membrane of the mitochondria and initiates the irreversible oxidation of H_2_S. Through the oxidation of H_2_S, SQR introduces electrons into the electron transport chain by transferring electrons from H_2_S to the oxidized form of coenzyme Q (CoQ), eventually resulting in ATP production.^139^ At the same time, H_2_S is oxidized to sulfur atoms and bound to the SQR to produce SQR persulfide (SQR-SSH). Subsequently, there are two pathways for further metabolism of SQR-SSH. The first pathway involves the transfer of sulfur atoms from SQR-SSH to sulfite (SO_3_^2-^), forming thiosulfate (S_2_O_3_^2-^), the sulfur atoms are transferred by thiosulfate sulfotransferase (TST) to reduced glutathione (GSH) to form Glutathione persulfide(GSSH) while regenerating SO_3_.^2-^ In another pathway, sulfur atoms in SQR-SSH are transferred directly to GSH to form GSSH, which is then oxidized to SO_3_^2−^ by persulfide dioxygenase (ETHE1). SO_3_^2-^ can be further oxidized to sulfate (SO_4_^2-^) by sulfite oxidase (SO) and excreted by the kidneys.^140,141^ Notably, rhodanese (Rhd) can transfer sulfur from GSSH to SO_3_^2-^, which in turn generates S_2_O_3_^2-^, most of which is further metabolized to sulfate by thiosulfate reductase (TR) and SO.^142^

Methylation is another pathway for the metabolism of H_2_S. Unlike oxidation, methylation of H_2_S occurs in the cytoplasm and is catalyzed by Thiol S-methyltransferase (TSMT).^143^ Methylation of H_2_S produces methanethiol (CH_3_SH), which can be further methylated to produce a relatively non-toxic compound, dimethyl sulfide (CH_3_SCH_3_). Both methylation products are sufficiently volatile to be excreted by respiration. On this basis, the conversion of CH_3_SH to CH_3_SCH_3_ is slower than the initial conversion of H_2_S to CH_3_SH, so the methylation of H_2_S is much slower than oxidation.^143^ TSMT is a widely distributed enzyme, with the highest activity in the mucosa of the colon and cecum. Additionally, it has also been reported to be active in the live^144^ and brain.^145^

H_2_S can also be eliminated by methemoglobin. H_2_S can rapidly bind to the Fe^3+^ in methemoglobin (MetHb), eventually producing heme-bound polysulfides and free thiosulfate, with Fe^3+^ reduced to Fe.^2+^ This process does not interfere with the function of hemoglobin as an oxygen carrier in the blood.^146^ Furthermore, based on the inherent binding characteristics of MetHb and H_2_S, Yuto Suzuki^147^ have designed and developed MetHb-albumin clusters as an antidote for H_2_S poisoning. Clusters of MetHb-albumin contain a ferric Hb core coated with three human serum albumins covalently. Rat cardiomyocytes (H9c2) death exposed to H_2_S can be inhibited by MetHb-albumin clusters while maintaining mitochondrial function. Additionally, they can restore cytochrome c oxidase activity in mice with lethal H_2_S toxicity.

SAM interacts with nucleic acids and plays a crucial role in cellular methylation processes. SAM serves as an essential methyl donor for DNMT, facilitating DNA methylation reactions.^148^ Additionally, SAM is involved in the methylation of RNA, particularly in RNA modifications, where it plays a critical role in regulating RNA stability, processing, and function.^149^ The thiol group (-SH) of cysteine can bind to metal ions such as copper, iron, and zinc, forming chelates that influence the biological functions of these metal ions. For example, cysteine’s interaction with copper ions can inhibit copper-induced oxidative reactions, thereby protecting cells from oxidative damage caused by metals.^150,151^ Chemical interaction of H_2_S with nitric oxide (NO) could generate several intermediates, including nitrosothiol, thionitrous acid, nitroxyl, nitrosopersulifide, polysulfides, SULFI/NO, etc.^152^ These intermediates play a number of biological roles. For example, nitroxyl and SULFI/NO exert positive inotropic effects. Nitroxyl reduces blood pressure in spontaneously hypertensive rats^153^ and attenuates myocardial ischemia-reperfusion injury.^154^ Polysulfides modulate the release of neurotransmitters.^155^ In addition, H_2_S may interact with other reactive species (e.g., oxygen, nitrogen, sulfur and selenium), leading to the formation of numerous products, contributing mostly to the redox biology of the cell. H₂S also interacts with proteins. For instance, H₂S can undergo sulfhydration reactions with cysteine residues within proteins, leading to the formation of sulfhydryl modifications.^156^ Moreover, H₂S may interact with the metal centers of target proteins,^157^ forming metal-sulfide complexes that regulate the activity of metal ions. This interaction is particularly significant in redox reactions. The interactions of sulfur-containing biomolecules with other biomolecules and the subsequent generation of intermediates and products which might be new signal molecules are becoming a new research field. More and more studies are conducted to clarify the exact production mechanisms and biological importance of these hybrid molecules.

## Detection of sulfide metabolites

The methylene blue colorimetric method is the simplest technique for detecting sulfide metabolite H_2_S release. It has been applied in studies to measure H_2_S production in the human internal mammary artery^158^ and human uterine artery,^159^ as well as to assess H_2_S levels in the serum of children with Kawasaki disease.^160^ The principle of this method is based on the reaction of H_2_S with N,N-dimethyl-p-phenylenediamine solution, resulting in the formation of methylene blue. The methylene blue colorimetric assay is simple, uses relatively inexpensive reagents, has a short detection time, and is suitable for high-throughput screening. However, this method can only accurately detect H_2_S concentrations above 1 μM and is unable to measure H_2_S levels in the nmol/L range, with significant variability in the results.^161^ The sulfur-sensitive electrode method is characterized by a wide measurement range, as well as good stability and reproducibility.^162,163^ In recent years, it has been applied to measure H_2_S levels in various tissues, including the heart,^164^ brain,^165^ liver,^166^ and stomach^167^ of rats. However, during detection, Ag_2_S can form on the electrode surface, leading to decreased sensitivity and altered performance. In recent years, numerous sensitive, real-time monitorable, and structurally novel fluorescent probes for H_2_S detection have been reported, including probes selectively reduced by H_2_S,^168–170^ probes reacting with H_2_S via nucleophilic reactions,^171–175^ and probes using ligand metals to trap H_2_S.^176,177^ These fluorescent probes have great potential as tools for the detection of H_2_S in biological samples. Gas chromatography (GC) and high-performance liquid chromatography (HPLC) have been extensively reported for the determination of endogenous H_2_S. GC can be combined with chemiluminescence detection for the analysis of H_2_S levels in biological samples. And it can also be coupled with mass spectrometry (GC-MS) to detect sulfides and thiosulfates in sample.^178,179^ This method demonstrates high detection sensitivity; however, the derivatization process may influence the concentration of original sulfides in the samples. Additionally, the settings of the instrumental parameters can impact the results, necessitating a high level of proficiency from the operators. The basic operation of HPLC involves the rapid derivatization of sulfides in the sample with an excess of monobromobimane (MBB) under mild conditions, yielding sulfide-dibimane. Dibimane is a hydrophobic molecule, and sulfide-dibimane is more hydrophobic than most physiological thiols. Based on this property, sulfide-dibimane was separated by reverse phase-HPLC with gradient elution and analyzed by fluorescence detector.^180^ This method exhibits high sensitivity, low detection limits (0.02 pM), good selectivity, and the ability to rapidly capture active sulfides without releasing chemically bound sulfur that may be present in biological matrices. However, the method still has non-negligible drawbacks, such as expensive reagents, stringent reaction conditions, and long analysis times. Despite the various disadvantages of chromatography, this method is commonly employed in the detection of clinical plasma samples^181,182^ (Table 1).

**Table 1:** Comparisons of commonly used H_2_S detection approaches

Approaches		LOD	Biological test sample	Advantage	Disadvantage
Methylen blue colorimetry^158–161^		1 μM	Human internal mammary artery, Human uterine artery	Simple testing process, Cheap reagents, Short testing time,	Low sensitivity, Vulnerable to be interfered
Sulfur-sensitive electrode^162–167^		≥100 nM	Rat heart, brain, liver, stomach	High sensitivity, Real time detection	Alkaline environment interference, Frequent instrument calibration
Fluorescence probe	Reduction-based probes^168–170^	5.2 nM - 0.1 µM	Huh7 cells, HeLa cells, nude mice	High selectivity, Fast response	Vulnerable to be interfered, Photochemical labile
	Nucleophilic-based probes^171–175^	47 nM - 86 nM	COS-7, HeLa cells, HepG2 cells	High selectivity, Fast response, Commercialization	Vulnerable to be interfered
	Metal coordination-based probes^176,177^	47 nM - 0.5 μM	NIH/3T3 fibroblast cells, Human serum	High sensitivity, Well light stability	Slow detection, Poor reversibility
Chromatography	GC^178,179^	0.5 pM	biological samples such as plasma, tissue homogenate	High sensitivity, Precise separation of H_2_S	Precise parameter setting, Time-consuming process
	HPLC^180–182^	0.02 pM	biological samples such as plasma, tissue and cell culture lysates, or media	High sensitivity, Precise separation of H_2_S	Expensive reagent, Harsh reaction conditions, Long analysis time

*GC* gas chromatography, *HPLC* high performance liquid chromatography, *LOD* limit of detection

## Sulfide catabolism and diseases

### Cardiovascular disease

**Fig. 3:**
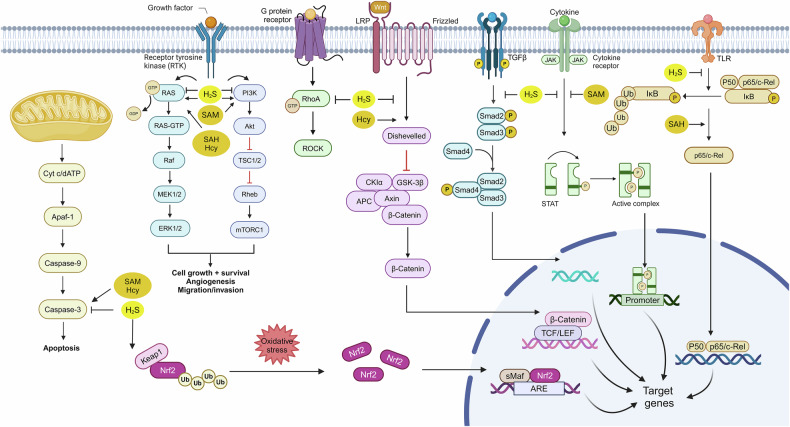
Sulfide signaling pathway. SAM and Hcy promote the activation of caspases, leading to apoptosis, while H_2_S exerts an inhibitory effect. In the ERK1/2 pathway, SAH, Hcy, and H_2_S demonstrate a promoting role. Both SAM and H_2_S activate the PI3K/Akt pathway. H_2_S inhibits RhoA, β-catenin, and TGF-β/Smad signaling, whereas Hcy promotes β-catenin nuclear translocation but downregulate its protein expression. Additionally, SAM and H_2_S can suppress the activity of the STAT pathway. SAH enhances NF-κB pathway, while H_2_S has the opposite effect. Furthermore, H_2_S promotes the activation of Nrf2. Created with BioRender.com

**Fig. 4:**
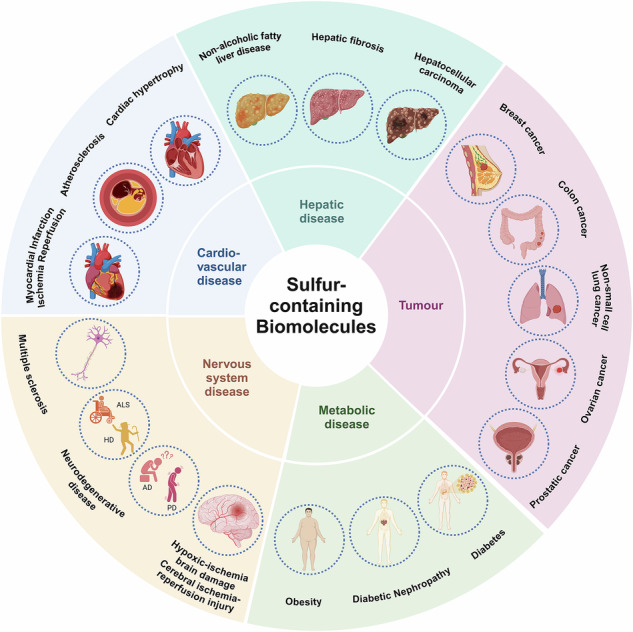
Role of sulfur-containing biomolecules in cardiovascular diseases, liver diseases, brain-related diseases, metabolic disease and tumors. In cardiovascular diseases, they are implicated in conditions such as myocardial infarction ischemia-reperfusion, atherosclerosis, and myocardial hypertrophy. In liver diseases, they are involved in the pathogenesis of non-alcoholic fatty liver disease, liver fibrosis, and hepatocellular carcinoma. Sulfur-containing molecules also contribute to brain-related diseases, including hypoxic-ischemic brain injury, neurodegenerative diseases like Alzheimer’s disease (AD), Parkinson’s disease (PD), Huntington’s disease (HD), and amyotrophic lateral sclerosis (ALS), as well as multiple sclerosis. In metabolic diseases, they are involved in obesity, diabetes, and diabetic nephropathy. Furthermore, sulfur-containing biomolecules have been linked to various cancers, including non-small cell lung cancer, breast cancer, colon cancer, ovarian cancer, and bladder cancer. Created with BioRender.com

The surviving heart after myocardial infarction (MI) undergoes continuous changes with myocardial fibrosis as the main pathological process.^183–185^ Early myocardial fibrosis can reduce expansion of the infarcted area and prevent ventricular rupture; however, prolonged fibrosis can lead to stiffening of the ventricular wall, progressive impairment of heart function, and eventually heart failure.^186^ Studies have shown that feeding mice a high L-methionine diet can induce hyperhomocysteinemia, increase MI risk, and promote myocardial fibrosis and cardiomyocyte apoptosis.^187^ The underlying mechanism involves the propensity of Hcy to undergo auto-oxidation, generating reactive oxygen species (ROS), which can reduce plasma membrane fluidity and compromise cellular integrity. This results in structural and functional damage to cells. The SAM/SAH ratio is critical for the regulation of methylation. In MI models, SAM concentration and the SAM/SAH ratio decrease progressively in a time-dependent manner, offering new insights into the potential pathophysiological mechanisms underlying myocardial infarction.^188^ However, the concentration changes of SAM and SAH are often measured at specific time points following myocardial infarction, which may overlook the impact of long-term and chronic changes on DNA methylation and myocardial repair processes. Moreover, it remains unclear whether alterations in the SAM/SAH ratio have a sustained effect on cardiac function and long-term prognosis. Notably, H_2_S can mitigate myocardial fibrosis following MI. In myocardial tissue from MI rat models, myocardial fibrosis develops, with reduced expression of CSE and downregulation of endogenous H_2_S levels. H_2_S intervention can inhibit excessive activation of the endoplasmic reticulum stress-autophagy axis, activate the PI3K/AKT pathway, reduce apoptosis, and thereby improve myocardial remodeling^189^ (Fig. 3). Overexpression of CSE can similarly inhibit endoplasmic reticulum stress and activate the PI3K/AKT signaling pathway.^190^ The mitochondria-targeted H_2_S donor AP39 can restore mitochondrial H_2_S homeostasis in MI rats, improve myocardial fibrosis and cardiac function, inhibit PINK1 expression and mitophagy, and reduce ROS production and iron accumulation, thereby counteracting ferroptosis in cardiomyocytes.^191^ GSH plays a crucial role in counteracting ROS production and ferroptosis. The key functional component of GSH is the thiol (-SH) group of cysteine, which allows GSH to be oxidized into glutathione disulfide (GSSG) to directly neutralize free radicals and peroxides that accumulate in cells during oxidative stress, thereby exerting a protective effect in MI^192,193^ (Fig. 4). It is important to note that although H_2_S may be involved in myocardial protection through pathways such as the PI3K/AKT signaling pathway, and mitochondrial autophagy, existing studies show inconsistent results and lack systematic validation. Additionally, many studies focus primarily on short-term effects, overlooking the long-term impact of H_2_S after myocardial infarction and its potential for clinical translation.

Myocardial ischemia-reperfusion (I/R) is the restoration of coronary artery blood flow after a period of coronary artery occlusion.^194^ Reperfusion has the potential to save ischemic myocardium. However, the influx of oxygen during reperfusion prompts reactivation of the aerobic metabolic electron transport chain, disrupting the dynamic balance between endogenous pro-oxidant and antioxidant functions.^195–197^ This leads to a significant increase in oxidative stress in vivo, which induces a pro-inflammatory immune cascade response, ultimately resulting in myocardial cell damage and death.^198–200^ Recent studies have identified a specific type of oxidative stress termed disulfide stress, which arises from the abnormal accumulation of intracellular disulfides, such as cystine. SLC7A11 is a cystine/glutamate antiporter that transports extracellular cystine into cells, where it is reduced to cysteine through NADPH consumption. Cysteine serves as the rate-limiting precursor for GSH synthesis, and GSH is a critical intracellular antioxidant. In cancer cells overexpressing SLC7A11 under glucose-deprived (NADPH-reduced) conditions, abnormal accumulation of cystine or other disulfide molecules can occur, triggering disulfide stress and leading to cell death. This novel form of cell death is termed disulfidoptosis.^201^ I/R injury can enhance protein glutathionylation, thereby triggering intracellular disulfide stress.^202^ Glutathionylation is the formation of a disulfide bond between a protein and GSH, serving as a critical redox regulatory mechanism. Under normal physiological conditions, glutathionylation protects protein cysteine residues from hyperoxidation, thereby preserving their structural integrity and functional capacity. However, under pathological conditions, excessive glutathionylation can reduce the activity of antioxidant enzymes, weaken cellular antioxidant defenses, and promote intracellular disulfide stress.^203^ Following myocardial I/R injury, persistent glutathionylation triggers disulfide stress, exacerbating cardiomyocyte damage and ultimately leading to cell death. This novel form of cell death offers new therapeutic insights for treating I/R injury.

Additionally, sulfur-containing biomolecules are also involved in the regulation of I/R injury. Research^204^ has shown that Hcy can activate ERK1/2 pathway (Fig. 3) and oxidative stress in rats subjected to I/R injury, leading to mitochondrial dysfunction and subsequent cardiac dysfunction. Furthermore, a methionine-restricted diet can alleviate myocardial injury induced by I/R; this dietary intervention increases H_2_S concentrations in myocardial tissue and peripheral blood of I/R mice, thereby reducing cardiomyocyte apoptosis.^205^ GYY4137 is a H_2_S donor.^206^ Under physiological pH and temperature, GYY4137 has the ability to gradually release small amounts of H_2_S in water for an extended period, replicating the release pattern of H_2_S in the human body. GYY4137 can protect heart function and reduce the infarct area after myocardial I/R. GYY4137 can protect heart function and reduce the infarct area after myocardial I/R,^207^ while I/R rats with CSE knockout experience exacerbated oxidative stress damage.^208^ This suggests that exogenous H_2_S supplementation or promotion of endogenous H_2_S production can exert cardioprotective effects. Mechanistically, H_2_S supplementation reduces serum malondialdehyde and myeloperoxidase levels after I/R, diminishes superoxide anion levels, inhibits myocardial mitogen-activated protein kinases (MAPK) signaling pathways, and alleviates systemic oxidative stress. Moreover, it can lower Bax expression, caspase-3 activity, and apoptosis^209^ (Fig. 3). H_2_S reduced infarction after I/R by stimulating adenosine monophosphate (AMP)-activated protein kinase and restoring autophagic flux, which in turn providing protection against myocardial I/R injury^210^ (Fig. 4).

Atherosclerosis involves endothelial dysfunction and vascular inflammation, leading to lipid accumulation and plaque formation on arterial walls, ultimately causing rupture and thrombotic events.^211–213^ A methionine-deficient diet leads to hepatic lipid accumulation, a well-known risk factor for atherosclerosis.^214^ Decreased SAM levels or increased SAH levels can both promote the progression of atherosclerosis. SAM supplementation can inhibit the proliferation and migration of vascular smooth muscle cells (VSMCs) by reducing inflammatory processes and endoplasmic reticulum oxidative stress. It also reduces carotid intima thickness and prevents endothelial dysfunction by inducing heme oxygenase-1 expression. In contrast, elevated plasma SAH levels promote VSMC proliferation and migration via activation of the oxidative stress-mediated ERK signaling pathway and activate endothelial cell inflammation through the NF-κB signaling pathway.^215^ SAM/SAH regulate processes related to atherosclerosis through multiple signaling pathways; however, these pathways may involve complex cross-talk and feedback mechanisms. Current studies tend to focus on individual signaling pathways, often overlooking the interactive effects between these pathways. High concentrations of Hcy can exacerbate the onset and progression of atherosclerosis in patients with systemic lupus erythematosus.^216^ Supplementation of H_2_S has shown improvement in atherosclerosis, while inhibiting CSE activity or having CSE gene defects can lower endogenous H_2_S levels and accelerate the progression of atherosclerosis.^217^ Liu^218^ designed and synthesized H_2_S donors modified with either niacin or clofibrate. All three H_2_S donors reduced the expression of apoptosis-related proteins Bax and caspase-3, exhibited antioxidant effects (significantly decreased ROS and MDA levels, while increasing SOD expression), and inhibited inflammatory responses (suppressing foam cell inflammation, reducing pro-inflammatory cytokine TNF-α, and increasing anti-inflammatory cytokine IL-10). Further studies revealed that these H_2_S donors inhibit the PI3K/Akt/NF-κB signaling pathway, thereby improving vascular function and suppressing atherosclerosis (Fig. 3). Similarly, NaHS, as an H_2_S donor, can improve vascular dysfunction, reduce the area of aortic atherosclerotic lesions and inhibit their progression by inhibiting the production of vascular superoxide.^219^ During the development of atherosclerosis, not only do plaques change, but the media and external elastic lamina of arteries continuously expand or contract, leading to changes in vascular lumen and causing vascular remodeling. Endogenous H_2_S protects vascular remodeling by maintaining the peroxisome proliferator activated receptor delta/suppressor of cytokine signaling 3 (PPARδ/SOCS3) anti-inflammatory signaling pathway. The lack of endogenous H_2_S results in vascular remodeling, thickening of the aortic wall, collagen deposition, increased phosphorylation of signal transducer and activator of transcription 3 (STAT3), reduced generation of aortic PPARδ and SOCS3. Therefore, the lack of endogenous H_2_S may be a risk factor for atherosclerosis and vascular remodeling^220^ (Fig. 4). Most current studies focus on the short-term effects of H_2_S donors, without thoroughly exploring their long-term impact on atherosclerosis progression, vascular remodeling, and clinical outcomes. Long-term effects may involve complex adaptive changes that have not been fully addressed. Additionally, the influence of different doses and administration methods on H_2_S donor efficacy remains underexplored. Varying doses may elicit distinct biological effects, and either excessive or insufficient doses could alter therapeutic outcomes or cause side effects.

The initial hypertrophy of the heart is a compensatory response to a failing heart, increasing contractility and reducing ventricular wall pressure in response to hemodynamic overload, but sustained hypertrophy can lead to cardiac dilation, loss of contractile function, and decreased ejection fraction, ultimately leading to heart failure.^221–223^ Elevated levels of Hcy can promote cardiac hypertrophy in patients with hypertension, with the calcium/calmodulin-dependent protein kinase-NFAT signaling pathway potentially involved in Hcy-induced hypertrophy.^224^ H_2_S is a product generated from Hcy via the transsulfuration pathway, and its donors can mitigate Hcy-induced cardiomyocyte hypertrophy.^225^ The protective role of H_2_S in pathological myocardial hypertrophy is increasingly being confirmed. In a myocardial hypertrophy model established by isoproterenol (ISO) injection, H_2_S decreased the expression of cleaved caspase-3 and NADPH oxidase 4 (NOX4), inhibited cardiac cell apoptosis, and improved cardiac structure. Furthermore, H_2_S can maintain mitochondrial membrane potential, and decrease the production of ROS within the mitochondria.^226^ However, this model differs from human pathology, particularly in long-term pathological conditions or clinical contexts, where the translational relevance of animal models is limited. The hypertrophic signaling pathway activated by myocardial infarction is defective in CSE knockout mice. However, 2 h after the onset of myocardial infarction, the treatment of both CSE knockout mice and wild-type mice with the exogenous H_2_S donor GYY4137 reduce infarct size, myocardial hypertrophy, adverse remodeling, and preserve cardiac function.^227^ An age-dependent association between 3-MST and cardiac hypertrophy has been found in mice. Knockout of 3-MST has a cardiac protective effect in young adult animals (2–3 months old); however, in older mice (>18 months), 3-MST knockout leads to reduced antioxidant signaling and subsequent hypertension and cardiac hypertrophy.^228^ In addition, H_2_S can increase glucose uptake and the expression of the glucose transporter glucose transporters type 4 (GLUT4) in hypertrophic cardiomyocytes, while inhibition of GLUT4 in mice can worsen myocardial hypertrophy.^229^ Notably, the accumulation of SAH in the heart reflects the concentration of free cytosolic adenosine and serves as a sensitive indicator of localized myocardial ischemia.^230^ In adult mice, knockout of mitochondrial Rieske iron-sulfur protein (RISP) leads to an increase in SAM, which is necessary for methyltransferase activity, resulting in proliferative remodeling of the heart with a doubling of cardiomyocyte numbers, although cellular hypertrophy does not occur^231^ (Fig. 4).

### Liver disease

NAFLD is widely assumed as the most common chronic liver disease, and the interaction between lipid metabolism disorders and the resulting inflammatory response can jointly promote the occurrence and development of NAFLD.^232–234^ It has been found that,^235^ in the livers of NAFLD patients, especially in fibrosis areas, the expression of CSE protein was significantly downregulated. The CSE/H_2_S pathway was also downregulated in high-fat diet (HFD)-induced NAFLD mice or oleic acid-induced liver cell models. Feeding CSE knockout mice with HFD increased liver lipid deposition, fatty acid de novo synthesis pathway activity, liver insulin resistance, and enhanced hepatic gluconeogenesis, while treatment with an H_2_S donor attenuated these phenotypes. The protective effect of H_2_S was blocked when farnesoid X receptor (FXR) was knocked down. Furthermore, CSE/H_2_S promoted the persulfidation of FXR at Cys138/141 locations, consequently boosting its effectiveness in regulating the expression of target genes associated with lipid-glucose metabolism, inflammation, and fibrosis, thus alleviating NAFLD. In an HFD-fed SD rat model, the H_2_S donor AP39 reduced weight gain, improved HFD-induced liver pathology, and reduced serum lipid accumulation. AP39 also exhibited antioxidant effects by reducing ROS and MDA levels, increasing GSH levels, and superoxide dismutase (SOD) activity. In addition, AP39 reduced both mRNA and protein levels of HIF-1α, decreased mitochondrial swelling, and restored changes in mitochondrial membrane potential.^236^ In addition to the commonly used HFD models, there are also animal models of methionine-choline deficient (MCD) diet.^237–239^ Methionine and choline play critical roles in hepatic lipid metabolism. When these nutrients are deficient, they can cause dysregulation of lipid metabolism, oxidative stress, and inflammatory responses, ultimately leading to hepatic fat accumulation and hepatocyte injury. Deficiencies in methionine and cystine can induce non-alcoholic steatohepatitis (NASH), which is characterized primarily by steatosis, oxidative injury, and inflammation.^240^ Prolonged deficiencies in methionine and cystine lead to reduced protein synthesis and increased oxidative stress. The heightened oxidative stress in the liver results in mitochondrial damage, which is considered a trigger for the pathogenesis of NASH. SAM is most abundant in the liver, and its biosynthesis requires methionine adenosyltransferase (MAT), with MAT1A expressed in normal mature liver and MAT2A expressed in extrahepatic tissues, induced during liver growth and dedifferentiation.^241^ In fatty liver disease, alterations in the methionine cycle leads to reduced expression of MAT1A and increased expression of MAT2A, resulting in decreased SAM levels and contributing to the development of NAFLD.^242^ Plasma methionine levels are significantly elevated in MAT1A knockout mice, while hepatic SAM and glutathione levels are notably decreased, with no change in SAH levels. These mice are more susceptible to liver damage and more vulnerable to fat accumulation induced by a choline-deficient diet^243^ (Fig. 4). Although the HFD and MCD models are widely used in NAFLD research, these animal models differ from the pathological processes observed in human NAFLD. For instance, the HFD model primarily induces fat accumulation and insulin resistance, while the MCD model results in more pronounced hepatic fibrosis. Therefore, these animal models may not fully recapitulate the complexity of human NAFLD, limiting the translational relevance of the findings.

Liver fibrosis is a wound healing response following liver damage (like NAFLD and hepatitis). Liver fibrosis is linked to oxidative stress, inflammation, and excessive deposition of extracellular matrix (ECM), and can eventually develop into cirrhosis.^244–246^ Serum Hcy levels are positively correlated with the progression of liver fibrosis, potentially exerting their effects through the homocysteinylation of the autophagosome/lysosome fusion protein Syntaxin 17 (Stx17).^247^ Folic acid can protect the liver from cholestasis and liver fibrosis by reducing serum Hcy levels and exerting its antioxidant properties.^248^ In patients with liver fibrosis, the expression and activity of H_2_S-generating enzymes and plasma H_2_S levels are significantly lower than in the healthy group.^249^ In a mouse model, H_2_S can improve liver damage, lower serum alanine transaminase (ALT) and aspartate transaminase (AST) levels, reduce lipid deposition, and decrease liver cell death. Moreover, H_2_S induces sulfhydrylation of Kelch-like ECH-associated protein 1 (Keap1) at Cys151, promotes its association with (NF-E2)-related factor 2 (Nrf2), and increases the expression of Nrf2-associated antioxidant genes in vivo and in vitro, thereby improving liver function and reducing liver fibrosis.^250^ S-allyl-cysteine (SAC) is an endogenous donor of H_2_S, which can alleviate carbon tetrachloride (CCL4)-induced liver fibrosis in rats, reduce the mRNA expression of inflammatory and fibrotic cytokines, and increase antioxidant enzyme activity. SAC lowered the phosphorylation levels of Smad family member 3 (Smad3) and STAT3, inhibited their binding ability to the transcription promoter, thereby restraining the transcription of fibrosis-related genes and causing the expression of antioxidant-related genes^251^ (Figs. 3, 4). The mechanisms of H_2_S in liver fibrosis remain incomplete. While it inhibits fibrosis by activating the Keap1-Nrf2 pathway and downregulating the phosphorylation of Smad3 and STAT3, the interactions among involved signaling pathways (such as PI3K/Akt, MAPK, TGF-β, Wnt/β-catenin) and their roles at different pathological stages have not been systematically studied. The complexity of these mechanisms limits the clinical application of H_2_S in liver fibrosis treatment and warrants further investigation.

### Brain-related diseases

The pathophysiological process of hypoxic-ischemic (HI) brain injury involves multiple mechanisms, including synaptic injury,^252^ inflammation,^253^ and oxidative stress.^254^ SAM can exacerbate hypoxic-ischemic injury in cortical cells of rats.^255^ Hcy can increase damage in the hippocampus of ischemic-hypoxic models in rats, which is consistent with the heightened susceptibility of patients with hyperhomocysteinemia to ischemic events.^256^ Increased cystine uptake and elevated extracellular glutamate levels can enhance hypoxic neuronal injury in cortical cultures from mice.^257^ Latest studies have shown that the expression of CBS and H_2_S levels in samples from HI patients and animals were significantly decreased.^258^ L-Cys, through its ability to generate H_2_S, can reduce early brain injury in HI, improve behavioral deficits, and synaptic injury. Treatment with L-Cys reduced the accumulation of CD11b^+^/CD45^high^ cells, the activation of microglia and astrocytes, and the increase in ROS and MDA within the injured cortex. It is hypothesized that H_2_S may be effective in attenuating HI injury by inhibiting reactive glial responses, synaptic modifications, and the triggering of autophagic fluxes^259^ (Fig. 4). Although L-Cys can inhibit the excessive activation of microglial cells and astrocytes, the effects of H_2_S on immune cell function are complex. Microglial cells not only play a role in inflammation but also contribute to neural repair and regeneration. In certain contexts, H_2_S may interfere with the normal functioning of microglial cells, potentially impairing their reparative functions.

As a result of cerebral ischemia, cerebral ischemia-reperfusion injury (CIRI) is a pathological condition characterized by an aggravation of damage once blood flow is restored. It plays an important part in the development and progression of ischemic brain diseases.^260–262^ The occurrence of CIRI is mainly related to neuroinflammation, oxygen free radical damage, autophagy, and calcium overload, ultimately leading to mitochondrial dysfunction, disruption of the blood-brain barrier (BBB), and neuronal death. SAM can inhibit blood-brain barrier disruption and promote neuronal survival following transient cerebral ischemia in gerbils.^263^ Elevated levels of Hcy are associated with neurotoxicity after CIRI. Hcy can enhance autophagy mediated by oxidative damage, thereby promoting cell death following cerebral ischemia.^264,265^ In contrast, SAM can reduce oxidative damage in rat models of cerebral ischemia-reperfusion.^266^ Notably, low concentrations of H_2_S can exert protective effects in the central nervous system through multiple mechanisms.^25,267^ Studies have reported that NaHS improves neurofunction in rats after transient middle cerebral artery occlusion (MCAO) and reperfusion, reduces the infarct area, and inhibits autophagy activity in the brains of MCAO rats, suggesting that H_2_S can alleviate CIRI in rats by suppressing excessive autophagy activation.^268^ In addition, H_2_S preconditioning prevents neurological dysfunction, inflammation, oxidative damage, and cognitive impairment in mice caused by CIRI, and its protective effect may be achieved through the induction of heat shock protein 70 (HSP70) expression via the PI3K/Akt/Nrf2 signaling pathway.^269^ It has been found that Ras Homolog Family Member A (RhoA) and Rho-associated coiled-coil-containing protein kinase 2 (ROCK2) expression is upregulated in the hippocampal tissue of CIRI mice. ROCK has a significant inhibitory effect on cell survival and axon growth, and the upregulation of ROCK2 is considered a marker of activation of the RhoA/ROCK pathway in the brain. This regulation can be blocked by treatment with the exogenous H_2_S donor NaHS. Subsequent studies confirmed that H_2_S derived from CSE can promote the recovery of neurological function in CIRI mice by inhibiting the RhoA/ROCK2 signaling pathway and suppressing reactive proliferation of astrocytes^270^ (Figs. 3, 4). Although H_2_S can alleviate CIRI by inhibiting excessive autophagy, autophagy plays a dual role within the cell. It is a protective mechanism that, under certain conditions, promotes cell survival by clearing damaged cellular components. However, excessive inhibition of autophagy may lead to the accumulation of cellular debris, which could be detrimental to neuroprotection. Therefore, the modulation of autophagy by H_2_S requires precise regulation in terms of cell type, injury severity, and dosage to avoid potential adverse effects.

H_2_S, as a neuromodulator, plays an important role in regulating neuron health and synaptic structure integrity.^271^ In the adult mouse brain, CBS is ubiquitously expressed, and inhibition or knockout of CBS affects the proliferation and differentiation of neural stem cells, which can be blocked by supplementation with H_2_S donor.^272^ Dysregulation of H_2_S levels is commonly observed in neurodegenerative diseases, indicating the potential therapeutic value of H_2_S in conditions, for instance, Alzheimer’s disease (AD), Parkinson’s disease (PD), Huntington’s Disease (HD) and amyotrophic lateral sclerosis (ALS) (Fig. 4).

AD is characterized by the aggregation of microtubule-associated protein Tau and Aβ peptide, forming neurofibrillary tangles and amyloid plaques, respectively.^273–275^ Hyperphosphorylation of Tau protein, a hallmark of AD, reduces its affinity for microtubules and causes it to aggregate.^276,277^ In AD mouse models, H_2_S donors can alleviate disease symptoms, improve spatial and cognitive deficits in mice. Furthermore, H_2_S prevents the pathological phosphorylation of Tau by inhibiting the catalytic activity of glycogen synthase kinase 3β (GSK3β), one of the main kinases responsible for Tau protein phosphorylation, ultimately exerting neuroprotective effects against AD.^278^ Notably, cerebrospinal fluid levels of SAM and the SAM/SAH ratio, as well as SAM levels in specific brain regions (cerebral cortical subdivisions, hippocampus, and putamen), are significantly decreased in patients with AD compared to controls, likely due to excessive utilization in polyamine biosynthesis.^279^ The reduction in SAM levels may impair metabolic processes and brain function in AD patients.^280^ A randomized controlled trial demonstrated that folic acid supplementation can increase plasma levels of SAM and the SAM/SAH ratio in patients with AD, reduce inflammation, and thereby alleviate AD symptoms.^281^ SAM and superoxide dismutase 1 (SOD1) can synergistically counteract the exacerbation of AD-like features caused by B-vitamin deficiency, suggesting that the combination of SAM and SOD1 may serve as a potential adjunctive therapy for AD. However, current studies may be limited by small sample sizes and insufficiently accurate dosage stratification. Further research is needed to explore and validate these findings in greater depth.

PD is caused by progressive degeneration of dopaminergic cells in the substantia nigra, which is the second most common neurodegenerative disease after AD.^282–284^ Abnormal protein handling, excitotoxicity, neuroinflammation, and apoptosis can all contribute to the development of PD. In a mouse model of PD caused by the neurotoxin 1-Methyl-4-phenyl-1,2,3,6-tetrahydropyridine (MPTP),^285^ inhalation of H_2_S can prevent MPTP-induced motor impairments and the degeneration and apoptosis of tyrosine hydroxylase (TH)-containing neurons. Additionally, H_2_S can increase the expression of detoxifying enzymes and antioxidant proteins in the brain’s substantia nigra, suggesting that H_2_S can mitigate PD pathology by upregulating antioxidant defense mechanisms and suppressing inflammation and cell apoptosis in the brain.^286^

HD is a progressive neurodegenerative disorder characterized by motor, cognitive, and psychiatric symptoms. The Huntingtin gene encodes a protein known as “huntingtin”, but this gene contains an expanded CAG repeat sequence, leading to the production of an abnormal protein.^287^ The aggregation of mutant huntingtin protein disrupts numerous cellular processes, including transcription and translation regulation,^288,289^ amino acid homeostasis,^290^ antioxidant and stress responses,^291,292^ DNA repair, and autophagy.^293–295^ Among these processes, oxidative stress plays a crucial role, with H_2_S exerting antioxidant effects in HD by activating antioxidant enzymes to limit free radical reactions.^296^ In HD tissues, there is a deficiency in CSE mRNA levels, and cysteine supplementation has been shown to alleviate abnormalities in HD mouse models, suggesting its therapeutic potential.^297^ Additionally, Hcy levels are significantly elevated in HD patients, indicating that Hcy may contribute to neurodegeneration in HD.^298^

Notably, Hcy levels are also elevated in ALS patients, suggesting that higher Hcy levels may be associated with the progression of ALS.^299^ ALS is caused by the degeneration of motor neurons, leading to muscle atrophy, paralysis, and ultimately death.^300^ Similar to HD, oxidative stress also plays a significant role in ALS. SOD1 is located in the mitochondrial outer membrane, intermembrane space, and inner membrane. Mutations in SOD1 are considered oxidative stress-inducing factors in ALS pathogenesis. H_2_S has shown therapeutic potential for ALS by inhibiting SOD1 aggregation and countering oxidative modifications.^301^ Dietary supplementation with SAM also impacts SOD1. SAM supplementation can delay ALS onset in mouse models by 2–3 weeks and mitigate neurodegenerative characteristics, including preventing motor neuron loss, reducing gliosis, and inhibiting SOD1 aggregation.^302^ SOD1 mutations are considered an oxidative stress-inducing factor in the pathogenesis of ALS. However, not all ALS patients harbor SOD1 mutations, and these mutations account for only a small fraction of ALS cases. Therefore, the findings from studies on SOD1 mutations may not be generalizable to all ALS patients.

Multiple sclerosis (MS) is an inflammatory disease of the central nervous system, affecting motor, sensory, visual, and autonomic systems.^303–305^ Studies have shown that methionine and glutathione levels are decreased in MS patients, both of which may serve as potential biomarkers for disease prognosis.^306^ However, Hcy is more commonly considered a potential indicator of MS progression.^307,308^ Those abovementioned findings suggest that sulfur-containing compounds are broadly involved in the pathogenesis of MS. NaHS, as an H_2_S donor, has shown potential therapeutic effects in the progression of MS. Studies indicate that^309^ NaHS reduces the expression of IRAK-1, NF-κB, and brain levels of IL-17 and IL-1β, thereby improving motor dysfunction in MS mice, reducing axonal demyelination, oxidative stress, and neuroinflammation. The role of H_2_S has also been validated in MS patients, as evidenced by the downregulation of 3-MST expression in peripheral blood mononuclear cells, with 3-MST expression inversely correlated with several pro-inflammatory cytokines.^310^ Additional evidence suggests that H_2_S can inhibit the production of inflammatory mediators by immune cells such as T cells and macrophages, highlighting its therapeutic potential in MS treatment^311^ (Figs. 3, 4).

Notably, in the cerebellum, the D-Cys pathway predominates and produces more H_2_S and protects cerebellar neurons from oxidative stress more effectively than the L-Cys pathway.^129^ It has been shown,^312^ that D-Cys promotes dendritic development in Purkinje cells. However, the promotion of D-Cys was inhibited by the administration of a DAO inhibitor, and its effects could be subsequently restored by treatment with donors of 3-MP and H_2_S. These results indicate that D-Cys promotes dendritic development of primary cultured Purkinje cells through the production of H_2_S, which also suggests that this pathway could be a novel therapeutic direction for cerebellar diseases.

### Metabolic disease

H_2_S-generating enzymes are also present in endocrine glands and organs.^313–315^ Studies indicate that CBS mRNA and protein expression levels are highest in the pancreas, particularly within acinar cells.^316^ Similarly, 3-MST is highly expressed in tissues such as the thyroid, parathyroid, adrenal glands, and pancreas.^317^ Consequently, H_2_S plays a significant role in lowering blood glucose. Plasma H_2_S levels are notably decreased in patients with type 2 diabetes, paralleling poor glycemic control^318^ (Fig. 4). In diabetic rat models, fasting blood glucose levels are inversely correlated with plasma H_2_S levels and H_2_S synthesis activity. Additionally, both plasma H_2_S levels and H_2_S synthesis activity are significantly reduced in diabetic rats.^319^ Notably, H_2_S can also improve left ventricular function, preventing myocardial hypertrophy and fibrosis in diabetic rats. Its protective mechanism may involve the activation of the Nrf2/ARE and PI3K/Akt pathways, thereby reducing inflammation, oxidative stress, and apoptosis, which helps mitigate the progression of diabetic cardiomyopathy.^320^ Additionally, elevated levels of Hcy are considered a potent contributor to chronic complications in diabetes.^321^ Hyperhomocysteinemia is an independent risk factor for the development and progression of diabetic retinopathy, whereas Hcy levels below a specific serum threshold serve as a protective factor for diabetic retinopathy.^322^ However, it is worth noting that lower Hcy levels are not necessarily better, as excessively low Hcy can indicate deficiencies in other substances, such as vitamins. In the future, it is also essential to monitor changes in other biomarkers reflected behind low Hcy levels.^323^ The measurement of Hcy levels may be influenced by various factors, including renal function, medications, gender, and age. These factors can lead to fluctuations in Hcy levels, potentially affecting its accuracy as a predictive marker for diabetic complications.

Diabetic kidney disease (DKD) is a form of chronic kidney disease caused by diabetes mellitus and is one of the leading causes of end-stage renal disease.^324–326^ Studies indicate that, as the disease progresses, SAH levels in red blood cell increase, but SAM levels and SAM/SAH ratio decrease. SAM deficiency may lead to methyl deficiency, which is associated with the high incidence and mortality of DKD patients.^327^ Glycine N-methyltransferase (GNMT), a SAM-dependent enzyme, plays a critical role in methyl transfer reactions by regulating the cellular SAM/SAH ratio. Research shows that GNMT expression is significantly downregulated in the serum of patients with type 1 diabetes and in the kidney tissues of DKD mice. GNMT overexpression alleviates renal inflammation and fibrosis, presenting a new therapeutic target for DKD.^328^ Although GNMT is considered a novel therapeutic target for DKD, treatment strategies targeting GNMT may need to account for individual differences, such as genetic polymorphisms, disease stage, and comorbidities, all of which could influence treatment outcomes. Moreover, H_2_S plays a significant role in diabetic nephropathy. Increasing H_2_S levels has been shown to mitigate renal dysfunction and pathological changes in diabetic rats. The protective effect of H_2_S against diabetic nephropathy may be associated with a reduction in oxidative stress through enhanced antioxidant activity.^329^ SIRT1 is considered an anti-aging molecule that utilizes the coenzyme NAD^+^ to deacetylate target proteins, thereby exerting protective effects in the kidneys by inhibiting renal cell apoptosis,^330^ inflammation^331^ and fibrosis,^332^ ultimately slowing the progression of DKD. H_2_S can upregulate SIRT1 expression, reduce ROS, and inhibit apoptosis, thus protecting renal cells from further DKD-related damage.^333^ Moreover, H_2_S can lower blood pressure in spontaneously hypertensive diabetic rats, alleviate renal dysfunction, and inhibit the progression of early DKD.^334^ Thus, sulfides exhibit a mitigating effect on diabetes and its complications, offering new insights for the prevention and treatment of these conditions (Fig. 4).

Obesity is closely associated with type 2 diabetes, and both conditions represent major global health burdens. The prevalence of obesity has shown a sharp increase over the past few years.^335–337^ Research has shown that plasma total cysteine levels are positively correlated with obesity, particularly with adipose tissue mass.^338^ In several rodent models, increased dietary cysteine levels have been associated with increased obesity.^339^ However, there is also evidence suggesting that cysteine can reduce appetite in both humans and rodents,^340^ which contradicts its role in promoting adiposity. Therefore, a deeper understanding of the additional mechanisms by which cysteine influences metabolic control is warranted. In addition to focusing on cysteine itself, its metabolic product, H_2_S, is also critical in the context of obesity. H_2_S levels are lower in obese individuals. Plasma H_2_S shows a negative correlation with obesity, such as waist circumference and waist-to-hip ratio.^341^ However, it is important to note that H_2_S has a complex role in both lipogenesis and lipolysis. H_2_S promotes the differentiation of preadipocytes into adipocytes by activating a series of transcription factors, including peroxisome proliferator-activated receptor gamma (PPAR-γ), CCAAT/enhancer-binding protein alpha (C/EBPα), sterol regulatory element-binding protein 1 (SREBP1), and carbohydrate response element-binding protein (ChREBP), thereby increasing triglyceride accumulation..^342^ In comparison to wild-type mice, the knockout mice for CBS and CSE exhibit significantly reduced adipose tissue mass and decreased body weight.^343,344^ The role of H_2_S in regulating lipolysis in adipose tissue is contentious. The CSE/H_2_S pathway inhibits lipolysis via the protein kinase A-perilipin/hormone-sensitive lipase pathway while simultaneously reducing high-fat diet-induced insulin resistance.^345^ However, other studies indicate that H_2_S can stimulate lipolysis in adipose tissue in a cAMP-PKA-dependent manner. Upregulation of the CSE/H_2_S pathway in adipose tissue may facilitate lipolysis in animals fed high-fat diets.^346^ Therefore, the controversial role of H_2_S necessitates further exploration to provide stronger evidence for the perspective that H_2_S donors or enhancers of H_2_S signaling may improve adipose tissue dysfunction in common metabolic disorders (Fig. 4).

### Tumor

Sulfur-containing biomolecules^347^ are involved in a variety of processes associated with tumor progression, including angiogenesis, tumor growth, cell migration, invasion and metastasis, epithelial-mesenchymal transition, energy metabolism in mitochondria, and chemoresistance.^348–351^ Sulfur-containing biomolecules have recently been well studied in the pathogenesis of hepatocellular carcinoma (HCC), breast cancer, colon cancer, lung cancer, pancreatic cancer, ovarian cancer and prostate cancer (Fig. 4).

HCC is the most common type of liver cancer and is associated with changes in cell proliferation, oxidative stress, and inflammatory responses.^352–354^ The effects of H_2_S on HCC are complex and can promote or inhibit the development of HCC by modulating different cell signaling pathways. It has been found that,^355^ low concentrations of H_2_S stimulate the growth of cancer cells. Treatment with low concentrations of NaHS (10-100 µM) increases the protein levels of phosphorylated-epidermal growth factor receptor (p-EGFR), phosphorylated-extracellular signal-regulated protein kinases (p-ERK), matrix metalloproteinase 2 (MMP-2), and p-Akt, which could activate the EGFR and its downstream signaling pathways to promote HCC proliferation and invasion (Fig. 3). On the other hand, high concentrations of H_2_S have anti-tumor effects on cancer cells. Treatment with high concentrations of NaHS (600-1,000 µM) inhibits the (phosphatase and tensin homolog) PTEN/Akt signaling pathway, thereby inhibiting angiogenesis and tumor growth without causing significant systemic toxicity. However, some studies have shown that^356^ extremely low concentrations of NaHS (1–10 µM) can also inhibit HCC cell migration, proliferation, and division. H_2_S can upregulate the expression of LC3-II and autophagy-related protein Atg5, two autophagy-related proteins, in HepG2 cells, while significantly inhibiting the expression of p-PI3K, p-Akt, and mammalian target of rapamycin (mTOR) proteins in liver cancer cells. This indicates that extremely low concentrations of H_2_S can inhibit HCC by promoting autophagy and suppressing apoptosis. High concentrations of H_2_S also exhibit dual effects.^357^ Treatment with 500 µM NaHS for 24 h enhances cell viability and migration ability, while reducing the number of apoptotic cells. The levels of p-STAT3 and STAT3 significantly increase, leading to overexpression of cyclooxygenase-2 (COX-2) and COX-2 mRNA, indicating that H_2_S can decrease cell apoptosis through the STAT3/COX-2 signaling pathway and promote the progression of HCC. These findings demonstrate the complexity and environment-dependent role of the H_2_S signaling pathway in the occurrence and development of HCC, and further studies are needed to fully understand the molecular mechanisms underlying the dual effects of H_2_S and develop targeted and efficient anti-cancer therapies. Moreover, studies have shown that SAM is significantly reduced in HCC, consequently impacting critical metabolic pathways, including transmethylation reactions, the methionine cycle, and trans-sulfuration pathways.^358^ The gluconeogenic enzyme phosphoenolpyruvate carboxykinase 1 (PCK1) can promote SAM production. PCK1 deficiency exacerbates HCC, potentially exerting its tumor-promoting effects by upregulating the PI3K/AKT signaling pathway. Furthermore, both in vivo and in vitro supplementation of SAM has been shown to inhibit the progression of HCC caused by PCK1 deficiency. This suggests that SAM may act as a bridge linking PCK1 and PI3K, playing a beneficial role in the progression of HCC.^359^ Importantly, disulfidptosis, as a novel form of cell death, has significant implications for clinical prognosis in HCC. Researchers have analyzed patient samples to develop a disulfidptosis risk score, with higher scores correlating with increased mortality risk. This finding suggests that novel biomarkers associated with disulfidptosis could serve as valuable tools in the clinical diagnosis, prognostic prediction, and therapeutic targeting of HCC.^360^ The development of a disulfidptosis risk score, while promising, may oversimplify the complex, multifactorial nature of HCC. Tumor progression and patient prognosis depend on various genetic, epigenetic, and microenvironmental factors, many of which may not be fully captured by a single risk score based solely on disulfidptosis. Therefore, relying too heavily on this score could lead to an incomplete understanding of a patient’s prognosis, potentially overlooking other important factors.

SAM has also demonstrated similar effects in breast cancer. A preclinical study indicated that the combination of SAM and decitabine shows significant potential in the anticancer treatment of breast cancer.^361^ SAM enhances the levels of autophagy markers beclin-1 and LC3B-II in MCF-7 breast cancer cells and significantly increases the Bax/Bcl-2 ratio, indicating that SAM can inhibit breast cancer by inducing autophagy and apoptosis in MCF-7 cells.^362^ In contrast to SAM, elevated levels of Hcy are considered a risk factor for cancer and a potential novel tumor marker.^363^ Women with high levels of Hcy and cysteine who also have low folate levels are at an increased risk of developing breast cancer.^364^ However, factors such as gender, ethnicity, and individual genetic background may influence the expression and function of these biomarkers. For example, folate absorption and metabolism can vary across different populations, which suggests that relying solely on these biomarkers to assess breast cancer risk may have limitations and may not be universally applicable to all populations. H_2_S plays a dual role in breast cancer. Wu^365^ found that inhibiting endogenous H_2_S can reduce the vitality, proliferation, migration, and invasion rate of human breast cancer cells, induce apoptosis in human breast cancer cells, and decrease the phosphorylation levels of PI3K, Akt, and mTOR. In an animal model of human breast cancer xenografts, it was found that inhibiting H_2_S can reduce the generation and growth of tumor blood vessels. This indicates that H_2_S acts on the PI3K/Akt/mTOR pathway in human breast cancer cells, and inhibiting the production of endogenous H_2_S can reduce cell proliferation and tumor growth through this signaling pathway. Triple-negative breast cancer (TNBC) is the most aggressive subtype of breast cancer, currently lacking targeted therapy.^366–368^ Chemotherapy is the only systemic treatment strategy available for TNBC patients and has a poor prognosis. TNBC cells are also inhibited from growing, proliferating, migrating, and invading when treated with H_2_S inhibitors. Moreover, it also reduces the protein levels of PI3K, Akt, mTOR, and Ras, Raf, and p-ERK, indicating that H_2_S may regulate human TNBC cells through dual targeting of the PI3K/Akt/mTOR and Ras/Raf/ERK signaling pathways^369^ (Fig. 3). Nevertheless, some studies have also shown that H_2_S donor can inhibit tumor invasion and metastasis. During the metastatic process, matrix metalloproteinases (MMP-2 and MMP-9) play a crucial role by degrading type IV collagen. Lu^370^ investigated the effects of H_2_S donor on MMPs and found that H_2_S donor can inhibit the viability of TNBC cells, increase apoptosis, and significantly suppress the mRNA levels, protein expression, and enzymatic activity of MMP-2/9 during invasion, tumorigenesis, and metastasis by blocking the NF-κB and ERK/MAPK signaling pathways. Additionally, other studies have shown that H₂S donors and their derivatives can exert strong antitumor effects by inhibiting the aberrant activation of the β-catenin pathway, thereby reducing the expression of MMP-9^371^ (Fig. 3). The contradictory roles of H₂S suggest that further in-depth research is needed to explore the potential mechanisms and specific contexts in which H₂S exerts its effects, thereby providing new therapeutic targets for the treatment of breast cancer patients.

In patients with colon cancer, Hcy levels are similarly elevated, and Hcy levels correlate with the patients’ IL-6, TNF-α, and folate levels. This suggests that Hcy may be associated with inflammation related to colon cancer, potentially involving TNF-α-mediated pathways.^372^ 5-MTHF is a key metabolic product in the folate and homocysteine metabolism pathways. In colon cancer cells treated with Hcy, both folate and 5-MTHF can reverse the growth enhancement induced by Hcy and inhibit the excessive proliferation of colon cancer cells, thus providing a protective effect.^373^ Epithelial-mesenchymal transition (EMT) is a major pathological change in colon cancer, representing the process of transition from a normal (epithelial) state to a transformed (mesenchymal) state.^374–376^ EMT represents a series of complex cellular events, typically controlled by the Wnt/β-catenin signaling pathway.^377^ Epithelial cells thus lose their intercellular adhesion capacity and acquire mesenchymal properties. This process can also occur in the opposite direction, known as mesenchymal-epithelial transition (MET).^378,379^ Research has found that,^380^ H_2_S promoted EMT in human colon cancer HCT116 cells, and inhibiting H_2_S could significantly downregulate Wnt3 mRNA levels and β-catenin protein expression, as well as reduce ATP-citrate lyase (ACLY) mRNA and protein levels. Reports indicate that ACLY is associated with the Wnt signaling pathway and is involved in EMT in colon cancer cell lines.^381^ The ACLY promoter is regulated by Sp1 and Sp3. It has been found that under the action of H_2_S inhibitors, the mRNA levels of Sp1 and Sp3 both decrease, with the effect on Sp3 being more pronounced. This suggests that the mechanism by which H_2_S maintains EMT is related to the regulation of the Sp3-ACLY-Wnt-β-catenin pathway. Comparing the gene expression levels between normal and tumor tissues in colon cancer patients revealed an upregulation of the TRIP6 gene associated with disulfide death. The TTPAL gene can prevent TRIP6 from being degraded by the proteasome and enhance its interaction with β-catenin. This suggests that the TTPAL-TRIP6-β-catenin axis present in disulfidptosis can activate the Wnt/β-catenin pathway, thereby promoting the progression of colon cancer.^382^ One study reported that,^383^ treating HCT116 cells with a low concentration (0.3 mM) of GYY4137 (a slow-release H_2_S donor) increased the proliferation rate of HCT116 cells, while enhancing mitochondrial function and glycolysis, thereby promoting tumor growth. It is worth noting that overexpression of CBS and supplementation of exogenous H_2_S can inhibit the proliferation, colony formation, migration, and hepatic metastasis of colon cancer cells. CD44 and SP-1 may be involved in the inhibitory effect of the CBS/H_2_S on colon cancer cells.^384^ The above findings indicate that the role of H_2_S is biphasic, concentration- and time-dependent. Szabo^385^ evaluated the impact of different concentrations of H_2_S donors on HCT116 proliferation and found that moderate H_2_S stimulation can enhance the proliferation of colon cancer cells, while high concentrations of sustained H_2_S treatment can reduce the proliferation activity of tumor cells. Different H_2_S donors vary in their rates of H_2_S release, stability, and metabolism, which can influence their biological effects in vivo. Additionally, the selectivity of H_2_S donors, individual differences among patients, interactions with other therapeutic modalities, and potential side effects need to be further investigated in future research. Studies on the clinical application of H_2_S should focus more on finding appropriate treatment doses and safe, effective, and specific small molecule agonists and inhibitors.

Non-small cell lung cancer (NSCLC) accounts for 85% to 90% of all lung cancers,^386^ and is a common type of lung cancer, with lung adenocarcinoma being the most prevalent subtype within NSCLC.^387,388^ Studies have shown that disulfide death is closely related to the occurrence and progression of lung adenocarcinoma. Researchers constructed a prognostic risk score based on disulfidptosis-related genes in lung adenocarcinoma. The high-risk score group exhibited a higher mortality rate, poorer survival outcomes, and a less favorable immune microenvironment compared to the low-risk score group. This suggests that the genetic features associated with disulfidptosis have various impacts on the occurrence, proliferation, and metastasis of lung cancer, and may provide insights for identifying patient prognosis.^389^ Furthermore, the levels of SAM are elevated in patients with NSCLC, suggesting its potential as a biomarker for early-stage NSCLC.^390^ Moreover, compared to normal pulmonary epithelial cells, NSCLC cells express higher levels of CBS, CSE and 3-MST, leading to increased production of H_2_S. H_2_S can induce migration and invasion of NSCLC cells, as well as the EMT process. Additionally, H_2_S plays a significant role in the growth and angiogenesis of NSCLC by activating HIF-1α, which may provide new avenues for targeting H_2_S in therapeutic strategies.^391^ An important aspect to consider is that H_2_S may exert its effects through multiple signaling pathways, which could differ depending on the tumor type or cellular context. The interactions between H_2_S and other factors, such as ROS and NF-κB, and their collective impact on the tumor microenvironment, as well as on tumor cell proliferation, migration, and invasion, still require further in-depth exploration.

Pancreatic cancer encompasses various types, with pancreatic adenocarcinoma being the most common, typically associated with a poor prognosis and a five-year survival rate of only 5%.^392–394^ Recent findings suggest that H_2_S donor hold potential in the treatment of pancreatic cancer. Erucin (ERU) can penetrate the cell membrane of pancreatic adenocarcinoma cells and release H_2_S intracellularly. High concentrations of ERU (30-100 μmol/L) induce apoptosis by reducing the levels of phosphorylated ERK1/2 and upregulating the expression of caspase-3 and caspase-7, thereby inhibiting cancer cell proliferation. This mechanism may be related to the hyperactivation of the oncogene KRAS, leading to the excessive phosphorylation of the downstream kinase ERK1/2.^395^ Sulforaphane (SFN) also gradually releases H_2_S in the biological environment. SFN induces the production of excessive ROS, which activates the AMPK signaling pathway, promoting the translocation of Nrf2 and resulting in the inhibition of pancreatic cancer cell viability. This suggests that H_2_S plays a crucial role in suppressing the growth of pancreatic cancer cells, promoting apoptosis, and modulating the migration and invasion of pancreatic cancer cells.^396^ Moreover, demethylation treatment can disrupt protein methylation, leading to the accumulation of SAH while depleting cellular SAM, resulting in the inhibition of autophagy and apoptosis induced by endoplasmic reticulum stress in pancreatic cancer cells. Additionally, demethylation may cause an imbalance in KRAS signaling, resulting in partial inactivation of ERK and excessive activation of the PI3K/AKT-mTORC1 pathway^397^ (Fig. 3). This demethylation treatment offers a novel therapeutic strategy for patients with pancreatic adenocarcinoma. It is important to highlight that MAT, a key enzyme in the generation of SAM, also plays a significant role in pancreatic cancer. Altered expression of MAT in pancreatic cancer makes it a potential biomarker for early diagnosis and prognostic prediction. Dysregulation of MAT is associated with pathways involved in carcinogenesis, chemotherapy resistance, and the activation of tumor-associated macrophages. This suggests that direct and indirect targeting of MAT may represent a promising therapeutic strategy.^398^ Although demethylation and MAT-targeted strategies offer new perspectives for the treatment of pancreatic cancer, their clinical translation faces numerous challenges. Demethylation therapy may lead to cytotoxicity and various side effects, while optimizing the dosage and treatment duration for MAT-targeted therapy, improving drug delivery methods, and addressing potential drug resistance issues require further investigation. Currently, there is a substantial amount of preclinical research on demethylation and MAT-targeted therapies, but translating these findings into effective clinical treatment protocols requires additional clinical validation.

Ovarian cancer (OC) is a common tumor in women, often associated with poor prognosis.^399–401^ Comparative analyses between OC tissues and normal tissues led to the identification of 14 differentially expressed genes related to disulfidptosis, which were used to create a risk signature. Patients in the high-risk group exhibited lower overall survival rates. The risk assessment tool established in this study can effectively stratify risk in OC patients, facilitating personalized treatment and follow-up management for individuals affected by this disease.^402^ Sulfides play an important role in OC.^403^ CBS is a sulfur-containing amino acid metabolic enzyme highly expressed in various OC cell lines and plays a significant role in the occurrence and development of OC. Studies have shown that silencing CBS notably inhibits OC cell proliferation and metastasis, and increases sensitivity to cisplatin. Mechanistically, silencing CBS impairs H_2_S production, significantly reduces cellular GSH levels, increases ROS production, activates the tumor suppressor p53 and inhibits NF-κB activation. This indicates that downregulation of CBS can alter antioxidant levels, trigger apoptotic cascades, and enhance drug sensitivity. In cancer cells, CBS colocalizes with mitochondrial markers. And silencing CBS was found to reduce mitochondrial respiration and inhibit ATP synthesis. OC growth and drug resistance phenotype can be maintained by CBS by controlling the cellular redox response and mitochondrial bioenergetics.^404^ Furthermore, CBS drives lipid metabolism dysregulation in OC.^405^ Cells were transiently transfected with CBS siRNA to probe the transcriptional regulation of sterol regulatory element binding protein (SREBP) (lipid transcription factor). It was found that all cells showed downregulation of gene expression of SREBP1a, SREBP1c, and SREBP2, as well as their target genes such as ACC1 and FASN, which are key enzymes in lipid synthesis. Further research revealed that silencing CBS hinders Sp1 nuclear translocation, thereby affecting Sp1 binding to the SREBP-1a promoter site. The stability of the Sp1 protein is maintained through the peroxidation function in the presence of CBS. Therefore, selectively targeting CBS and modulating abnormal lipid metabolism could offer new thoughts for the therapy of ovarian cancer. However, the potential impact of CBS-targeted therapy on normal cells remains unclear. CBS is involved not only in cancer cells but also in crucial physiological processes such as sulfur metabolism and antioxidant responses. Targeting CBS may interfere with these normal functions, potentially leading to unforeseen side effects. The long-term safety, dose optimization, and management of side effects associated with CBS inhibition still require further investigation.

Prostate cancer (PC) is one of the most common cancers in men and is especially prevalent in older men.^406–408^ It was shown that CSE was upregulated in bone metastatic PC cells and in patients with advanced PC, and the high expression of CSE in patients was associated with poor survival. Further investigation revealed that CSE could promote PC cell migration and invasion, and knockdown of CSE could inhibit cell invasion by suppressing IL-1β/NF-κB-mediated signaling.^409^ SAM exhibits antitumor effects in PC cells by inducing cell cycle arrest in the S phase and inhibiting cell proliferation. Additionally, SAM increases the ratio of pro-apoptotic factor Bax to anti-apoptotic factor Bcl-2, as well as the activity of caspase-3, thereby promoting apoptosis in PC cells. The therapeutic effects of SAM are associated with the downregulation of the ERK1/2 and STAT3 signaling pathways, both of which are involved in the survival, proliferation, migration, and invasion of cancer cells.^410^ EZH2, as an oncogene, is overexpressed in PC and is associated with poor clinical outcomes in PC patients.^411,412^ Treatment of PC cells with an AHCY inhibitor leads to the accumulation of SAH and a reduction in levels of Hcy and histone H3K27 methylation, subsequently decreasing PC cell proliferation. To some extent, miR-26a induced by AHCY inhibitors can regulate the expression of EZH2,^413^ which may represent a significant mechanism of action for AHCY inhibitor therapy in prostate cancer. Although AHCY inhibitors show potential in the treatment of prostate cancer, the safety and side effects of their long-term use remain unclear. Inhibition of AHCY may interfere with normal sulfur metabolism, leading to systemic metabolic disturbances or adverse effects on other physiological processes. Furthermore, whether AHCY inhibitors could induce resistance or alter tumor cell responses to other therapies requires further investigation.

## Sulfide-based therapeutics

### H_2_S donor

The importance of maintaining endogenous H₂S homeostasis in various diseases has sparked interest in exploring pharmacological approaches to either increase or decrease its levels. Direct inhalation of H₂S gas could offer targeted therapy for pulmonary diseases; however, this approach carries risks of toxicity and flammability.^414^ Intraperitoneal or intravenous administration of inorganic H₂S donors, such as sodium sulfide and sodium hydrosulfide,^415–417^ allows for site-specific delivery. However, these compounds have a short half-life, undergo rapid oxidation, and their H₂S release is uncontrolled. Lawesson’s reagent, widely used as a sulfurizing agent in organic synthesis, can also function as an H₂S donor.^418–420^ Upon spontaneous hydrolysis in aqueous solution, it releases H₂S. However, similar to other donors, it presents the challenge of uncontrolled H₂S release.^421^ The rapid and excessive release of H₂S may exert toxic effects on the body. Therefore, the development of compounds capable of stable and controlled H₂S release has become a key focus of research.

GYY4137 is an organic H_2_S donor, chemically referred to as N-(4-hydroxyphenyl)thiourea dioxide.^182^ It is a relatively stable compound capable of releasing H_2_S in a slow and sustained manner. Compared to traditional H_2_S salts, GYY4137 has a longer half-life, allowing for a more controlled release of H_2_S over time.^422^ A ADT-OH (5-(4-hydroxyphenyl)-3H-1,2-dithiocyclopentene-3-thione) is also capable of stabilizing H_2_S levels through slow release.^423^ Studies have demonstrated that ADT-OH, as a hydrogen sulfide donor, exerts therapeutic effects in malignant melanoma^424^ and breast cancer.^425^ Phenol thiosemicarbazone (AP39) is a mitochondria-targeted H_2_S donor. AP39 maintains intracellular H_2_S homeostasis while enhancing mitochondrial antioxidant capacity, reducing inflammatory responses, and protecting cells from apoptosis.^191^ Similar to AP39, hydroxythiobenzamide (AP123) slowly releases H_2_S within the mitochondrial region of cells, thereby reducing mitochondrial oxidative stress and improving the function of the mitochondrial electron transport chain.^426,427^ ATB-346 is a compound formed by conjugating an H_2_S donor group with the non-steroidal anti-inflammatory drug (NSAID) naproxen. Unlike previous H_2_S donors, ATB-346 can only release H_2_S through metabolic processes within the body, and it is incapable of releasing H_2_S in non-biological environments. ATB-346 retains the anti-inflammatory and analgesic properties of naproxen by inhibiting cyclooxygenase (COX) enzyme activity, thereby reducing prostaglandin production and exerting its anti-inflammatory and analgesic effect.^428^ Additionally, the release of H_2_S contributes to the protection of the gastrointestinal mucosa, reducing inflammatory responses and oxidative stres.^429^ Similar to ATB-346, ATB-337 combines an H_2_S-releasing moiety with an NSAID molecule to reduce gastrointestinal side effects.^430^ In addition, garlic extracts contain H_2_S-releasing compounds, with the most characteristic being diallyl thiosulfinate. This compound rapidly decomposes in aqueous solution into various compounds, among which diallyl trisulfide (DATS) generates the highest amount of H_2_S.^431–433^ However, its limitations include a structure unsuitable for chemical modification, poor water solubility, and the potential reduction of its original physiological effects when these compounds are isolated from garlic.^432^ SG-1002 is a prodrug of H_2_S that acts solely through H_2_S signaling pathways.^434^ Its unique advantage is its oral administration, which enhances patient compliance.^435^ Multiple clinical studies have already demonstrated its potential value,^436,437^ indicating that SG-1002 warrants further investigation in the treatment of H_2_S-related diseases in the future. Notably, the strong reducing agent dithiothreitol (DTT) can react with disulfide bonds in proteins, reducing them to thiol groups.^438^ This reaction produces sulfides, which can further react to generate H_2_S. However, DTT is primarily used in biochemical experiments, particularly in the field of protein research.

Current H₂S donors show great potential, but they have limitations. Inorganic donors like sodium sulfide have rapid oxidation and uncontrolled release, while organic donors such as GYY4137 offer more stability but still face issues with dose control and toxicity. Mitochondria-targeted donors like AP39 show localized effects but lack precise control over H₂S release. Prodrugs like SG-1002 improve oral bioavailability but need further clinical validation. Compounds like ATB-346 combine H₂S release with anti-inflammatory properties but face challenges in structural optimization and solubility. Future research should focus on the development of H₂S donors that allow for more precise, controlled, and sustained release, minimizing the risks of toxicity and side effects. Efforts should also be directed toward improving the pharmacokinetic properties, such as stability and bioavailability, and investigating targeted delivery mechanisms to enhance therapeutic efficacy while reducing off-target effects.

Studies have found that several marketed drugs can upregulate intracellular H_2_S levels (Table 2). Zofenopril is an angiotensin-converting enzyme inhibitor (ACEI) that has been marketed in Europe and is widely used for the treatment of primary hypertension and acute myocardial infarction. Zofenopril alleviates vascular constriction pressure by inhibiting the generation of angiotensin II, which indirectly induces endothelial cells to release NO and H_2_S.^439–441^ N-acetylcysteine (NAC) is a widely used medication that has been FDA-approved for the treatment of acetaminophen overdose and is utilized as a mucolytic agent for respiratory conditions such as chronic obstructive pulmonary disease (COPD). The thiol structure of NAC enables it to replenish glutathione (GSH), which is crucial in antioxidant and detoxification processes. Although NAC is not a direct precursor for H_2_S generation, it indirectly upregulates H_2_S levels by modulating redox status, thereby providing protective effects against oxidative stress.^442–444^ Metformin is a biguanide antidiabetic medication that was initially approved by the FDA in 1995 for the treatment of type 2 diabetes. It primarily reduces hepatic glucose production and increases peripheral tissue sensitivity to insulin by activating the adenosine 5‘-monophosphate (AMP)-activated protein kinase (AMPK) pathway. Recent studies have demonstrated that metformin can also promote the production of H_2_S by upregulating the expression of CSE. H_2_S plays a critical role in metabolic regulation and exerts significant protective effects on the cardiovascular system and in inflammation. This mechanism may partially explain metformin’s role in preventing cardiovascular complications in diabetic patients, as well as its multiple pharmacological effects beyond metabolic improvement.^445–447^ Atorvastatin is one of the most commonly used statins, approved by the FDA in 1996 for the treatment of hypercholesterolemia and the prevention of cardiovascular events. It lowers serum low-density lipoprotein (LDL) levels not only by inhibiting HMG-CoA reductase but also by enhancing the production of H_2_S in endothelial cells, thereby exerting cardiovascular protective effects.^448–450^ Anetholedithiolethione is a sulfur-containing compound that has been shown in preclinical studies to exert protective effects on the liver and cardiovascular system by activating endogenous H_2_S production pathways. Although this drug has not been widely approved for clinical use, it is utilized in some countries as an adjunct treatment for liver diseases. It exerts antioxidant and anti-inflammatory effects through the release of H_2_S. Despite its limited clinical application, the mechanism of H_2_S release has been demonstrated in several studies.^451–453^ These medications demonstrate significant clinical efficacy within their respective therapeutic areas, and in some cases, promote the generation of H_2_S, providing a novel pharmacological mechanism for the protection of the cardiovascular system and other organs. The diversity of these drugs is reflected not only in their therapeutic uses and mechanisms of action but also in their routes of administration and widespread global production, underscoring the biological importance of H_2_S as a gaseous signaling molecule and its broad potential in pharmacological interventions. Current research focuses on the development of H_2_S donors, H_2_S prodrugs, and molecular entities capable of targeting and regulating endogenous H_2_S synthesis or metabolism. In the future, scientists will need to further investigate the safety, dose-dependence, and specific molecular mechanisms of H_2_S-related therapies to facilitate their application in clinical treatment.

**Table 2:** Summary of FDA-approved drugs that can generate H_2_S

Drug name	Indication	Date of approval	Main mechanism	Company	Dosage form	Comment
Zofenopril^439–441^	Hypertension	2000	Contains sulfhydryl groups, which can be metabolized to H_2_S. Angiotensin converting enzyme inhibitors(ACEI), which lower blood pressure by blocking angiotensin-converting enzyme activity and reducing angiotensin II production.	Menarini Group	Oral	Approved in European Union, not yet in FDA
N-acetylcysteine^442–444^	Paracetamol poisoning	1963	A precursor drug for cysteine, which produces glutathione and hydrogen sulfide. Mucolytic agent.	Cumberland Pharmaceuticals	Oral, intravenous, inhalant	
Metformin^445–447^	Diabetes	1995	Modulation of CSE expression promotes the production of H_2_S. Suppression of gluconeogenesis and enhancing insulin suppression of endogenous glucose production.	Laboratoires Aron, Bristol-Myers Squibb	Oral	
Atorvastatin^448–450^	Hyperlipidaemia, Atherosis	1996	Modulation of CSE expression promotes the production of H_2_S.	Pfizer	Oral	
Anetholedithiolethione (Sulfarlem)^451–453^	Cholecystitis, Cholelithiasis	1960s	Releaseing sulfide and promotes the production of H_2_S. Promoting bile secretion.	Laboratoires Grimerg	Oral	Approved in European countries, not yet in FDA

While several marketed drugs, including Zofenopril, NAC, Metformin, Atorvastatin, and Anetholedithiolethione, have shown the ability to upregulate endogenous H₂S production, their limitations remain. These drugs may not directly target H₂S synthesis, but rather modulate its levels indirectly, which could lead to unpredictable or suboptimal effects. The mechanisms of H_2_S release from these drugs merit study. Additionally, the specific molecular mechanisms underlying their H₂S-related actions are not fully understood, and the safety and efficacy of long-term use in relation to H₂S modulation require further investigation.

Future research should focus on developing more precise H₂S-targeted therapies, optimizing the dose-response relationship, and exploring the safety profiles of H₂S-based treatments. This includes better understanding the molecular pathways that regulate endogenous H₂S synthesis and the potential risks of chronic modulation. The development of H₂S donors, prodrugs, and molecules that can specifically target H₂S production pathways in a controlled and tissue-specific manner will be crucial for advancing clinical applications.

### Therapy targeting H_2_S-producing enzymes

**Fig. 5:**
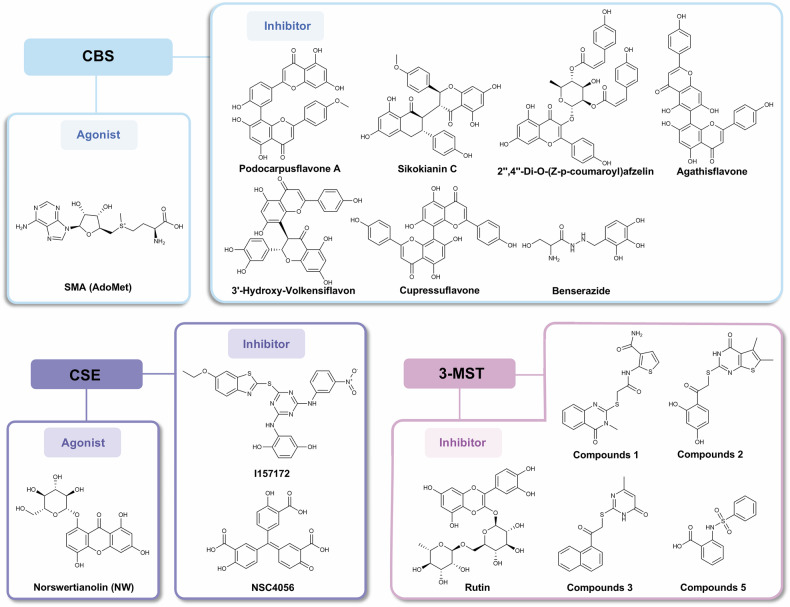
Summary of agonists and inhibitors targeting CBS, CSE and 3-MST. CBS activity can be enhanced by the agonist S-adenosylmethionine (AdoMet), while its inhibition is mediated by compounds such as Podocarpusflavone A, Sikokianin C, 2”,4”-Di-O-(Z-p-coumaroyl)afzelin, Agathisflavone, Cupressuflavone, 3’-Hydroxy-Volkensiflavon and Benserazide. CSE activity is stimulated by Norswertianolin (NW) and inhibited by I157172 and NSC4056. For 3-MST, inhibition is achieved by Rutin and Compounds 1, 2, 3, and 5

Many natural and synthetic H_2_S donors also have drawbacks such as short in vivo metabolic half-life, potential toxicity, and poor pharmacokinetics.^454–456^ Similarly, commonly used H_2_S inhibitors also lack safety and selectivity, especially in their inability to specifically inhibit CBS or CSE, causing significant challenges in the advancement of disease research.^14^ Hence, an increasing number of scientists are beginning to focus on discovering specific agonists or inhibitors of H_2_S-generating enzymes (CBS, CSE, 3-MST) based on drug screening technologies (Table 3 and Fig. 5).

**Table 3:** Summary of studies on small molecule regulators of endogenous H_2_S producing enzymes

		References	Research method	H_2_S Probe	Screened compounds	*K*_D_	IC_50_	Binding site
CBS	agonist	Kraus et al.^463^	Calorimetric methods, functional assays and kinetic modelling	\	adenosylmethionine (SAM)	*K*_D_ ~ 10 nM (a high-affinity type of sites) *K*_D_ ~ 400 nM (a lower affinity type of sites)	\	C-terminal region of CBS
	inhibitor	Szabo et al.^120^	High-throughput discovery	AzMC	benserazide	\	30 μM	CBS active site and polar residues on the periphery of the cavity such as His203, Tyr223 and Tyr308
	inhibitor	Xu et al.^467^	High-throughput discovery	CPM	six compounds fall into the category of flavonoids	\	<20 μM	\
CSE	inhibitor	Wang et al.^121^	Virtual screening	\	I157172	\	18.51 µM	\
	inhibitor	Wu et al.^469^	High-throughput discovery	DTNB	NSC4056	3.4 μM	0.6 μM	Arg and Tyr residues of CSE active site
	agonist	Geng et al.^23^	Computer molecular docking technology and microscale thermophoresis technology	\	norswertianolin (NW)	1.6 ± 0.33 μM	\	Leu68 and Asp164 of CSE
3-MST	inhibitor	Hanaoka et al.^471^	High-throughput discovery	HSip-1	compounds 1–3 and 5 all have aromatic ring-carbonyl-S -pyrimidone structure	3.0 μM (compounds 1) 0.5 μM (compounds 3)	2 ~ 7 μM	persulfurated C248 residue of 3-MST
	inhibitor	Vijayakumar et al.^474^	Molecular docking and Molecular dynamic simulation	\	quercetin 3-rutinoside (Rutin)	\	40.95 μM (promastigote) 90.09 μM (amastigote)	central active site residue CYS253 of 3-MST

*MST* 3-mercaptopyruvate sulfurtransferase, *AzMC* 7-azido-4-methylcoumarin, *CBS* cystathionine *β*-synthase, *CPM* 7-diethylamino-3-(4-maleimidophenyl)-4-methylcoumarin, *CSE* cystathionine *γ*-lyase, *DTNB* 5,5′-dithiobis (2-nitrobenzoic acid)

**Fig. 6:**
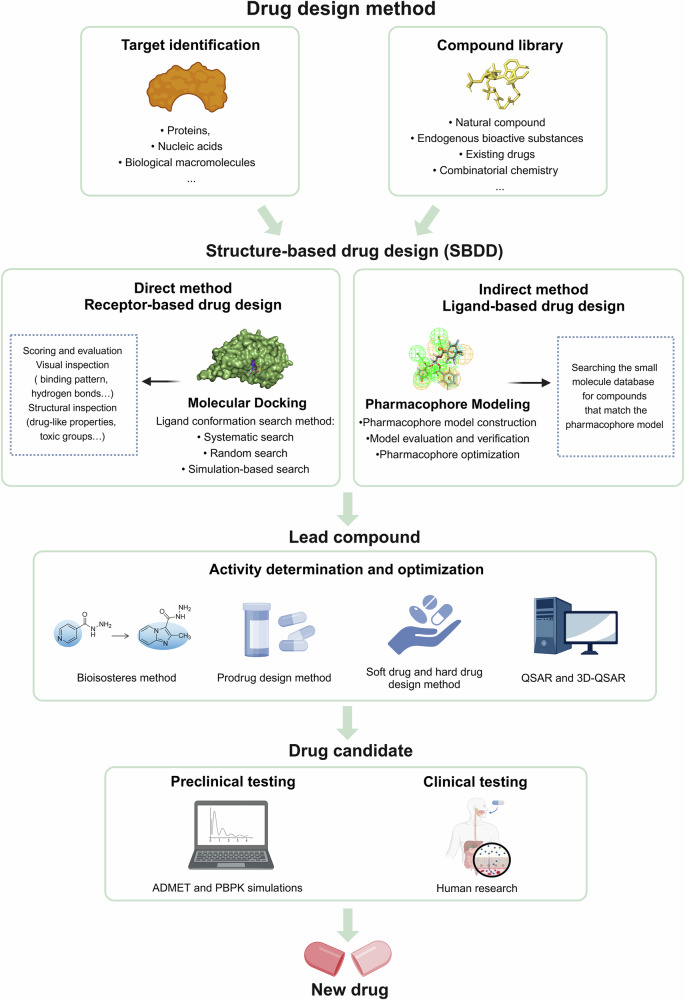
Rational drug design approaches. Drug screening begins with the identification and validation of disease targets, including proteins, nucleic acids, and other biomacromolecules. The small molecule library can be sourced from natural products, endogenous bioactive substances, existing drugs, or high-throughput screening. Structure-based drug design involves receptor-based molecular docking or ligand-based pharmacophore modeling to identify lead compounds. These leads are optimized using bioisosterism, prodrug strategies, and QSAR/3D-QSAR, followed by in silico ADMET (absorption, distribution, metabolism, excretion, toxicity) predictions and PBPK simulations for preclinical evaluation. Promising candidates are then advanced to clinical. Created with BioRender.com

Drug screening^457^ first requires identification and confirmation of disease targets. Disease targets can be discovered through disease genomics and proteomics, followed by target confirmation through reverse docking, protein structure prediction, and other methods. The small molecule library used for screening can be obtained from active components of natural products, endogenous bioactive substances, existing drugs, or through tissue chemistry and high-throughput screening. Once these two parts are prepared, structure-based drug design can be carried out, which is currently the most common screening method and is often divided into receptor-based direct methods and ligand-based indirect methods.^458,459^ The direct method involves molecular docking to predict the binding conformation of ligands and score the rationality and binding affinity of the receptor-ligand binding mode. Subsequently, visual analysis and structural inspection are conducted on the highly scored small molecules to obtain lead compounds. Indirect methods are used to obtain lead compounds by analyzing the three-dimensional structure and conformation of the active small molecule to create a pharmacophore model and subsequently searching a small molecule library for compounds that match the pharmacophore model. Lead compounds can be optimized through principles such as bioisosterism, prodrugs, quantitative structure-activity relationships (QSAR), and 3D-QSAR, followed by in silico ADMET (Absorption, Distribution, Metabolism, Excretion, Toxicity) prediction and physiologically-based pharmacokinetic simulations for preclinical testing.^460–462^ Finally, new drugs are obtained through clinical trials (Fig. 6).

#### CBS agonists

CBS is a tetrameric enzyme, with each polypeptide chain containing three domains, each of which contains different cofactors required for CBS enzyme activity.^463^ The N-terminal region binds heme, potentially playing a role in proper enzyme folding and assembly^464^; next is the central catalytic core that contains a PLP molecule forming a Schiff base with Lys119; and finally, the C-terminal regulatory domain contains a tandem of CBS domains, which bind the CBS allosteric activator SAM most likely.^463,465^ In order to study the effect of SAM on the stability and activity of wild-type CBS and mutant CBS, Krau^463^ applied a combination of calorimetric methods, functional assays, and kinetic modeling. Research has identified two distinct binding sites with different binding conformations and properties for SAM at the CBS regulatory domain. The high-affinity site binds two SAM molecules per CBS tetramer, possibly contributing to the kinetic stabilization of the regulatory domain. While the low-affinity site can accommodate up to four SAM molecules and is associated with enzyme activation. CBS half-life is nearly three times shorter under extremely low SAM concentrations, such as during methionine restriction. Under natural circumstances, SAM first stabilizes the basic conformation of CBS. With increasing concentrations of SAM, the CBS regulatory domain undergoes conformational rearrangement, leading to enzyme activation. Meanwhile, SAM maintains the activated conformation, which is essential to enzyme functio.^466^ The CBS regulatory domain can serve as a novel curative target, while SAM and its structural analogs can serve as original scaffolds for new drugs.

#### CBS inhibitors

To obtain more efficient and selective CBS inhibitors, an enzyme activity assay method compatible with high-throughput screening is needed. Szabo^120^ used a commercial AzMc probe to detect H_2_S generated by CBS and to monitor CBS activity. Researchers screened 8871 clinically used drugs and well-annotated pharmacological compounds. AOAA (a small molecule inhibitor of CBS) was used as a positive control at a compound concentration of 30 μM to assess its ability to inhibit H_2_S production. The authors identified 30 compounds for further study, among which hexachlorophene (IC_50_: 60 μM), tannic acid (IC_50_: 40 μM), and benserazide (IC_50_: 30 μM) showed concentration-dependent inhibitory effects on CBS. Additionally, benserazide could inhibit the proliferation of colon cancer cell line HT29 with high CBS expression, as well as the growth of tumors in nude mice carrying human colon cancer cell xenografts. Silico docking (Schrodinger Inc., Small-Molecule Drug Discovery Suite, 2016-1) revealed that benserazide binds to the active site of CBS (PDB ID: 1JBQ) and reacts with the PLP cofactor to form a reversible but kinetically stable Schiff base, and its trihydroxybenzyl ring can form hydrogen bonds with polar residues located in the peripheral cavity (such as His203, Tyr223, and Tyr308), resulting in strong inhibition. While benserazide shows potential as an H_2_S inhibitor, its clinical applicability remains limited by factors such as its pharmacokinetic profile and stability in vivo. The effectiveness of benserazide as a CBS inhibitor in cancer treatment requires further validation through extensive preclinical and clinical studies. Additionally, high specificity and selectivity for CBS inhibition, without off-target effects, must be ensured for therapeutic use.

Similarly, Xu^467^ also adopted a high-throughput approach to screen for CBS inhibitors. Through their research, the authors explored a new method for measuring enzyme activity that was simple, sensitive, continuous, and not interfered by enzyme assay buffer. CBS could catalyze the production of methanethiol (CH_3_SH) from methylcysteine.^468^ The enzymatic activity of CBS was then measured by reacting with the generated CH_3_SH using a commercial fluorescent thiol probe (CPM), which increased the fluorescence intensity with increasing incubation time. In order to screen for effective CBS inhibitors, the researchers selected 6491 compounds in a natural product library, including compounds isolated from plants and microorganisms, ensuring a wide range of chemical diversity and biological activity. The screening was initially performed at a concentration of 100 μM, and compounds with >80% CBS inhibition were selected for further study. A secondary screening was then conducted at a concentration of 50 μM, and 25 compounds displayed inhibition rates exceeding 50%. Half-maximal inhibitory concentration (IC_50_) values of the compounds against CBS showed that 11 compounds had IC_50_ values below 20 μM. H_2_S generation experiments were used to study the selectivity of these 11 compounds, and the results showed that two compounds exhibited similar inhibitory activities against CSE and CBS; The three compounds exhibited high selectivity for CBS, which were >10 times higher than CSE; The remaining six compounds did not inhibit the activity of CSE, even at high concentrations (IC_50_ > 400 μM). It is worth noting that these six compounds are all flavonoids, with five of them being biflavonoids. Further validation of these six compounds showed that four of them were able to inhibit the proliferation of HT29 human colon cancer cells, but the anti-proliferation mechanisms of these inhibitors still need to be further elucidated in future studies.

#### CSE agonists

Geng^23^ attempted to search for CSE activator in natural small molecules to stimulate the production and release of endogenous H_2_S. The main strategy was to use molecular docking techniques to screen natural small molecules with high affinity to CSE in the Chinese Natural Product Database (CNPD). The results showed that norswertianolin (NW) had good affinity with CSE, and microscale thermophoresis (MST)750 was used to verify the interaction between NW and CSE (*K*_D_ = 1.6 ± 0.33 μM). Meanwhile, the binding mode of the interaction between NW and CSE indicated that Leu68 and Asp164 might be important binding sites for their interaction. To verify the binding site, Niu et al. repeated the binding assay using the CSE-GFP mutant protein, and the direct binding between Leu68 mutant protein and NW was weakened, proving that Leu68 was a critical binding site for NW and CSE interaction. By detecting the effect of NW on H_2_S production in different tissue homogenates, it was found that NW increased H_2_S production in the heart, aorta, and kidney. This finding was validated in in vivo experiments, where the elevation of H_2_S in heart and kidney tissues was observed. Additionally, the researchers established an acute renal I/R injury model and found that NW significantly increased CSE activity and alleviated renal injury. In spontaneously hypertensive rats, NW up-regulated CSE expression in the aorta, increased H_2_S production, and alleviated hypertension, vascular remodeling, and inflammation. Therefore, as a newly discovered small molecule activator of CSE, NW can directly bind to CSE, thereby enhancing H_2_S production, and has potential value in the treatment of renal I/R injury and cardiovascular diseases.

#### CSE inhibitors

Inspired by the high-throughput screening method for CBS inhibitors, scientists attempted to use this method to screen for CSE inhibitors. In order to monitor the activity of CSE in a high-throughput mode, Wu et al. designed a method based on tandem-well plate to measure the production of H_2_S.^469^ The designed 192-tandem-well plate was modified from a traditional 384-well assay plate by connecting the upper channels between adjacent wells for gas exchange. This device traps and accurately measures the gas generated in the enzymatic reaction solution well and the well adjacent to it, thus eliminating interference from the assay reagents on the enzyme and its substrate. Through this high-throughput screening method,^470^ 62 compounds with CSE inhibition rates >50% were initially selected from 11,954 compounds, with 10 compounds having IC_50_ values <10 μM. The most potent inhibitor was NSC4056, with an IC_50_ value of 0.6 μM. Subsequently, researchers tested the effect of NSC4056 on CBS activity, and the results displayed that NSC4056 had a higher inhibitory activity on CSE compared to CBS, with a selectivity of 137-fold higher than CBS. Counter screen assays confirmed the inhibitory activity of NSC4056 on CSE enzymes, indicating that it was not simply trapping H_2_S. It was also discovered that the inhibitory effect of NSC4056 on CSE could be reversed, suggesting that this compound may be a noncovalent inhibitor of CSE. Unlike most current CSE inhibitors, NSC4056 exerts competitive inhibition on the substrate L-cysteine, rather than being a competitive inhibitor of PLP. NSC4056 completely occupies the same binding site as the L-cysteine substrate, with its two carboxyl groups forming six hydrogen bonds with Arg62, Tyr114, and Arg119, collectively ensuring the inhibitory effect. Structure-activity relationship (SAR)^470^ is an important method for guiding the optimization design of lead compounds and its study results have shown that the carboxyl group and tripod structure of NSC4056 are essential for inhibiting CSE activity. Further investigation into the effects of NSC4056 on cells revealed that it could reduce the endogenous H_2_S levels in Raw264.7 cells, with an IC_50_ value of 43.2 μM, effectively alleviating hypotension in hemorrhagic shock rats. The tandem-well analysis method provides a foundation for the development of more effective and selective CSE inhibitors, and the development of new inhibitors offers new insights into the treatment of related diseases.

Wang^121^ studied CSE inhibitors using virtual screening technology. The Site Finder module was used to identify the binding pocket of the CSE protein (PDB ID: 3COG), and the Wash module was used to prepare the SPECS compound library (200,000 compounds) to investigate the affinity of compounds for the CSE protein. MOE Dock was used for docking simulations of the ligands. Initially, high-throughput rigid docking was used to filter out many inactive small molecules. Subsequently, the selected 20,000 compounds were subjected to flexible docking using force field refinement to obtain 1000 compounds. These compounds were then ranked, and the top 100 compounds were selected. Among them, I157172 (S: -7.9215) exhibited the highest binding affinity to the protein. To confirm the inhibitory activity of I157172 on CSE, the study further examined the effects of I157172 on H_2_S production and CSE protein expression. Western blot results also demonstrated that I157172 significantly inhibited CSE protein expression in MCF7 cells. Therefore, I157172 efficiently suppressed the proliferation, migration, and invasion of MCF7 cells. It could be a potential candidate drug for the treatment of breast cancer and further exploration needed to understand its anticancer mechanisms in vivo. It is important to note that the virtual screening approach may not fully account for the dynamic nature of protein-ligand interactions under physiological conditions, which could lead to discrepancies between in silico predictions and actual biological activity. Furthermore, although I157172 demonstrated inhibitory effects on CSE expression in vitro, its efficacy and safety in vivo have not been fully validated, necessitating further preclinical and clinical studies.

#### 3-MST inhibitors

Hanaok^471^ used H_2_S selective fluorescent probe HSip-1 for high-throughput screening of a large compound library and discovered effective 3-MST inhibitors. 3-MST utilizes 3-MP as a substrate to generate H_2_S through enzymatic reaction. HSip-1 is then added to the solution as a fluorescent probe to monitor the production of H_2_S and evaluate the compound’s activity. The researchers screened a library containing 174,118 compounds, testing all compounds at 10 μM concentration. False positives were eliminated by combining inhibitory activity, resulting in 146 compounds. The dose-dependency (0.25, 1, 3, 10 and 30 μM) of 3-MST-inhibitory activity of each compound was examined to determine the IC_50_. Compounds 1–3, 5 showed >80% inhibition of 3-MST activity at 10 μM, and their IC_50_ values were 2–7 μM. High-level coupled-cluster calculation (CCSD(T)) combined with a large aug-cc-pVDZ basis set revealed that compounds 1-3 shared a similar structural backbone, characterized by an aromatic ring-carbonyl-S-pyrimidinone structure. Subsequently, the selectivity of these compounds to 3-MST was examined. Gas chromatography was used to determine the selectivity of compounds 1-3 and 5 towards two other enzymes, CSE and CBS, which also catalyze the production of H_2_S. It was found that compound 3 exhibited almost no activity against CBS and CSE, indicating its highest selectivity to 3-MST. The X-ray crystal structures of the 3-MST complexes with 1 and 3 revealed that their active pockets were located at the active site of 3-MST, specifically at the perthiolated cysteine residue. Persulfur anions of persulfurated cysteine residues and positively charged carbonyl carbons of pyrimidone moiety of inhibitor have a substantial long-range electrostatic interaction, according to theoretical calculations. The pKa value determined for the cysteine persulfide is 4.34, suggesting that the persulfurated cysteine residue is present in a negatively charged deprotonated state, aligning with the robust electrostatic interaction observed in this investigation. Currently, 3-MST knockout mice have been utilized in many disease studies,^472^ and these mice are also important tools for investigating the physiological functions of 3-MST. In future work, extensive in vitro and in vivo studies can be conducted with the identified selective 3-MST inhibitors, providing a basis for the treatment of cancer, cardiovascular, and neurological diseases.^131^

In addition to focusing on mammals, 3-MS^473^ also has an influence in the generation of sulfides in Leishmania. Vijayakumar^474^ used molecular docking (the Genetic Optimization for Ligand Docking (GOLD) software) and molecular dynamic simulations to screen the binding affinity of natural compounds against L. donovani 3-MST (Ld3MST). Compared to mammalian 3-MST, Leishmania 3-MST contains an additional 70 amino acids in the C-terminal domain. Due to the lack of X-ray and nuclear magnetic resonance structures for Ld3MST, homology model is required. Using homology model, the Ld3MST protein was docked with 5,284 natural compounds, and the active site and affinity of 3-MP binding to Ld3MST were used as positive controls. A total of 275 natural compounds were discovered. Among them, the dock score (55.30) and binding energy score (-10.5 kcal/mol) of quercetin 3-rutinoside (Rutin) were higher than 3-MP, and Rutin forms more hydrogen bonds with the active site than other compounds. Subsequently, molecular dynamics simulations were performed on three systems: Ld3MST, Ld3MST-3-MP, and Ld3MST-Rutin complex. The trajectories of each system were analyzed and compared. It was found that the average RMSD value of the Ld3MST-Rutin complex (~0.35 nm) was lower than that of other systems, indicating better stability of the Ld3MST-Rutin complex. At the same time, the radius of gyration (Rg) of the Ld3MST-Rutin complex converged over time, and the solvent accessible surface area (SASA, ~44 nm^2^) remained stable, indicating a compactness and a more stable binding of the complex. Considering the high number of hydrogen bonds and strong binding affinity between Ld3MST and Rutin, Rutin can be considered a potential inhibitor of Ld3MST. Furthermore, the activity of Rutin was verified, with IC_50_ values of 40.95 μM and 90.09 μM for promastigotes and amastigotes, respectively, and no cytotoxicity at a concentration of 819.00 μM. This study suggests that Rutin may be an effective compound that can be used in combination with other anti-leishmanial drugs. This study also highlights the use of molecular docking and molecular dynamics simulations for screening selective inhibitors of human 3-MST.

Unfortunately, current specific small molecule screening targeting DAO is mainly focused on the effect of DAO on the N-methyl-D-aspartate (NMDA) receptor,^475–477^ while neglecting the important role of DAO in generating H_2_S, which is also a new direction for future exploration of H_2_S targeted therapy.

### Clinical trials of H_2_S

Research has found that patients with heart failure (HF) have lower circulating levels of H_2_S, and there is a negative correlation between H_2_S levels and the severity of HF.^478^ A novel H_2_S prodrug, SG1002, can increase plasma H_2_S levels (Table 4). The prevalence and incidence of heart failure with preserved ejection fraction (HFpEF) have been rising, with nearly half of all heart failure patients globally being classified as this subtype.^479^ A preclinical study indicated that,^480^ adjunctive treatment with SG1002 can enhance circulating and tissue levels of H_2_S, alleviating cardiac dysfunction in HFpEF and significantly reducing cardiac interstitial fibrosis. Additionally, Krum^436^ found that H_2_S levels were lower in patients with HF and were negatively correlated with the severity of HF. A novel H_2_S precursor, SG1002, was shown to increase plasma H_2_S levels. Therefore, Krum conducted a Phase I clinical trial to evaluate the changes in H_2_S levels, treatment effectiveness, and safety of oral SG1002 in both healthy individuals and HF patients. The results showed that SG1002 reduced the levels of B-type natriuretic peptide (BNP), a biomarker for cardiac injury, in the patients’ blood and demonstrated a stable drug level throughout the trial. Furthermore, SG1002 was safe and well tolerated in all participants at all doses. These data suggest that SG1002 is a promising new drug for treating heart failure, but further investigation is needed in larger-scale clinical studies to determine its precise role. The Phase I trial of SG1002 primarily focused on safety and H_2_S levels, without fully addressing long-term efficacy and potential side effects. While the drug showed promise in reducing BNP levels, its impact on overall heart failure progression and survival remains unclear. Additionally, larger, more diverse clinical trials are needed to confirm the drug’s effectiveness across different stages of heart failure and in various patient populations.

**Table 4:** Preclinical and clinical studies based on H_2_S therapy

Institution	Clinical indications	Lead drug	Comment	Stage of development	Clinical trial registration number
Cedars-Sinai Medical Center	Heart failure with preserved ejection fraction	SG1002	Polyvalent sulfur	Preclinical^480^	
Monash University	Heart failure			Phase I^436^	NCT01989208
University Medical Center Groningen	Acute coronary syndrome	Na_2_S_2_O_3_	Inorganic salt	Phase I^483^	
University of Texas Medical Branch	Inflammation, oxidative stress	AP39	Mitochondrion-targeted H_2_S release	Preclinical^484^	
Antibe Therapeutics	Acute pain	ATB-352	Ketoprofen derivative	Preclinical^490^	
	Pain, inflammation	ATB-346	Naproxen derivative	Phase II^492^	NCT03291418
City University of New York	Inflammation Cancer	NBS-1120	Aspirin derivative	Preclinical^497^	
Gicare Pharma Inc	Colonic pain	GIC-1001	Trimebutine salt	Phase I^499^	NCT01738425
				Phase IIa	NCT01926444

Na_2_S_2_O_3_, a clinically approved H_2_S donor with little side effects, used for the treatment of cyanide poisoning,^481,482^ calcific uremic arteriolopathy, and chemotherapy-induced nephrotoxicity (Table 4). A study was conducted on 18 patients who underwent coronary angiography due to acute coronary syndrome. The patients were intravenously administered Na_2_S_2_O_3_ in combination with vasodilators and antihypertensive drugs. No severe adverse events were observed, with only two patients experiencing transient hypotension and one patient experiencing mild nausea. This indicates the safety and tolerability of Na_2_S_2_O_3_ in patients with acute coronary syndrome. However, this study has limitations like a small sample size and a short-term following up, and the benefits of Na_2_S_2_O_3_ treatment must be further tested in larger studies.^483^ The study on Na₂S₂O₃ demonstrated its safety and tolerability in acute coronary syndrome patients, but its small sample size limits the generalizability of the results. The short-term follow-up period also restricts the understanding of long-term efficacy and potential side effects. Larger, more comprehensive studies with extended follow-up are necessary to fully evaluate the therapeutic benefits and risks of Na₂S₂O₃ in coronary artery disease and other cardiovascular conditions.

As previously mentioned, AP39 is a mitochondria-targeted H_2_S donor (Table 4). A preclinical study has investigated the effects of AP39 on endothelial cells under both baseline and oxidative stress conditions.^484^ AP39 has been shown to increase H_2_S levels within the mitochondria of endothelial cells. Furthermore, the effects of AP39 on mitochondrial activity are concentration-dependent; at low concentrations (30-100 nM), it stimulates mitochondrial electron transport and cellular bioenergetics, while at higher concentrations (300 nM), it exerts an inhibitory effect on mitochondria. Under oxidative stress conditions, pre-treatment with AP39 can attenuate its impact on mitochondrial activity and prevent mitochondrial DNA damage. Mitochondrial dysfunction plays a critical role in cardiovascular diseases, inflammatory disorders, and various critical illnesses, often accompanied by a disruption in the body’s H_2_S balance.^485–487^ Therefore, mitochondria-selective H_2_S donors may offer potential therapeutic benefits under certain pathophysiological conditions.

The covalent linkage of NSAIDs with H_2_S releasing moieties has been shown to significantly reduce gastrointestinal damage and bleeding while enhancing anti-inflammatory and analgesic efficacy.^488,489^ ATB-352 is a novel H_2_S-releasing anti-inflammatory agent (Table 4). A preclinical study has evaluated the potential of ATB-352 for application in clinical research.^490^ In a mouse model of nociceptive hypersensitivity, ATB-352 demonstrated greater analgesic efficacy compared to ketorolac. Ketorolac is a highly effective NSAID but is associated with significant gastrointestinal toxicity. The analgesic effect of 30 mg/kg of ketorolac is comparable to that of ATB-352 at one-third of the molar equivalent dose. Although ATB-352 exhibits notable inhibitory effects on COX, it does not induce gastrointestinal damage. Anandamide is an endogenous cannabinoid that exerts analgesic effects by activating CB1 receptors, and it is considered an analgesic mediator.^491^ Pre-treatment with the CB1 antagonist AM251 can reverse the analgesic effects of ATB-352, but it does not diminish the analgesic effects of ketorolac. This indicates that endogenous cannabinoids play a significant role in the analgesic properties of ATB-352. Additionally, it is noteworthy that ATB-352 does not activate μ-opioid receptors even at concentrations as high as 30 μg/mL. Therefore, this compound may serve as a promising alternative to opioid analgesics in the treatment of severe pain, potentially addressing the opioid crisis. ATB-352 shows promising analgesic effects with less gastrointestinal toxicity compared to ketorolac, but its long-term safety and human applicability require further clinical evaluation. The exact mechanisms, particularly the role of cannabinoid signaling, need more exploration. While it may serve as an opioid alternative, more comprehensive trials are needed to confirm its efficacy and safety.

ATB-346 is an H_2_S-NSAID derived from naproxen (Table 4). NSAIDs exert their effects primarily by inhibiting COX, but they often lead to gastrointestinal adverse reactions. In a Phase II clinical trial conducted by researchers,^492^ 244 healthy volunteers were divided into two groups and received either ATB-346 or naproxen, with upper gastrointestinal ulcers assessed through endoscopic examination. Both drugs showed similar inhibition efficiency on COX activity, but the naproxen group experienced more ulcers, particularly larger ones, compared to the ATB-346 group. The incidence of dyspepsia, abdominal pain, and gastroesophageal reflux was lower in the ATB-346 group than in the naproxen group. Additionally, the plasma H_2_S levels in the ATB-346 group were significantly higher than in the naproxen group, suggesting that H_2_S may play a role in reducing gastrointestinal toxicity. Gilroy^493^ also conducted a study on ATB-346. They created an acute skin inflammation model in 23 healthy volunteers and measured the accumulation of inflammatory cells and factors within the inflamed skin. ATB-346 was found to have a strong anti-inflammatory effect, suggesting that H_2_S release is involved in the regulation of inflammation. However, similar to existing clinical studies, this study had a limited number of participants and lacked subsequent investigations. Nevertheless, it has provided initial evidence of the tremendous potential of H_2_S in clinical therapy, and we look forward to more follow-up clinical research on H_2_S in the future.

Furthermore, it is important to mention that NO-releasing NSAIDs have been shown to be effective in preclinical models of inflammation with fewer side effects.^494,495^ Consequently, researchers have developed a novel compound that combines both NO and H_2_S gas signaling molecules with the aspirin molecule, referred to as NOSH-aspirin (NBS-1120)^496^ (Table 4). A preclinical study investigated the gastrointestinal safety, anti-inflammatory, analgesic, antipyretic, antiplatelet, and tumor-inhibiting effects of equimolar doses of aspirin and NBS-1120 in rats.^497^ The findings indicated that NBS-1120 exhibited lower toxicity and superior safety compared to aspirin. Furthermore, NBS-1120 demonstrated a greater efficacy as a chemopreventive agent, with its effects on tumor growth inhibition and tumor size reduction being dose-dependent. At the highest dose of 100 mg/kg (0.21 mmol/kg), the tumor growth reduction rate was 95%, and the tumor mass reduction rate was 97%. Although NBS-1120 demonstrates superior safety and efficacy compared to aspirin, its potential interactions with other drugs or underlying conditions have not been fully explored. The tumor inhibition results, while promising, are based on preclinical models, and their translation to clinical settings remains uncertain. Furthermore, the long-term pharmacokinetics and toxicity of NBS-1120 need more rigorous investigation before widespread clinical use.

In addition to combining with NSAIDs, researchers have identified that conjugating the H₂S-releasing counterion 3-thiocarbamoylbenzoate (3TCB) with trimebutine can enhance its antispasmodic effect in the gastrointestinal tract. At equimolar doses, this novel compound, GIC-1001 (trimebutine 3-thiocarbamoylbenzene-sulphonate), shows rapid onset of action (2 h) and more effectively mitigates nociceptive responses to colorectal distension, providing pain relief during colonoscopy (Table 4). Additionally, GIC-1001 exhibits significant physical stability and exerts an antihyperalgesic effect through H₂S release.^498^ As a result, GIC-1001 has been selected as a drug development candidate and has progressed to clinical investigation. In 2012, a Phase I randomized, double-blind, placebo-controlled, comprehensive study was conducted in Canada to evaluate the safety, tolerability, and pharmacokinetics of single and multiple escalating oral doses of GIC-1001, as well as the impact of food on the pharmacokinetics of a single oral dose of GIC-1001 in healthy subjects.^499^ The results showed no adverse events related to vital signs or ECGs with either single or multiple escalating doses. The pharmacokinetics of GIC-1001 were primarily linear and dose-proportional. These findings indicate that GIC-1001 is well-tolerated, with a safety profile comparable to placebo, and that its safety is consistent under both fed and fasted conditions. However, variability in food intake posed challenges for pharmacokinetic analysis, making it difficult to determine whether GIC-1001 should be taken on an empty stomach. Given that GIC-1001 is intended for use in colonoscopy, where patients must fast beforehand, it is recommended that the drug be administered in a fasted state. Subsequently, a Phase IIa study was conducted in 2013 (ClinicalTrials.gov ID: NCT01926444) involving 240 patients. Eligible patients were randomized in a 1:1:1:2 ratio into one of four treatment groups: GIC-1001 at doses of 250 mg, 375 mg, or 500 mg, and a matching placebo group. Patients underwent unsedated colonoscopy within 3 days of taking the assigned medication. Results showed that the moderate dose (375 mg) of GIC-1001 reduced pain by 26.6% compared to placebo. Based on these findings, GIC-1001 demonstrates promise as a novel colonic analgesic, warranting further clinical investigation.

## Conclusion and perspective

In recent decades, the presence of sulfur-containing amino acid metabolic pathway in various cells of the human body has been recognized, with their metabolic products serving as endogenous bioactive molecules responsible for maintaining health. These molecules play crucial roles in multiple biological processes, including anti-inflammatory responses, methylation, antioxidant defense, membrane stabilization, ion transport, osmoregulation, protein synthesis, and energy metabolism. Dysregulation of these sulfur-containing biomolecules can lead to numerous pathological consequences across different systems. By exploring the metabolic mechanisms, signaling pathways, and potential impacts of these sulfur-containing biomolecules in various diseases, we lay the groundwork for understanding their complex biological functions and clinical applications. Importantly, Hcy has been widely recognized as an independent risk factor for cardiovascular diseases. This suggests that future interventions targeting Hcy metabolism may help prevent related diseases. Research on SAM has also provided new perspectives for the development of antidepressant and neuroprotective drugs.^500,501^ SAM plays a crucial role in maintaining brain function and offers significant insights for the treatment of mood regulation, neuroinflammation, and neurodegenerative diseases.^502,503^ Additionally, cystine, with its antioxidant properties and role in H_2_S production, positions it as a potential intervention target for combating oxidative stress, inflammation, and metabolic disorders. H_2_S, as an endogenous gaseous signaling molecule, participates in life activities such as cell survival, proliferation, differentiation, migration, and death by regulating energy metabolism, inflammatory response, and oxidative homeostatic balance, and plays an important biological effect in physiological and pathophysiological regulation of multiple systems in the body. Currently, there is a large controversy in H_2_S research. H_2_S is a double-edged sword that tends to have opposite effects on certain biological processes. For example, the pro-inflammatory and anti-inflammatory effects of H_2_S. H_2_S activates NF-κB in pancreatic alveolar cells, promoting inflammation, but inhibit NF-κB in HUVECs, suppressing inflammation. H_2_S also has a dual role in tumors. H_2_S can inhibit the aberrant activation of the β-catenin pathway, which in turn inhibits TNBC cell viability, as well as enhance mitochondrial function and glycolysis which in turn promotes colon cancer growth. However, this also indicates that H_2_S possesses significant research potential. Clarifying the mechanisms by which H_2_S functions in specific diseases and identifying targeted therapeutic agents could lay the foundation for future treatment strategies.

In future research exploration, the following aspects are worth considering: (1) The role of the methionine cycle in disease prevention and personalized treatment is increasingly recognized. By integrating metabolic biomarkers with genomic and metabolomic data, personalized intervention strategies can be developed, particularly for patients with metabolic disorders. However, the interactions among sulfur-containing biomolecules and their relationships with other nutrients or pharmaceuticals remain incompletely understood, limiting research on their potential synergistic effects. Therefore, future studies should further explore these interactions and expand research on the physiological roles of sulfur-containing biomolecules in immune function and other underexplored systems, especially their role in modulating immune responses and the reactivation of HIV latency.^504^ (2) Current studies on sulfur-containing biomolecules mostly focus on rats and mice, lacking clinical evidence. The effects of H_2_S could be studied in large animal models, such as pigs and monkeys, which exhibit similar characteristics to human diseases, to provide a research foundation for clinical trials. (3) In clinical practice, the coexistence of multiple chronic diseases often results in suboptimal treatment outcomes. Therefore, research should expand to explore the therapeutic effects of sulfur-containing biomolecules in the context of comorbidities, leveraging their multi-systemic functions to develop more effective integrated treatment strategies. Additionally, since the effects of sulfur-containing biomolecules are highly tissue-specific, clinical studies should focus on designing methods to selectively modulate the levels of these molecules in specific tissues, ensuring targeted local effects while minimizing potential adverse outcomes in other tissues. (4) Strengthen research on DAO, as current studies mainly focus on the three H_2_S-producing enzymes CBS, CSE, and 3-MST. In fact, DAO plays a major part in the generation of H_2_S in the cerebellum and kidneys. Exploring new mechanisms of H_2_S through DAO as a target can provide new insights for the clinical treatment of cerebellum and kidney-related diseases. (5) When studying endogenous sulfur-containing biomolecules, pay attention to their interaction with other endogenous bioactive small molecules, especially as the fourth emerging endogenous gaseous signal molecule SO_2_. H_2_S can oxidize to produce SO_2_, and both are generated from L-Cys. Therefore, it is important to consider the sulfur-containing amino acid metabolic family as a complex network. Reversing the disrupted sulfur-containing amino acid metabolic homeostasis in diseases is an important scientific question for future research.^505^

## References

[1] Gamgee, A. & Dewar, J. Researches on the constitution and physiological relations of cystine (C(3)H(5)NO(2)S). *J. Anat. Physiol.* **5**, 142–149 (1870). 17230873 PMC1318819

[2] Plimmer, R. H. The separation of cystine and tyrosine. *Biochem J.* **7**, 311–317 (1913). 16742251 10.1042/bj0070311 PMC1276474

[3] Go, Y. M. & Jones, D. P. Cysteine/cystine redox signaling in cardiovascular disease. *Free Radic. Biol. Med.* **50**, 495–509 (2011). 21130865 10.1016/j.freeradbiomed.2010.11.029 PMC3040416

[4] Madden, S. C. et al. Ten amino acids essential for plasma protein production effective orally or intravenously. *J. Exp. Med.* **77**, 277–295 (1943). 19871282 10.1084/jem.77.3.277 PMC2135333

[5] Madden, S. C., Woods, R. R., Shull, F. W. & Whipple, G. H. Amino acid mixtures effective parenterally for long continued plasma protein production. Casein digests compared. *J. Exp. Med.* **79**, 607–624 (1944). 19871390 10.1084/jem.79.6.607 PMC2135383

[6] Bruk, G. et al. Hyperhomocysteinemia as a cause of erectile dysfunction. *Georgian Med. News* **350**, 54–56 (2024). 39089271

[7] Du Vigneaud, V., Loring, H. S. & Craft, H. A. The oxidation of the sulfur of homocystine, methionine, and S-METHYLCYSTEINE in the animal body. *J. Biol. Chem.* **105**, 481–488 (1934).

[8] Szabo, C. A timeline of hydrogen sulfide (H(2)S) research: from environmental toxin to biological mediator. *Biochem Pharm.* **149**, 5–19 (2018). 28947277 10.1016/j.bcp.2017.09.010 PMC5862769

[9] Cantoni, G. L. S-Adenosylmethionine; a new intermediate formed enzymatically from L-methionine and adenosinetriphosphate. *J. Biol. Chem.* **204**, 403–416 (1953). 13084611

[10] De La Haba, G. & Cantoni, G. L. The enzymatic synthesis of S-adenosyl-L-homocysteine from adenosine and homocysteine. *J. Biol. Chem.* **234**, 603–608 (1959). 13641268

[11] Abe, K. & Kimura, H. The possible role of hydrogen sulfide as an endogenous neuromodulator. *J. Neurosci.* **16**, 1066–1071 (1996). 8558235 10.1523/JNEUROSCI.16-03-01066.1996 PMC6578817

[12] Hosoki, R., Matsuki, N. & Kimura, H. The possible role of hydrogen sulfide as an endogenous smooth muscle relaxant in synergy with nitric oxide. *Biochem Biophys. Res. Commun.* **237**, 527–531 (1997). 9299397 10.1006/bbrc.1997.6878

[13] Li, J. et al. Hydrogen sulfide attenuates ferroptosis and stimulates autophagy by blocking mTOR signaling in sepsis-induced acute lung injury. *Mol. Immunol.* **141**, 318–327 (2022). 34952420 10.1016/j.molimm.2021.12.003

[14] Shatalin, K. et al. Inhibitors of bacterial H(2)S biogenesis targeting antibiotic resistance and tolerance. *Science* **372**, 1169–1175 (2021). 34112687 10.1126/science.abd8377 PMC10723041

[15] Saaranen, M. J. & Ruddock, L. W. Applications of catalyzed cytoplasmic disulfide bond formation. *Biochem. Soc. Trans.* **47**, 1223–1231 (2019). 31671179 10.1042/BST20190088

[16] Lu, S. C. Glutathione synthesis. *Biochim. Biophys. Acta* **1830**, 3143–3153 (2013). 22995213 10.1016/j.bbagen.2012.09.008 PMC3549305

[17] Guertin, D. A. & Wellen, K. E. Acetyl-CoA metabolism in cancer. *Nat. Rev. Cancer* **23**, 156–172 (2023). 36658431 10.1038/s41568-022-00543-5 PMC11137663

[18] Jomova, K. & Valko, M. Advances in metal-induced oxidative stress and human disease. *Toxicology* **283**, 65–87 (2011). 21414382 10.1016/j.tox.2011.03.001

[19] Fang, W. et al. Methionine restriction constrains lipoylation and activates mitochondria for nitrogenic synthesis of amino acids. *Nat. Commun.* **14**, 2504 (2023). 37130856 10.1038/s41467-023-38289-9 PMC10154411

[20] Wu, G. et al. Dietary methionine restriction ameliorated fat accumulation, systemic inflammation, and increased energy metabolism by altering gut microbiota in middle-aged mice administered different fat diets. *J. Agric. Food Chem.* **68**, 7745–7756 (2020). 32597175 10.1021/acs.jafc.0c02965

[21] Djuric, D. M. Editorial: sulfur-containing amino acids in cardiovascular and neural physiology, pathophysiology and pharmacology: an overview and update. *Curr. Med. Chem.* **25**, 322–323 (2018). 29431604 10.2174/092986732503180130142900

[22] Zhang, P. et al. Role of hydrogen sulfide in myocardial ischemia-reperfusion injury. *J. Cardiovasc. Pharm.* **77**, 130–141 (2021). 10.1097/FJC.0000000000000943 33165141

[23] Niu, Y. et al. Norswertianolin promotes cystathionine gamma-lyase activity and attenuates renal ischemia/reperfusion injury and hypertension. *Front Pharm.* **12**, 677212 (2021). 10.3389/fphar.2021.677212 PMC8317460 34335249

[24] Loiselle, J. J., Yang, G. & Wu, L. Hydrogen sulfide and hepatic lipid metabolism - a critical pairing for liver health. *Br. J. Pharm.* **177**, 757–768 (2020). 10.1111/bph.14556 PMC7024709 30499137

[25] Huang, Y. et al. Hydrogen sulfide and its donors for the treatment of cerebral ischaemia-reperfusion injury: a comprehensive review. *Biomed. Pharmacother.* **161**, 114506 (2023). 36906977 10.1016/j.biopha.2023.114506

[26] Sen, S. et al. Role of cystathionine beta-synthase in human breast cancer. *Free Radic. Biol. Med.* **86**, 228–238 (2015). 26051168 10.1016/j.freeradbiomed.2015.05.024

[27] Phillips, C. M. et al. Upregulation of cystathionine-beta-synthase in colonic epithelia reprograms metabolism and promotes carcinogenesis. *Cancer Res.* **77**, 5741–5754 (2017). 28923859 10.1158/0008-5472.CAN-16-3480 PMC5668191

[28] Guo, H. et al. Characterization of hydrogen sulfide and its synthases, cystathionine beta-synthase and cystathionine gamma-lyase, in human prostatic tissue and cells. *Urology* **79**, 483 e481–483.e485 (2012). 10.1016/j.urology.2011.10.013 22310774

[29] Guo, S. et al. The CBS-H(2)S axis promotes liver metastasis of colon cancer by upregulating VEGF through AP-1 activation. *Br. J. Cancer* **126**, 1055–1066 (2022). 34952931 10.1038/s41416-021-01681-7 PMC8979992

[30] Shackelford, R. E. et al. Molecular functions of hydrogen sulfide in cancer. *Pathophysiology* **28**, 437–456 (2021). 35366284 10.3390/pathophysiology28030028 PMC8830448

[31] You, J. et al. Cystathionine- gamma-lyase promotes process of breast cancer in association with STAT3 signaling pathway. *Oncotarget* **8**, 65677–65686 (2017). 29029463 10.18632/oncotarget.20057 PMC5630363

[32] Paulusma, C. C., Lamers, W. H., Broer, S. & van de Graaf, S. F. J. Amino acid metabolism, transport and signalling in the liver revisited. *Biochem Pharm.* **201**, 115074 (2022). 35568239 10.1016/j.bcp.2022.115074

[33] Li, C. et al. Amino acid catabolism regulates hematopoietic stem cell proteostasis via a GCN2-eIF2alpha axis. *Cell Stem Cell* **29**, 1119–1134.e1117 (2022). 35803229 10.1016/j.stem.2022.06.004

[34] Li, Z. & Zhang, H. Reprogramming of glucose, fatty acid and amino acid metabolism for cancer progression. *Cell Mol. Life Sci.* **73**, 377–392 (2016). 26499846 10.1007/s00018-015-2070-4 PMC11108301

[35] Pencharz, P. B. & Ball, R. O. Amino acid needs for early growth and development. *J. Nutr.* **134**, 1566S–1568S (2004). 15173431 10.1093/jn/134.6.1566S

[36] Terstappen, F. et al. Prenatal amino acid supplementation to improve fetal growth: a systematic review and meta-analysis. *Nutrients*. **12**, 2535 (2020). 10.3390/nu12092535 PMC7551332 32825593

[37] Balakrishnan, M. et al. Growth and neurodevelopmental outcomes of early, high-dose parenteral amino acid intake in very low birth weight infants: a randomized controlled trial. *JPEN J. Parenter. Enter. Nutr.* **42**, 597–606 (2018). 10.1177/0148607117696330 29187120

[38] Li, P. et al. Amino acids and immune function. *Br. J. Nutr.* **98**, 237–252 (2007). 17403271 10.1017/S000711450769936X

[39] Han, C., Ge, M., Ho, P. C. & Zhang, L. Fueling T-cell antitumor immunity: amino acid metabolism revisited. *Cancer Immunol. Res.* **9**, 1373–1382 (2021). 34716193 10.1158/2326-6066.CIR-21-0459

[40] McGaha, T. L. et al. Amino acid catabolism: a pivotal regulator of innate and adaptive immunity. *Immunol. Rev.* **249**, 135–157 (2012). 22889220 10.1111/j.1600-065X.2012.01149.x PMC4384693

[41] Brosnan, J. T. & Brosnan, M. E. The sulfur-containing amino acids: an overview. *J. Nutr.* **136**, 1636S–1640S (2006). 16702333 10.1093/jn/136.6.1636S

[42] Willke, T. Methionine production–a critical review. *Appl Microbiol. Biotechnol.* **98**, 9893–9914 (2014). 25381187 10.1007/s00253-014-6156-y

[43] Sanderson, S. M., Gao, X., Dai, Z. & Locasale, J. W. Methionine metabolism in health and cancer: a nexus of diet and precision medicine. *Nat. Rev. Cancer* **19**, 625–637 (2019). 31515518 10.1038/s41568-019-0187-8

[44] Ouyang, Y. et al. S-adenosylmethionine: A metabolite critical to the regulation of autophagy. *Cell Prolif.* **53**, e12891 (2020). 33030764 10.1111/cpr.12891 PMC7653241

[45] Finkelstein, J. D. The metabolism of homocysteine: pathways and regulation. *Eur. J. Pediatr.* **157**, S40–S44 (1998). 9587024 10.1007/pl00014300

[46] Lu, S. C. Regulation of glutathione synthesis. *Mol. Asp. Med.* **30**, 42–59 (2009). 10.1016/j.mam.2008.05.005 PMC2704241 18601945

[47] Gibbs, C. A., Fedoretz-Maxwell, B. P. & Warren, J. J. On the roles of methionine and the importance of its microenvironments in redox metalloproteins. *Dalton Trans.* **51**, 4976–4985 (2022). 35253809 10.1039/d1dt04387k

[48] Schoneich, C. Redox processes of methionine relevant to beta-amyloid oxidation and Alzheimer’s disease. *Arch. Biochem Biophys.* **397**, 370–376 (2002). 11795896 10.1006/abbi.2001.2621

[49] Cantoni, G. Methylation of nicotinamide with a soluble enzyme system from rat liver. *J. Biol. Chem.* **189**, 203-1 (1951). 14832232

[50] Bentley, R. Role of sulfur chirality in the chemical processes of biology. *Chem. Soc. Rev.* **34**, 609–624 (2005). 15965542 10.1039/b418284g

[51] Aposhian, H. V. Enzymatic methylation of arsenic species and other new approaches to arsenic toxicity. *Annu Rev. Pharm. Toxicol.* **37**, 397–419 (1997). 10.1146/annurev.pharmtox.37.1.397 9131259

[52] Fontecave, M., Atta, M. & Mulliez, E. S-adenosylmethionine: nothing goes to waste. *Trends Biochem. Sci.* **29**, 243–249 (2004). 15130560 10.1016/j.tibs.2004.03.007

[53] Satterwhite, E. R. & Mansfield, K. D. RNA methyltransferase METTL16: targets and function. *Wiley Interdiscip. Rev. RNA* **13**, e1681 (2022). 34227247 10.1002/wrna.1681 PMC9286414

[54] Fujimori, D. G. Radical SAM-mediated methylation reactions. *Curr. Opin. Chem. Biol.* **17**, 597–604 (2013). 23835516 10.1016/j.cbpa.2013.05.032 PMC3799849

[55] Jones, P. A. Functions of DNA methylation: islands, start sites, gene bodies and beyond. *Nat. Rev. Genet* **13**, 484–492 (2012). 22641018 10.1038/nrg3230

[56] Meyer, K. D. & Jaffrey, S. R. The dynamic epitranscriptome: N6-methyladenosine and gene expression control. *Nat. Rev. Mol. Cell Biol.* **15**, 313–326 (2014). 24713629 10.1038/nrm3785 PMC4393108

[57] Kouzarides, T. Chromatin modifications and their function. *Cell* **128**, 693–705 (2007). 17320507 10.1016/j.cell.2007.02.005

[58] Soda, K. Polyamine metabolism and gene methylation in conjunction with one-carbon metabolism. *Int. J. Mol. Sci*. **19**, 3106 (2018). 10.3390/ijms19103106 PMC6213949 30309036

[59] Casero, R. A. Jr., Murray Stewart, T. & Pegg, A. E. Polyamine metabolism and cancer: treatments, challenges and opportunities. *Nat. Rev. Cancer* **18**, 681–695 (2018). 30181570 10.1038/s41568-018-0050-3 PMC6487480

[60] Pegg, A. E. & Williams-Ashman, H. G. Phosphate-stimulated breakdown of 5’-methylthioadenosine by rat ventral prostate. *Biochem J.* **115**, 241–247 (1969). 5378381 10.1042/bj1150241 PMC1185095

[61] Madeo, F., Eisenberg, T., Pietrocola, F. & Kroemer, G. Spermidine in health and disease. *Science*. **359**, eaan2788 (2018). 10.1126/science.aan2788 29371440

[62] Pegg, A. E. Functions of polyamines in mammals. *J. Biol. Chem.* **291**, 14904–14912 (2016). 27268251 10.1074/jbc.R116.731661 PMC4946908

[63] Igarashi, K. & Kashiwagi, K. Modulation of cellular function by polyamines. *Int J. Biochem Cell Biol.* **42**, 39–51 (2010). 19643201 10.1016/j.biocel.2009.07.009

[64] Mato, J. M., Martinez-Chantar, M. L. & Lu, S. C. S-adenosylmethionine metabolism and liver disease. *Ann. Hepatol.* **12**, 183–189 (2013). 23396728 PMC4027041

[65] Frostesjo, L. et al. Interference with DNA methyltransferase activity and genome methylation during F9 teratocarcinoma stem cell differentiation induced by polyamine depletion. *J. Biol. Chem.* **272**, 4359–4366 (1997). 9020157 10.1074/jbc.272.7.4359

[66] Dante, R., Arnaud, M. & Niveleau, A. Effects of 5’deoxy-5’-methylthioadenosine on the metabolism of S-adenosyl methionine. *Biochem Biophys. Res. Commun.* **114**, 214–221 (1983). 6309166 10.1016/0006-291x(83)91615-7

[67] Kujubu, D. A. et al. Early responses of PC-12 cells to NGF and EGF: effect of K252a and 5’-methylthioadenosine on gene expression and membrane protein methylation. *J. Neurosci. Res.* **36**, 58–65 (1993). 8230321 10.1002/jnr.490360107

[68] Broderick, J. B., Broderick, W. E. & Hoffman, B. M. Radical SAM enzymes: Nature’s choice for radical reactions. *FEBS Lett.* **597**, 92–101 (2023). 36251330 10.1002/1873-3468.14519 PMC9894703

[69] Broderick, W. E. & Broderick, J. B. Radical SAM enzymes: surprises along the path to understanding mechanism. *J. Biol. Inorg. Chem.* **24**, 769–776 (2019). 31494759 10.1007/s00775-019-01706-w PMC8837180

[70] Vizan, P., Di Croce, L. & Aranda, S. Functional and pathological roles of AHCY. *Front Cell Dev. Biol.* **9**, 654344 (2021). 33869213 10.3389/fcell.2021.654344 PMC8044520

[71] Kloor, D. & Osswald, H. S-Adenosylhomocysteine hydrolase as a target for intracellular adenosine action. *Trends Pharm. Sci.* **25**, 294–297 (2004). 15165742 10.1016/j.tips.2004.04.004

[72] Ingrosso, D. et al. Influence of osmotic stress on protein methylation in resealed erythrocytes. *Eur. J. Biochem.* **244**, 918–922 (1997). 9108266 10.1111/j.1432-1033.1997.00918.x

[73] Mi, J. et al. S-adenosylhomocysteine induces cellular senescence in rat aorta vascular smooth muscle cells via NF-kappaB-SASP pathway. *J. Nutr. Biochem.* **107**, 109063 (2022). 35609855 10.1016/j.jnutbio.2022.109063

[74] You, Y. et al. Inhibition of S-adenosylhomocysteine hydrolase induces endothelial senescence via hTERT downregulation. *Atherosclerosis* **353**, 1–10 (2022). 35753115 10.1016/j.atherosclerosis.2022.06.002

[75] You, Y. et al. Epigenetic modulation of Drp1-mediated mitochondrial fission by inhibition of S-adenosylhomocysteine hydrolase promotes vascular senescence and atherosclerosis. *Redox Biol.* **65**, 102828 (2023). 37517319 10.1016/j.redox.2023.102828 PMC10400927

[76] Wang, Y. et al. Insufficient S-adenosylhomocysteine hydrolase compromises the beneficial effect of diabetic BMSCs on diabetic cardiomyopathy. *Stem Cell Res. Ther.* **13**, 418 (2022). 35964109 10.1186/s13287-022-03099-1 PMC9375418

[77] Hermann, A. & Sitdikova, G. Homocysteine: biochemistry, molecular biology and role in disease. *Biomolecules*. **11**, 737 (2021). 10.3390/biom11050737 PMC8156138 34063494

[78] Raghubeer, S. & Matsha, T. E. Methylenetetrahydrofolate (MTHFR), the one-carbon cycle, and cardiovascular risks. *Nutrients*. **13**, 4562 (2021). 10.3390/nu13124562 PMC8703276 34960114

[79] Zaric, B. L. et al. Homocysteine and hyperhomocysteinaemia. *Curr. Med. Chem.* **26**, 2948–2961 (2019). 29532755 10.2174/0929867325666180313105949

[80] Yang, Z. J. et al. Betaine alleviates cognitive impairment induced by homocysteine through attenuating NLRP3-mediated microglial pyroptosis in an m(6)A-YTHDF2-dependent manner. *Redox Biol.* **69**, 103026 (2024). 38184996 10.1016/j.redox.2024.103026 PMC10808937

[81] Imbard, A. et al. Plasma choline and betaine correlate with serum folate, plasma S-adenosyl-methionine and S-adenosyl-homocysteine in healthy volunteers. *Clin. Chem. Lab. Med.* **51**, 683–692 (2013). 23095202 10.1515/cclm-2012-0302

[82] Bhatia, M. et al. Allosteric inhibition of MTHFR prevents futile SAM cycling and maintains nucleotide pools in one-carbon metabolism. *J. Biol. Chem.* **295**, 16037–16057 (2020). 32934008 10.1074/jbc.RA120.015129 PMC7681022

[83] Prudova, A. et al. S-adenosylmethionine stabilizes cystathionine beta-synthase and modulates redox capacity. *Proc. Natl Acad. Sci. USA* **103**, 6489–6494 (2006). 16614071 10.1073/pnas.0509531103 PMC1458911

[84] Huang, C. F. et al. Echinocystic acid ameliorates hyperhomocysteinemia-induced vascular endothelial cell injury through regulating NF-kappaB and CYP1A1. *Exp. Ther. Med.* **14**, 4174–4180 (2017). 29104633 10.3892/etm.2017.5097 PMC5658691

[85] Ji, C. et al. Propofol alleviates inflammation and apoptosis in HCY‑induced HUVECs by inhibiting endoplasmic reticulum stress. *Mol. Med. Rep*. **23**, 333 (2021). 10.3892/mmr.2021.11972 PMC7974316 33760174

[86] Wu, Y. et al. The expression of SAH, IL-1beta, Hcy, TNF-alpha and BDNF in coronary heart disease and its relationship with the severity of coronary stenosis. *BMC Cardiovasc. Disord.* **22**, 101 (2022). 35282820 10.1186/s12872-021-02388-6 PMC8919521

[87] Jakubowski, H. Homocysteine modification in protein structure/function and human disease. *Physiol. Rev.* **99**, 555–604 (2019). 30427275 10.1152/physrev.00003.2018

[88] Liu, L. et al. Plasma homocysteine and autonomic nervous dysfunction: association and clinical relevance in OSAS. *Dis. Markers* **2020**, 4378505 (2020). 32695242 10.1155/2020/4378505 PMC7368224

[89] Pi, T., Liu, B. & Shi, J. Abnormal homocysteine metabolism: an insight of alzheimer’s disease from DNA methylation. *Behav. Neurol.* **2020**, 8438602 (2020). 32963633 10.1155/2020/8438602 PMC7495165

[90] Park, J. H. et al. Effect of B-vitamins on stroke risk among individuals with vascular disease who are not on antiplatelets: A meta-analysis. *Int J. Stroke* **11**, 206–211 (2016). 26783312 10.1177/1747493015616512

[91] Marti-Carvajal, A. J., Sola, I., Lathyris, D. & Dayer, M. Homocysteine-lowering interventions for preventing cardiovascular events. *Cochrane Database Syst. Rev.* **8**, CD006612 (2017). 28816346 10.1002/14651858.CD006612.pub5 PMC6483699

[92] Lu, S. C. Regulation of hepatic glutathione synthesis. *Semin. Liver Dis.* **18**, 331–343 (1998). 9875552 10.1055/s-2007-1007168

[93] Forman, H. J., Zhang, H. & Rinna, A. Glutathione: overview of its protective roles, measurement, and biosynthesis. *Mol. Asp. Med.* **30**, 1–12 (2009). 10.1016/j.mam.2008.08.006 PMC2696075 18796312

[94] Dixon, S. J. et al. Ferroptosis: an iron-dependent form of nonapoptotic cell death. *Cell* **149**, 1060–1072 (2012). 22632970 10.1016/j.cell.2012.03.042 PMC3367386

[95] Stockwell, B. R. et al. Ferroptosis: a eegulated cell death nexus linking metabolism, redox biology, and disease. *Cell* **171**, 273–285 (2017). 28985560 10.1016/j.cell.2017.09.021 PMC5685180

[96] Koppula, P., Zhuang, L. & Gan, B. Cystine transporter SLC7A11/xCT in cancer: ferroptosis, nutrient dependency, and cancer therapy. *Protein Cell* **12**, 599–620 (2021). 33000412 10.1007/s13238-020-00789-5 PMC8310547

[97] Dixon, S. J. et al. Pharmacological inhibition of cystine-glutamate exchange induces endoplasmic reticulum stress and ferroptosis. *Elife* **3**, e02523 (2014). 24844246 10.7554/eLife.02523 PMC4054777

[98] Huang, C. W. et al. Hydrogen sulfide and its roles in Saccharomyces cerevisiae in a winemaking context. *FEMS Yeast Res*. **17**, 6 (2017). 10.1093/femsyr/fox058 28830086

[99] Simoni, R. D., Hill, R. L. & Vaughan, M. A new sulfur-containing amino-acid isolated from the hydrolytic products of protein(Mueller, J. H. (1923) J. Biol. Chem. 56, 157-169). *J. Biol. Chem.* **277**, 14e (2002). 12068030

[100] Barger, G. & Coyne, F. P. The amino-acid methionine; constitution and synthesis. *Biochem J.* **22**, 1417–1425 (1928). 16744158 10.1042/bj0221417 PMC1252276

[101] Ericson, L. E., Williams, J. N. Jr. & Elvehjem, C. A. Studies on partially purified betaine-homocysteine transmethylase of liver. *J. Biol. Chem.* **212**, 537–544 (1955). 14353854

[102] Gayon, U. J. C. R. A. S. Note de MU. Gayon presentee par M. *Pasteur* **85**, 1074–1076 (1877).

[103] Myers, J. T. The production of hydrogen sulphide by bacteria. *J. Bacteriol.* **5**, 231–252 (1920). 16558873 10.1128/jb.5.3.231-252.1920 PMC378876

[104] Rodler, M., Vadon, V. & Pekar, K. Ability to form H2S in various bacteria. *Zentralbl Bakteriol. Orig.* **206**, 117–122 (1968). 4901958

[105] Massidda, A. Production of H2s in brucella. *Nuovi. Ann. Ig. Microbiol* **15**, 424–431 (1964). 14228927

[106] Clarke, P. H. Hydrogen sulphide production by bacteria. *J. Gen. Microbiol* **8**, 397–407 (1953). 13061742 10.1099/00221287-8-3-397

[107] Stutzenberger, F. J. & Bennett, E. O. Sensitivity of mixed populations of staphylococcus aureus and escherichia coli to mercurials. *Appl. Microbiol.* **13**, 570–574 (1965). 14339264 10.1128/am.13.4.570-574.1965 PMC1058299

[108] Kadota, H. & Ishida, Y. Production of volatile sulfur compounds by microorganisms. *Annu. Rev. Microbiol.* **26**, 127–138 (1972). 4562806 10.1146/annurev.mi.26.100172.001015

[109] Hanson, H. & Eisfeld, G. Intermediary sulfur metabolism. III. Formation of hydrogen sulfide from cystine and cysteine by the liver. *Z. Gesamt. Inn. Med.* **7**, 801–810 (1952). 13006675

[110] Chatagner, F. & Sauret-Ignazi, G. Role of transamination and pyridoxal phosphate in the enzymatic formation of hydrogen sulfide from cysteine by the rat liver under anaerobiosis. *Bull. Soc. Chim. Biol. (Paris)* **38**, 415–428 (1956). 13342749

[111] Hylin, J. W. & Wood, J. L. Enzymatic formation of polysulfides from mercaptopyruvate. *J. Biol. Chem.* **234**, 2141–2144 (1959). 13673028

[112] Yao, K. Effects of several unusual sulfur-containing amino acids on rat liver cystathionine-gamma-lyase. *Physiol. Chem. Phys.* **7**, 401–408 (1975). 1197382

[113] De Cormis, L. J. C. A. S. Release of hydrogen sulfide into an atmosphere containing sulfur dioxide. **266**, 683–685 (1968).

[114] Fenchel, T. & Riedl, R. J. M. B. The sulfide system: a new biotic community underneath the oxidized layer of marine sand bottoms. **7**, 255–268 (1970).

[115] Jannasch, H. W. & Wirsen, C. O. Morphological survey of microbial mats near deep-sea thermal vents. *Appl Environ. Microbiol* **41**, 528–538 (1981). 16345722 10.1128/aem.41.2.528-538.1981 PMC243726

[116] Jannasch, H. W. & Mottl, M. J. Geomicrobiology of deep-sea hydrothermal vents. *Science* **229**, 717–725 (1985). 17841485 10.1126/science.229.4715.717

[117] Gaill, F. Aspects of life development at deep sea hydrothermal vents. *FASEB J.* **7**, 558–565 (1993). 8472894 10.1096/fasebj.7.6.8472894

[118] Sylvan, J. B., Toner, B. M. & Edwards, K. J. Life and death of deep-sea vents: bacterial diversity and ecosystem succession on inactive hydrothermal sulfides. *mBio* **3**, e00279–00211 (2012). 22275502 10.1128/mBio.00279-11 PMC3262234

[119] Sugiyama, E. et al. Precolumn derivatization LC/MS method for observation of efficient hydrogen sulfide supply to the kidney via d-cysteine degradation pathway. *J. Pharm. Biomed. Anal.* **222**, 115088 (2023). 36215804 10.1016/j.jpba.2022.115088

[120] Druzhyna, N. et al. Screening of a composite library of clinically used drugs and well-characterized pharmacological compounds for cystathionine beta-synthase inhibition identifies benserazide as a drug potentially suitable for repurposing for the experimental therapy of colon cancer. *Pharm. Res* **113**, 18–37 (2016). 10.1016/j.phrs.2016.08.016 PMC5107130 27521834

[121] Wang, L. et al. I157172, a novel inhibitor of cystathionine gamma-lyase, inhibits growth and migration of breast cancer cells via SIRT1-mediated deacetylation of STAT3. *Oncol. Rep.* **41**, 427–436 (2019). 30365149 10.3892/or.2018.6798

[122] Liu, Y. H. et al. Hydrogen sulfide in the mammalian cardiovascular system. *Antioxid. Redox Signal* **17**, 141–185 (2012). 22304473 10.1089/ars.2011.4005

[123] Fernandez-Rodriguez, C. et al. Structural basis of the inhibition of cystathionine gamma-lyase from Toxoplasma gondii by propargylglycine and cysteine. *Protein Sci.* **32**, e4619 (2023). 36883335 10.1002/pro.4619 PMC10053738

[124] Yang, Q. & He, G. W. Imbalance of homocysteine and H(2)S: significance, mechanisms, and therapeutic promise in vascular injury. *Oxid. Med. Cell Longev.* **2019**, 7629673 (2019). 31885816 10.1155/2019/7629673 PMC6893243

[125] Giuffre, A. & Vicente, J. B. Hydrogen sulfide biochemistry and interplay with other gaseous mediators in mammalian physiology. *Oxid. Med. Cell Longev.* **2018**, 6290931 (2018). 30050658 10.1155/2018/6290931 PMC6040266

[126] Ascencao, K. & Szabo, C. Emerging roles of cystathionine beta-synthase in various forms of cancer. *Redox Biol.* **53**, 102331 (2022). 35618601 10.1016/j.redox.2022.102331 PMC9168780

[127] Jia, Y. et al. Rational design of a profluorescent substrate for S-adenosylhomocysteine hydrolase and its applications in bioimaging and inhibitor screening. *ACS Appl. Mater. Interfaces* **8**, 25818–25824 (2016). 27626909 10.1021/acsami.6b09190

[128] Henthorn, H. A. & Pluth, M. D. Mechanistic insights into the H(2)S-mediated reduction of aryl azides commonly used in H(2)S detection. *J. Am. Chem. Soc.* **137**, 15330–15336 (2015). 26540330 10.1021/jacs.5b10675 PMC4924530

[129] Shibuya, N. & Kimura, H. Production of hydrogen sulfide from d-cysteine and its therapeutic potential. *Front Endocrinol. (Lausanne)* **4**, 87 (2013). 23882260 10.3389/fendo.2013.00087 PMC3712494

[130] Mikami, Y. et al. Thioredoxin and dihydrolipoic acid are required for 3-mercaptopyruvate sulfurtransferase to produce hydrogen sulfide. *Biochem J.* **439**, 479–485 (2011). 21732914 10.1042/BJ20110841

[131] Rao, S. P., Dobariya, P., Bellamkonda, H. & More, S. S. Role of 3-mercaptopyruvate sulfurtransferase (3-MST) in physiology and disease. *Antioxidants (Basel)*. **12**, 603 (2023). 10.3390/antiox12030603 PMC10045210 36978851

[132] Augsburger, F. & Szabo, C. Potential role of the 3-mercaptopyruvate sulfurtransferase (3-MST)-hydrogen sulfide (H(2)S) pathway in cancer cells. *Pharm. Res.* **154**, 104083 (2020). 10.1016/j.phrs.2018.11.034 30500457

[133] Ye, H. et al. Rational design of a dual-reactive probe for imaging the biogenesis of both H(2)S and GSH from l-Cys rather than d-Cys in live cells. *RSC Chem. Biol.* **3**, 848–852 (2022). 35866170 10.1039/d2cb00105e PMC9257618

[134] Schumann, U. & Subramani, S. Special delivery from mitochondria to peroxisomes. *Trends Cell Biol.* **18**, 253–256 (2008). 18468897 10.1016/j.tcb.2008.04.002 PMC3697091

[135] Suzuki, Y., Saito, J., Munakata, M. & Shibata, Y. Hydrogen sulfide as a novel biomarker of asthma and chronic obstructive pulmonary disease. *Allergol. Int.* **70**, 181–189 (2021). 33214087 10.1016/j.alit.2020.10.003

[136] Insko, M. A. et al. Detection of exhaled hydrogen sulphide gas in rats exposed to intravenous sodium sulphide. *Br. J. Pharm.* **157**, 944–951 (2009). 10.1111/j.1476-5381.2009.00248.x PMC2737653 19422378

[137] Toombs, C. F. et al. Detection of exhaled hydrogen sulphide gas in healthy human volunteers during intravenous administration of sodium sulphide. *Br. J. Clin. Pharm.* **69**, 626–636 (2010). 10.1111/j.1365-2125.2010.03636.x PMC2883755 20565454

[138] Suzuki, N. et al. Induction and inhibition of oral malodor. *Mol. Oral. Microbiol.* **34**, 85–96 (2019). 30927516 10.1111/omi.12259

[139] Jia, J. et al. SQR mediates therapeutic effects of H(2)S by targeting mitochondrial electron transport to induce mitochondrial uncoupling. *Sci. Adv.* **6**, eaaz5752 (2020). 32923620 10.1126/sciadv.aaz5752 PMC7449675

[140] Beltowski, J. Synthesis, metabolism, and signaling mechanisms of hydrogen sulfide: an overview. *Methods Mol. Biol.* **2007**, 1–8 (2019). 31148102 10.1007/978-1-4939-9528-8_1

[141] Landry, A. P., Ballou, D. P. & Banerjee, R. H(2)S oxidation by nanodisc-embedded human sulfide quinone oxidoreductase. *J. Biol. Chem.* **292**, 11641–11649 (2017). 28512131 10.1074/jbc.M117.788547 PMC5512061

[142] Kamoun, P. H2S, a new neuromodulator. *Med. Sci. (Paris)* **20**, 697–700 (2004). 15329822 10.1051/medsci/2004206-7697

[143] Weisiger, R. A., Pinkus, L. M. & Jakoby, W. B. Thiol S-methyltransferase: suggested role in detoxication of intestinal hydrogen sulfide. *Biochem. Pharm.* **29**, 2885–2887 (1980). 7437088 10.1016/0006-2952(80)90029-5

[144] Weisiger, R. A. & Jakoby, W. B. Thiol S-methyltransferase from rat liver. *Arch. Biochem. Biophys.* **196**, 631–637 (1979). 485170 10.1016/0003-9861(79)90317-5

[145] Hiemke, C. & Ghraf, R. Distribution and properties of thiol S-methyltransferase in rat brain. *J. Neurochem.* **40**, 592–594 (1983). 6822842 10.1111/j.1471-4159.1983.tb11325.x

[146] Jensen, B. & Fago, A. Reactions of ferric hemoglobin and myoglobin with hydrogen sulfide under physiological conditions. *J. Inorg. Biochem.* **182**, 133–140 (2018). 29459272 10.1016/j.jinorgbio.2018.02.007

[147] Suzuki, Y. et al. Methemoglobin-albumin clusters for the treatment of hydrogen sulfide intoxication. *J. Control Release* **349**, 304–314 (2022). 35809661 10.1016/j.jconrel.2022.07.001

[148] Lin, K. et al. Disrupted methionine cycle triggers muscle atrophy in cancer cachexia through epigenetic regulation of REDD1. *Cell Metab.* **37**, 460–476.E8 (2024). 10.1016/j.cmet.2024.10.017 39729999

[149] Lu, Y. et al. Lactylation-driven IGF2BP3-mediated serine metabolism reprogramming and RNA m6A-modification promotes lenvatinib resistance in HCC. *Adv. Sci. (Weinh.)* **11**, e2401399 (2024). 39450426 10.1002/advs.202401399 PMC11633555

[150] Verdejo-Torres, O. et al. Cysteine rich intestinal protein 2 is a copper-responsive regulator of skeletal muscle differentiation and metal homeostasis. *PLoS Genet* **20**, e1011495 (2024). 39637238 10.1371/journal.pgen.1011495 PMC11671023

[151] Yu, Z. J. et al. Gold nanoparticles-based colorimetric immunoassay of carcinoembryonic antigen with metal-organic framework to load quinones for catalytic oxidation of cysteine. *Sensors (Basel)*. **24**, 6701 (2024). 10.3390/s24206701 PMC11510933 39460180

[152] Huang, Y. Q. et al. Interaction among hydrogen sulfide and other gasotransmitters in mammalian physiology and pathophysiology. *Adv. Exp. Med. Biol.* **1315**, 205–236 (2021). 34302694 10.1007/978-981-16-0991-6_9

[153] Irvine, J. C., Ravi, R. M., Kemp-Harper, B. K. & Widdop, R. E. Nitroxyl donors retain their depressor effects in hypertension. *Am. J. Physiol. Heart Circ. Physiol.* **305**, H939–H945 (2013). 23851276 10.1152/ajpheart.00630.2012 PMC3761337

[154] Mancardi, D. et al. HNO protects the myocardium against reperfusion injury, inhibiting the mPTP opening via PKCepsilon activation. *Antioxidants (Basel)*. **11**, 382 (2022). 10.3390/antiox11020382 PMC8869498 35204265

[155] Kimura, H. Hydrogen sulfide (H(2)S)/polysulfides (H(2)S(n)) signalling and TRPA1 channels modification on sulfur metabolism. *Biomolecules*. **14**, 129 (2024). 10.3390/biom14010129 PMC10813152 38275758

[156] Peleli, M., Zampas, P. & Papapetropoulos, A. Hydrogen sulfide and the kidney: physiological roles, contribution to pathophysiology, and therapeutic potential. *Antioxid. Redox Signal* **36**, 220–243 (2022). 34978847 10.1089/ars.2021.0014

[157] Zaorska, E. et al. Hydrogen sulfide in pharmacotherapy, beyond the hydrogen sulfide-donors. *Biomolecules*. **10**, 323 (2020). 10.3390/biom10020323 PMC7072623 32085474

[158] Webb, G. D. et al. Contractile and vasorelaxant effects of hydrogen sulfide and its biosynthesis in the human internal mammary artery. *J. Pharm. Exp. Ther.* **324**, 876–882 (2008). 10.1124/jpet.107.133538 18029544

[159] Sheibani, L. et al. Augmented H2S production via cystathionine-beta-synthase upregulation plays a role in pregnancy-associated uterine vasodilation. *Biol. Reprod.* **96**, 664–672 (2017). 28339573 10.1095/biolreprod.116.143834 PMC6366540

[160] Song, H. B. et al. Significance of serum NT-proBNP and endogenous H(2)S for predicting coronary artery lesions in pediatric kawasaki disease. *J. Coll. Physicians Surg. Pak.* **30**, 37–40 (2020). 31931930 10.29271/jcpsp.2020.01.37

[161] Siegel, L. M. A Direct microdetermination for sulfide. *Anal. Biochem.* **11**, 126–12 (1965). 14328633 10.1016/0003-2697(65)90051-5

[162] Brown, M. D., Hall, J. R. & Schoenfisch, M. H. A direct and selective electrochemical hydrogen sulfide sensor. *Anal. Chim. Acta.* **1045**, 67–76 (2019). 30454574 10.1016/j.aca.2018.08.054 PMC6641862

[163] Li, X. H., Zhang, C. Y., Wu, J. X. & Zhang, T. Changes in plasma hydrogen sulfide and nitric oxide levels and their clinical significance in children with Kawasaki disease. *Chin. Med. J. (Engl.)* **124**, 3445–3449 (2011). 22340156

[164] Yang, G. et al. H2S as a physiologic vasorelaxant: hypertension in mice with deletion of cystathionine gamma-lyase. *Science* **322**, 587–590 (2008). 18948540 10.1126/science.1162667 PMC2749494

[165] Zhang, L. et al. Ag(2)S/Ag nanoparticle microelectrodes for in vivo potentiometric measurement of hydrogen sulfide dynamics in the rat brain. *Anal. Chem.* **93**, 7063–7070 (2021). 33900732 10.1021/acs.analchem.1c00540

[166] Zhen, Y. et al. Exogenous hydrogen sulfide exerts proliferation/anti-apoptosis/angiogenesis/migration effects via amplifying the activation of NF-kappaB pathway in PLC/PRF/5 hepatoma cells. *Int. J. Oncol.* **46**, 2194–2204 (2015). 25738635 10.3892/ijo.2015.2914

[167] Velazquez-Moyado, J. A. & Navarrete, A. The detection and quantification, in vivo and in real time, of hydrogen sulfide in ethanol-induced lesions in rat stomachs using an ion sensitive electrode. *J. Pharm. Toxicol. Methods* **89**, 54–58 (2018). 10.1016/j.vascn.2017.10.008 29100966

[168] Tian, X., Li, Z., Lau, C. & Lu, J. Visualization of in vivo hydrogen sulfide production by a bioluminescence probe in cancer cells and nude mice. *Anal. Chem.* **87**, 11325–11331 (2015). 26482557 10.1021/acs.analchem.5b03712

[169] Olson, K. R., Gao, Y. & Straub, K. D. Oxidation of hydrogen sulfide by quinones: how polyphenols initiate their cytoprotective effects. *Int. J. Mol. Sci*. **22**, 961 (2021). 10.3390/ijms22020961 PMC7835830 33478045

[170] Wang, L. Y., Chen, X. G. & Cao, D. R. A nitroolefin functionalized DPP fluorescent probe for the selective detection of hydrogen sulfide. *N. J. Chem.* **41**, 3367–3373 (2017).

[171] Liu, C. R. et al. Capture and visualization of hydrogen sulfide by a fluorescent probe. *Angew. Chem. Int. Ed.* **50**, 10327–10329 (2011). 10.1002/anie.201104305 PMC3417056 21898737

[172] Peng, B. et al. Fluorescent probes based on nucleophilic substitution-cyclization for hydrogen sulfide detection and bioimaging. *Chem.-Eur. J.* **20**, 1010–1016 (2014). 24339269 10.1002/chem.201303757 PMC4049170

[173] Liu, C. R. et al. Reaction based fluorescent probes for hydrogen sulfide. *Org. Lett.* **14**, 2184–2187 (2012). 22486842 10.1021/ol3008183 PMC3336739

[174] Singha, S. et al. Toward a selective, sensitive, fast-responsive, and biocompatible two-photon probe for hydrogen sulfide in live cells. *Anal. Chem.* **87**, 1188–1195 (2015). 25495776 10.1021/ac503806w

[175] Wu, Q., Huo, F. J., Wang, J. P. & Yin, C. X. Fluorescent probe for detecting hydrogen sulfide based on disulfide nucleophilic substitution-addition. *Spectrochim. Acta. A*. **238**, 118437 (2020). 10.1016/j.saa.2020.118437 32388415

[176] Liu, J. et al. Molecular engineering of aqueous soluble triarylboron-compound-based two-photon fluorescent probe for mitochondria H2S with analyte-induced finite aggregation and excellent membrane permeability. *Anal. Chem.* **88**, 1052–1057 (2016). 26634883 10.1021/acs.analchem.5b04248

[177] Chen, L. L., Huang, P. H., Tan, H. L. & Wang, L. A terbium(III)-based coordination polymer for time-resolved determination of hydrogen sulfide in human serum displacement of copper(II). *Anal. Methods-Uk* **9**, 1004–1010 (2017).

[178] Moretti, M. et al. Fatal poisoning of four workers in a farm: distribution of hydrogen sulfide and thiosulfate in 10 different biological matrices. *Forensic Sci. Int.* **316**, 110525 (2020). 33039903 10.1016/j.forsciint.2020.110525

[179] Vitvitsky, V. & Banerjee, R. H2S analysis in biological samples using gas chromatography with sulfur chemiluminescence detection. *Methods Enzymol.* **554**, 111–123 (2015). 25725519 10.1016/bs.mie.2014.11.013 PMC4684085

[180] Shen, X. et al. Measurement of plasma hydrogen sulfide in vivo and in vitro. *Free Radic. Biol. Med.* **50**, 1021–1031 (2011). 21276849 10.1016/j.freeradbiomed.2011.01.025 PMC4798232

[181] Klingerman, C. M., Trushin, N., Prokopczyk, B. & Haouzi, P. H2S concentrations in the arterial blood during H2S administration in relation to its toxicity and effects on breathing. *Am. J. Physiol. Regul. Integr. Comp. Physiol.* **305**, R630–R638 (2013). 23904109 10.1152/ajpregu.00218.2013 PMC3763045

[182] Luo, S. et al. Endothelial HDAC1-ZEB2-NuRD complex drives aortic aneurysm and dissection through regulation of protein S-Sulfhydration. *Circulation* **147**, 1382–1403 (2023). 36951067 10.1161/CIRCULATIONAHA.122.062743

[183] Weng, L. et al. TGF-beta1/SMAD3 regulates programmed cell death 5 that suppresses cardiac fibrosis post-myocardial infarction by inhibiting HDAC3. *Circ. Res.* **133**, 237–251 (2023). 37345556 10.1161/CIRCRESAHA.123.322596

[184] Yuan, J. et al. Microneedle patch loaded with exosomes containing microRNA-29b prevents cardiac fibrosis after myocardial infarction. *Adv. Health. Mater.* **12**, e2202959 (2023). 10.1002/adhm.202202959 36739582

[185] Micheletti, R. et al. The long noncoding RNA Wisper controls cardiac fibrosis and remodeling. *Sci. Transl. Med*. **9**, eaai9118 (2017). 10.1126/scitranslmed.aai9118 PMC5643582 28637928

[186] Zhao, K. et al. Alamandine alleviated heart failure and fibrosis in myocardial infarction mice. *Biol. Direct* **17**, 25 (2022). 36167556 10.1186/s13062-022-00338-6 PMC9516792

[187] Zhao, Q. et al. Metformin decreased myocardial fibrosis and apoptosis in hyperhomocysteinemia -induced cardiac hypertrophy. *Curr. Res Transl. Med.* **69**, 103270 (2021). 33268288 10.1016/j.retram.2020.103270

[188] Nam, M., Jung, Y., Ryu, D. H. & Hwang, G. S. A metabolomics-driven approach reveals metabolic responses and mechanisms in the rat heart following myocardial infarction. *Int. J. Cardiol.* **227**, 239–246 (2017). 27852445 10.1016/j.ijcard.2016.11.127

[189] Li, Y. et al. Exogenous hydrogen sulfide inhibits apoptosis by regulating endoplasmic reticulum stress-autophagy axis and improves myocardial reconstruction after acute myocardial infarction. *Acta Biochim Biophys. Sin. (Shanghai)* **52**, 1325–1336 (2020). 33210714 10.1093/abbs/gmaa133

[190] Guo, Z. et al. CSE/H2S system protects mesenchymal stem cells from hypoxia and serum deprivation‑induced apoptosis via mitochondrial injury, endoplasmic reticulum stress and PI3K/Akt activation pathways. *Mol. Med. Rep.* **12**, 2128–2134 (2015). 25901909 10.3892/mmr.2015.3651

[191] Yang, T. et al. AP39 inhibits ferroptosis by inhibiting mitochondrial autophagy through the PINK1/parkin pathway to improve myocardial fibrosis with myocardial infarction. *Biomed. Pharmacother.* **165**, 115195 (2023). 37516015 10.1016/j.biopha.2023.115195

[192] Angelovski, M. et al. Myocardial infarction and oxidative damage in animal models: objective and expectations from the application of cysteine derivatives. *Toxicol. Mech. Methods* **33**, 1–17 (2023). 35450505 10.1080/15376516.2022.2069530

[193] Nishizawa, H. et al. Ferroptosis is controlled by the coordinated transcriptional regulation of glutathione and labile iron metabolism by the transcription factor BACH1. *J. Biol. Chem.* **295**, 69–82 (2020). 31740582 10.1074/jbc.RA119.009548 PMC6952604

[194] Gao, E. et al. A novel and efficient model of coronary artery ligation and myocardial infarction in the mouse. *Circ. Res.* **107**, 1445–1453 (2010). 20966393 10.1161/CIRCRESAHA.110.223925 PMC3005817

[195] Chen, C. L. et al. Mitochondrial redox regulation and myocardial ischemia-reperfusion injury. *Am. J. Physiol. Cell Physiol.* **322**, C12–C23 (2022). 34757853 10.1152/ajpcell.00131.2021 PMC8721908

[196] Lee, H. L. et al. Biphasic modulation of the mitochondrial electron transport chain in myocardial ischemia and reperfusion. *Am. J. Physiol. Heart Circ. Physiol.* **302**, H1410–H1422 (2012). 22268109 10.1152/ajpheart.00731.2011 PMC3330792

[197] Jang, S. et al. Elucidating mitochondrial electron transport chain supercomplexes in the heart during ischemia-reperfusion. *Antioxid. Redox Signal* **27**, 57–69 (2017). 27604998 10.1089/ars.2016.6635 PMC5488255

[198] Hashmi, S. & Al-Salam, S. Acute myocardial infarction and myocardial ischemia-reperfusion injury: a comparison. *Int. J. Clin. Exp. Pathol.* **8**, 8786–8796 (2015). 26464621 PMC4583853

[199] Li, N. et al. Chemotactic NO/H(2)S nanomotors realizing cardiac targeting of G-CSF against myocardial ischemia-reperfusion injury. *ACS Nano* **17**, 12573–12593 (2023). 37327056 10.1021/acsnano.3c02781

[200] Xu, S. et al. Macrophage heterogeneity and its impact on myocardial ischemia-reperfusion injury: An Integrative Review. *J. Inflamm. Res.* **16**, 5971–5987 (2023). 38088942 10.2147/JIR.S436560 PMC10712254

[201] Liu, X. et al. Actin cytoskeleton vulnerability to disulfide stress mediates disulfidptosis. *Nat. Cell Biol.* **25**, 404–414 (2023). 36747082 10.1038/s41556-023-01091-2 PMC10027392

[202] Qian, S. et al. Disulfide stress and its role in cardiovascular diseases. *Redox Biol.* **75**, 103297 (2024). 39127015 10.1016/j.redox.2024.103297 PMC11364009

[203] Rashdan, N. A., Shrestha, B. & Pattillo, C. B. S-glutathionylation, friend or foe in cardiovascular health and disease. *Redox Biol.* **37**, 101693 (2020). 32912836 10.1016/j.redox.2020.101693 PMC7767732

[204] Wang, L., Niu, H. & Zhang, J. Homocysteine induces mitochondrial dysfunction and oxidative stress in myocardial ischemia/reperfusion injury through stimulating ROS production and the ERK1/2 signaling pathway. *Exp. Ther. Med.* **20**, 938–944 (2020). 32742337 10.3892/etm.2020.8735 PMC7388298

[205] Pan, Y. et al. Dietary methionine restriction attenuates renal ischaemia/reperfusion-induced myocardial injury by activating the CSE/H2S/ERS pathway in diabetic mice. *J. Cell Mol. Med.* **24**, 9890–9897 (2020). 32790060 10.1111/jcmm.15578 PMC7520309

[206] Lazarevic, M. et al. The H(2)S donor GYY4137 stimulates reactive oxygen species generation in BV2 cells while suppressing the secretion of TNF and Nitric Oxide. *Molecules*. **23**, 2966 (2018). 10.3390/molecules23112966 PMC6278327 30441775

[207] Elrod, J. W. et al. Hydrogen sulfide attenuates myocardial ischemia-reperfusion injury by preservation of mitochondrial function. *Proc. Natl Acad. Sci. USA* **104**, 15560–15565 (2007). 17878306 10.1073/pnas.0705891104 PMC2000503

[208] Luo, Y. et al. Activation of the CaR-CSE/H2S pathway confers cardioprotection against ischemia-reperfusion injury. *Exp. Cell Res.* **398**, 112389 (2021). 33221316 10.1016/j.yexcr.2020.112389

[209] Meng, G. et al. GYY4137 protects against myocardial ischemia and reperfusion injury by attenuating oxidative stress and apoptosis in rats. *J. Biomed. Res.* **29**, 203–213 (2015). 26060444 10.7555/JBR.28.20140037 PMC4449488

[210] Xie, H. et al. Hydrogen sulfide protects against myocardial ischemia and reperfusion injury by activating AMP-activated protein kinase to restore autophagic flux. *Biochem. Biophys. Res. Commun.* **458**, 632–638 (2015). 25684185 10.1016/j.bbrc.2015.02.017

[211] Gimbrone, M. A. Jr. & Garcia-Cardena, G. Endothelial cell dysfunction and the pathobiology of atherosclerosis. *Circ. Res.* **118**, 620–636 (2016). 26892962 10.1161/CIRCRESAHA.115.306301 PMC4762052

[212] Xu, S. et al. Endothelial dysfunction in atherosclerotic cardiovascular diseases and beyond: from mechanism to pharmacotherapies. *Pharm. Rev.* **73**, 924–967 (2021). 34088867 10.1124/pharmrev.120.000096

[213] Back, M. et al. Inflammation and its resolution in atherosclerosis: mediators and therapeutic opportunities. *Nat. Rev. Cardiol.* **16**, 389–406 (2019). 30846875 10.1038/s41569-019-0169-2 PMC6727648

[214] Dhar, I. et al. Plasma methionine and risk of acute myocardial infarction: Effect modification by established risk factors. *Atherosclerosis* **272**, 175–181 (2018). 29621698 10.1016/j.atherosclerosis.2018.03.038

[215] Dai, X. et al. Betaine supplementation attenuates S-adenosylhomocysteine hydrolase-deficiency-accelerated atherosclerosis in apolipoprotein E-deficient mice. *Nutrients*. **14**, 718 (2022). 10.3390/nu14030718 PMC8840105 35277077

[216] Roman, M. J. et al. Rate and determinants of progression of atherosclerosis in systemic lupus erythematosus. *Arthritis Rheum.* **56**, 3412–3419 (2007). 17907140 10.1002/art.22924

[217] Munteanu, C. Hydrogen sulfide and oxygen homeostasis in atherosclerosis: a systematic review from molecular biology to therapeutic perspectives. *Int. J. Mol. Sci*. **24**, 8376 (2023). 10.3390/ijms24098376 PMC10179092 37176083

[218] Bai, Z. et al. Anti-atherosclerosis effect of H(2)S donors based on nicotinic acid and chlorfibrate structures. *Bioorg. Med. Chem.* **27**, 3307–3318 (2019). 31204228 10.1016/j.bmc.2019.06.012

[219] Ford, A., Al-Magableh, M., Gaspari, T. A. & Hart, J. L. Chronic NaHS treatment is vasoprotective in high-fat-fed ApoE(-/-) mice. *Int J. Vasc. Med.* **2013**, 915983 (2013). 23864951 10.1155/2013/915983 PMC3707268

[220] Tian, D. et al. Endogenous hydrogen sulfide improves vascular remodeling through PPARdelta/SOCS3 signaling. *J. Adv. Res.* **27**, 115–125 (2021). 33318871 10.1016/j.jare.2020.06.005 PMC7728593

[221] Nakamura, M. & Sadoshima, J. Mechanisms of physiological and pathological cardiac hypertrophy. *Nat. Rev. Cardiol.* **15**, 387–407 (2018). 29674714 10.1038/s41569-018-0007-y

[222] Tham, Y. K. et al. Pathophysiology of cardiac hypertrophy and heart failure: signaling pathways and novel therapeutic targets. *Arch. Toxicol.* **89**, 1401–1438 (2015). 25708889 10.1007/s00204-015-1477-x

[223] Nomura, S. et al. Cardiomyocyte gene programs encoding morphological and functional signatures in cardiac hypertrophy and failure. *Nat. Commun.* **9**, 4435 (2018). 30375404 10.1038/s41467-018-06639-7 PMC6207673

[224] Deng, Y. et al. Hyperhomocysteinemia promotes cardiac hypertrophy in hypertension. *Oxid. Med Cell Longev.* **2022**, 1486157 (2022). 36046692 10.1155/2022/1486157 PMC9423973

[225] Kar, S. et al. Hydrogen sulfide ameliorates homocysteine-induced cardiac remodeling and dysfunction. *Front Physiol.* **10**, 598 (2019). 31178749 10.3389/fphys.2019.00598 PMC6544124

[226] Lu, F. et al. Exogenous hydrogen sulfide prevents cardiomyocyte apoptosis from cardiac hypertrophy induced by isoproterenol. *Mol. Cell Biochem.* **381**, 41–50 (2013). 23660955 10.1007/s11010-013-1686-7

[227] Ellmers, L. J. et al. Hydrogen sulfide treatment improves post-infarct remodeling and long-term cardiac function in CSE knockout and wild-type mice. *Int. J. Mol. Sci*. **21**, 4284 (2020). 10.3390/ijms21124284 PMC7352717 32560137

[228] Peleli, M. et al. Cardiovascular phenotype of mice lacking 3-mercaptopyruvate sulfurtransferase. *Biochem. Pharm.* **176**, 113833 (2020). 32027885 10.1016/j.bcp.2020.113833 PMC7657663

[229] Liang, M. et al. Hydrogen sulfide improves glucose metabolism and prevents hypertrophy in cardiomyocytes. *Nitric Oxide* **46**, 114–122 (2015). 25524832 10.1016/j.niox.2014.12.007

[230] Borst, M. M., Deussen, A. & Schrader, J. S-adenosylhomocysteine hydrolase activity in human myocardium. *Cardiovasc. Res.* **26**, 143–147 (1992). 1571934 10.1093/cvr/26.2.143

[231] Waypa, G. B. et al. Mitochondria regulate proliferation in adult cardiac myocytes. *J. Clin. Invest*. **134**, e165482 (2024). 10.1172/JCI165482 PMC11213516 38722697

[232] Rong, L. et al. Advancements in the treatment of non-alcoholic fatty liver disease (NAFLD). *Front Endocrinol. (Lausanne)* **13**, 1087260 (2022). 36726464 10.3389/fendo.2022.1087260 PMC9884828

[233] Heeren, J. & Scheja, L. Metabolic-associated fatty liver disease and lipoprotein metabolism. *Mol. Metab.* **50**, 101238 (2021). 33892169 10.1016/j.molmet.2021.101238 PMC8324684

[234] Gross, B., Pawlak, M., Lefebvre, P. & Staels, B. PPARs in obesity-induced T2DM, dyslipidaemia and NAFLD. *Nat. Rev. Endocrinol.* **13**, 36–49 (2017). 27636730 10.1038/nrendo.2016.135

[235] Xu, W. et al. Hepatocellular cystathionine gamma lyase/hydrogen sulfide attenuates nonalcoholic fatty liver disease by activating farnesoid X receptor. *Hepatology* **76**, 1794–1810 (2022). 35586979 10.1002/hep.32577 PMC9795901

[236] Yu, Y., Ye, S. M., Liu, D. Y. & Yang, L. Q. AP39 ameliorates high fat diet-induced liver injury in young rats via alleviation of oxidative stress and mitochondrial impairment. *Exp. Anim.* **70**, 553–562 (2021). 34305077 10.1538/expanim.21-0056 PMC8614011

[237] Alshawsh, M. A. et al. A comparison of the gene expression profiles of non-alcoholic fatty liver disease between animal models of a high-fat diet and methionine-choline-deficient diet. *Molecules*. **27**, 858 (2022). 10.3390/molecules27030858 PMC8839835 35164140

[238] Wang, Q. et al. Naringenin attenuates non-alcoholic fatty liver disease by down-regulating the NLRP3/NF-kappaB pathway in mice. *Br. J. Pharm.* **177**, 1806–1821 (2020). 10.1111/bph.14938 PMC7070172 31758699

[239] Park, H. S. et al. TXNIP/VDUP1 attenuates steatohepatitis via autophagy and fatty acid oxidation. *Autophagy* **17**, 2549–2564 (2021). 33190588 10.1080/15548627.2020.1834711 PMC8496541

[240] Balkrishna, A. et al. Livogrit prevents methionine-cystine deficiency induced nonalcoholic steatohepatitis by modulation of steatosis and oxidative stress in human hepatocyte-derived spheroid and in primary rat hepatocytes. *Bioengineered* **13**, 10811–10826 (2022). 35485140 10.1080/21655979.2022.2065789 PMC9208489

[241] Lu, S. C. & Mato, J. M. S-adenosylmethionine in liver health, injury, and cancer. *Physiol. Rev.* **92**, 1515–1542 (2012). 23073625 10.1152/physrev.00047.2011 PMC3698976

[242] Pascale, R. M. et al. S-Adenosylmethionine: from the discovery of its inhibition of tumorigenesis to its use as a therapeutic agent. *Cells*. **11**, 409 (2022). 10.3390/cells11030409 PMC8834208 35159219

[243] Lu, S. C. et al. Methionine adenosyltransferase 1A knockout mice are predisposed to liver injury and exhibit increased expression of genes involved in proliferation. *Proc. Natl Acad. Sci. USA* **98**, 5560–5565 (2001). 11320206 10.1073/pnas.091016398 PMC33252

[244] Asrani, S. K., Devarbhavi, H., Eaton, J. & Kamath, P. S. Burden of liver diseases in the world. *J. Hepatol.* **70**, 151–171 (2019). 30266282 10.1016/j.jhep.2018.09.014

[245] Rodriguez, M. J. et al. Maresin-1 prevents liver fibrosis by targeting Nrf2 and NF-kappaB, reducing oxidative stress and inflammation. *Cells*. **10**, 3406 (2021). 10.3390/cells10123406 PMC8699629 34943914

[246] Aljobaily, N. et al. Creatine alleviates doxorubicin-induced liver aamage by inhibiting liver fibrosis, inflammation, oxidative stress, and cellular senescence. *Nutrients*. **13**, 41 (2020). 10.3390/nu13010041 PMC7824063 33374297

[247] Tripathi, M. et al. Vitamin B(12) and folate decrease inflammation and fibrosis in NASH by preventing syntaxin 17 homocysteinylation. *J. Hepatol.* **77**, 1246–1255 (2022). 35820507 10.1016/j.jhep.2022.06.033

[248] Mohammadian, Z. et al. Effects of folic acid on dyslipidemia and serum homocysteine in a rat model of cholestasis and hepatic fibrosis. *Pol. J. Pathol.* **66**, 49–56 (2015). 26017880 10.5114/pjp.2015.51153

[249] Fiorucci, S., Distrutti, E., Cirino, G. & Wallace, J. L. The emerging roles of hydrogen sulfide in the gastrointestinal tract and liver. *Gastroenterology* **131**, 259–271 (2006). 16831608 10.1053/j.gastro.2006.02.033

[250] Zhao, S. et al. Hydrogen sulfide alleviates liver injury through the S-sulfhydrated-kelch-like ECH-associated protein 1/Nuclear erythroid 2-related factor 2/low-density lipoprotein receptor-related protein 1 pathway. *Hepatology* **73**, 282–302 (2021). 32219872 10.1002/hep.31247

[251] Gong, Z. et al. S-allyl-cysteine attenuates carbon tetrachloride-induced liver fibrosis in rats by targeting STAT3/SMAD3 pathway. *Am. J. Transl. Res.* **10**, 1337–1346 (2018). 29887949 PMC5992555

[252] Peng, X. et al. Resveratrol improves synaptic plasticity in hypoxic-ischemic brain injury in neonatal mice via alleviating SIRT1/NF-kappaB signaling-mediated neuroinflammation. *J. Mol. Neurosci.* **72**, 113–125 (2022). 34549339 10.1007/s12031-021-01908-5

[253] Barks, J. D. E., Liu, Y., Dopp, I. A. & Silverstein, F. S. Azithromycin reduces inflammation-amplified hypoxic-ischemic brain injury in neonatal rats. *Pediatr. Res.* **92**, 415–423 (2022). 34625655 10.1038/s41390-021-01747-5 PMC8989723

[254] Xu, X. et al. Irisin prevents hypoxic-ischemic brain damage in rats by inhibiting oxidative stress and protecting the blood-brain barrier. *Peptides* **161**, 170945 (2023). 36623553 10.1016/j.peptides.2023.170945

[255] Liu, Y. et al. Downregulation of nitric oxide by electroacupuncture against hypoxic‑ischemic brain damage in rats via nuclear factor‑kappaB/neuronal nitric oxide synthase. *Mol. Med. Rep.* **11**, 837–842 (2015). 25374015 10.3892/mmr.2014.2879 PMC4262503

[256] Tagliari, B. et al. Hyperhomocysteinemia increases damage on brain slices exposed to in vitro model of oxygen and glucose deprivation: prevention by folic acid. *Int J. Dev. Neurosci.* **24**, 285–291 (2006). 16542814 10.1016/j.ijdevneu.2006.01.002

[257] Fogal, B. et al. System x(c)- activity and astrocytes are necessary for interleukin-1 beta-mediated hypoxic neuronal injury. *J. Neurosci.* **27**, 10094–10105 (2007). 17881516 10.1523/JNEUROSCI.2459-07.2007 PMC6672668

[258] Liu, C. Y. et al. The role of cystathionine-beta-synthase, H(2)S, and miRNA-377 in hypoxic-ischemic encephalopathy: insights from human and animal studies. *J. Mol. Neurosci.* **73**, 921–931 (2023). 37864623 10.1007/s12031-023-02165-4

[259] Xin, D. et al. l-Cysteine suppresses hypoxia-ischemia injury in neonatal mice by reducing glial activation, promoting autophagic flux and mediating synaptic modification via H(2)S formation. *Brain Behav. Immun.* **73**, 222–234 (2018). 29751053 10.1016/j.bbi.2018.05.007

[260] Yuan, Q. et al. Anti-cerebral ischemia reperfusion injury of polysaccharides: A review of the mechanisms. *Biomed. Pharmacother.* **137**, 111303 (2021). 33517189 10.1016/j.biopha.2021.111303

[261] Xu, D. et al. Orexin-A alleviates astrocytic apoptosis and inflammation via inhibiting OX1R-mediated NF-kappaB and MAPK signaling pathways in cerebral ischemia/reperfusion injury. *Biochim Biophys. Acta Mol. Basis Dis.* **1867**, 166230 (2021). 34358627 10.1016/j.bbadis.2021.166230

[262] Zhu, K. et al. Inhibition of TLR4 prevents hippocampal hypoxic-ischemic injury by regulating ferroptosis in neonatal rats. *Exp. Neurol.* **345**, 113828 (2021). 34343528 10.1016/j.expneurol.2021.113828

[263] Rao, A. M. et al. Beneficial effects of S-adenosyl-L-methionine on blood-brain barrier breakdown and neuronal survival after transient cerebral ischemia in gerbils. *Brain Res. Mol. Brain Res.* **44**, 134–138 (1997). 9030707 10.1016/s0169-328x(96)00245-8

[264] Zhao, Y. et al. Homocysteine aggravates cortical neural cell injury through neuronal autophagy overactivation following rat cerebral ischemia-reperfusion. *Int. J. Mol. Sci*. **17**, 1196 (2016). 10.3390/ijms17081196 PMC5000594 27455253

[265] Petras, M. et al. Effect of hyperhomocysteinemia on redox balance and redox defence enzymes in ischemia-reperfusion injury and/or after ischemic preconditioning in rats. *Cell Mol. Neurobiol.* **37**, 1417–1431 (2017). 28210876 10.1007/s10571-017-0473-5 PMC11482144

[266] Villalobos, M. A. et al. Effect of S-adenosyl-L-methionine on rat brain oxidative stress damage in a combined model of permanent focal ischemia and global ischemia-reperfusion. *Brain Res.* **883**, 31–40 (2000). 11063985 10.1016/s0006-8993(00)02873-0

[267] Deng, G. et al. Protective effect of hydrogen sulfide on cerebral ischemia-reperfusion injury. *Cell Mol. Neurobiol.* **43**, 15–25 (2023). 35066714 10.1007/s10571-021-01166-4 PMC11415178

[268] Jiang, W. W. et al. Sodium hydrosulfide attenuates cerebral ischemia/reperfusion injury by suppressing overactivated autophagy in rats. *FEBS Open Bio.* **7**, 1686–1695 (2017). 29123977 10.1002/2211-5463.12301 PMC5666398

[269] Ji, K., Xue, L., Cheng, J. & Bai, Y. Preconditioning of H2S inhalation protects against cerebral ischemia/reperfusion injury by induction of HSP70 through PI3K/Akt/Nrf2 pathway. *Brain Res. Bull.* **121**, 68–74 (2016). 26772627 10.1016/j.brainresbull.2015.12.007

[270] Zhang, Y. et al. CSE-derived H(2)S inhibits reactive astrocytes proliferation and promotes neural functional recovery after cerebral ischemia/reperfusion injury in mice via inhibition of RhoA/ROCK(2) pathway. *ACS Chem. Neurosci.* **12**, 2580–2590 (2021). 34252278 10.1021/acschemneuro.0c00674

[271] Zhang, X. & Bian, J. S. Hydrogen sulfide: a neuromodulator and neuroprotectant in the central nervous system. *ACS Chem. Neurosci.* **5**, 876–883 (2014). 25230373 10.1021/cn500185g

[272] Wang, Z. et al. L-Cysteine promotes the proliferation and differentiation of neural stem cells via the CBS/H(2)S pathway. *Neuroscience* **237**, 106–117 (2013). 23376738 10.1016/j.neuroscience.2012.12.057

[273] Sun, X. Y. et al. Rutin prevents tau pathology and neuroinflammation in a mouse model of Alzheimer’s disease. *J. Neuroinflamm.* **18**, 131 (2021). 10.1186/s12974-021-02182-3 PMC8196535 34116706

[274] Congdon, E. E. & Sigurdsson, E. M. Tau-targeting therapies for Alzheimer disease. *Nat. Rev. Neurol.* **14**, 399–415 (2018). 29895964 10.1038/s41582-018-0013-z PMC6463489

[275] Muraoka, S. et al. Proteomic and biological profiling of extracellular vesicles from Alzheimer’s disease human brain tissues. *Alzheimers Dement.* **16**, 896–907 (2020). 32301581 10.1002/alz.12089 PMC7293582

[276] Zhao, X. et al. A microtubule stabilizer ameliorates protein pathogenesis and neurodegeneration in mouse models of repetitive traumatic brain injury. *Sci. Transl. Med.* **15**, eabo6889 (2023). 37703352 10.1126/scitranslmed.abo6889 PMC10787216

[277] Hadar, A. et al. Introducing ADNP and SIRT1 as new partners regulating microtubules and histone methylation. *Mol. Psychiatry* **26**, 6550–6561 (2021). 33967268 10.1038/s41380-021-01143-9

[278] Giovinazzo, D. et al. Hydrogen sulfide is neuroprotective in Alzheimer’s disease by sulfhydrating GSK3beta and inhibiting Tau hyperphosphorylation. *Proc. Natl Acad. Sci. USA*. **118**, e2017225118 (2021). 10.1073/pnas.2017225118 PMC7848711 33431651

[279] Linnebank, M. et al. S-adenosylmethionine is decreased in the cerebrospinal fluid of patients with Alzheimer’s disease. *Neurodegener. Dis.* **7**, 373–378 (2010). 20523031 10.1159/000309657

[280] Morrison, L. D., Smith, D. D. & Kish, S. J. Brain S-adenosylmethionine levels are severely decreased in Alzheimer’s disease. *J. Neurochem.* **67**, 1328–1331 (1996). 8752143 10.1046/j.1471-4159.1996.67031328.x

[281] Chen, H. et al. Folic acid supplementation mitigates alzheimer’s disease by reducing inflammation: a randomized controlled trial. *Mediators Inflamm.* **2016**, 5912146 (2016). 27340344 10.1155/2016/5912146 PMC4909909

[282] Zhu, B., Yin, D., Zhao, H. & Zhang, L. The immunology of Parkinson’s disease. *Semin Immunopathol.* **44**, 659–672 (2022). 35674826 10.1007/s00281-022-00947-3 PMC9519672

[283] Kikuchi, T. et al. Human iPS cell-derived dopaminergic neurons function in a primate Parkinson’s disease model. *Nature* **548**, 592–596 (2017). 28858313 10.1038/nature23664

[284] Zheng, X. et al. Human iPSC-derived midbrain organoids functionally integrate into striatum circuits and restore motor function in a mouse model of Parkinson’s disease. *Theranostics* **13**, 2673–2692 (2023). 37215566 10.7150/thno.80271 PMC10196819

[285] Liu, Y. et al. Involvement of microRNA-135a-5p in the protective effects of hydrogen sulfide against Parkinson’s Disease. *Cell Physiol. Biochem.* **40**, 18–26 (2016). 27842305 10.1159/000452521

[286] Kida, K. et al. Inhaled hydrogen sulfide prevents neurodegeneration and movement disorder in a mouse model of Parkinson’s disease. *Antioxid. Redox Signal* **15**, 343–352 (2011). 21050138 10.1089/ars.2010.3671 PMC3118610

[287] A novel gene containing a trinucleotide repeat that is expanded and unstable on Huntington’s disease chromosomes. The huntington’s disease collaborative research group. *Cell* **72**, 971–983 (1993). 8458085 10.1016/0092-8674(93)90585-e

[288] Zsindely, N., Siagi, F. & Bodai, L. DNA methylation in huntington’s diseas. *Int. J. Mol. Sci*. **22**, 12736 (2021). 10.3390/ijms222312736 PMC8657460 34884540

[289] Cha, J. H. Transcriptional signatures in Huntington’s disease. *Prog. Neurobiol.* **83**, 228–248 (2007). 17467140 10.1016/j.pneurobio.2007.03.004 PMC2449822

[290] Sbodio, J. I., Snyder, S. H. & Paul, B. D. Transcriptional control of amino acid homeostasis is disrupted in Huntington’s disease. *Proc. Natl Acad. Sci. USA* **113**, 8843–8848 (2016). 27436896 10.1073/pnas.1608264113 PMC4978294

[291] Paul, B. D. & Snyder, S. H. Impaired redox signaling in Huntington’s disease: therapeutic implications. *Front Mol. Neurosci.* **12**, 68 (2019). 30941013 10.3389/fnmol.2019.00068 PMC6433839

[292] Perluigi, M. et al. Proteomic analysis of protein expression and oxidative modification in r6/2 transgenic mice: a model of Huntington disease. *Mol. Cell Proteom.* **4**, 1849–1861 (2005). 10.1074/mcp.M500090-MCP200 15968004

[293] Maiuri, T. et al. DNA repair in Huntington’s disease and spinocerebellar ataxias: somatic instability and alternative hypotheses. *J. Huntingt. Dis.* **10**, 165–173 (2021). 10.3233/JHD-200414 PMC7990435 33579859

[294] Oh, Y. M. et al. Age-related Huntington’s disease progression modeled in directly reprogrammed patient-derived striatal neurons highlights impaired autophagy. *Nat. Neurosci.* **25**, 1420–1433 (2022). 36303071 10.1038/s41593-022-01185-4 PMC10162007

[295] Jones, L., Wheeler, V. C. & Pearson, C. E. Special issue: DNA repair and somatic repeat expansion in Huntington’s disease. *J. Huntingt. Dis.* **10**, 3–5 (2021). 10.3233/JHD-219001 PMC7990429 33554921

[296] Tabassum, R., Jeong, N. Y. & Jung, J. Therapeutic importance of hydrogen sulfide in age-associated neurodegenerative diseases. *Neural Regen. Res.* **15**, 653–662 (2020). 31638087 10.4103/1673-5374.266911 PMC6975154

[297] Paul, B. D. et al. Cystathionine gamma-lyase deficiency mediates neurodegeneration in Huntington’s disease. *Nature* **509**, 96–100 (2014). 24670645 10.1038/nature13136 PMC4349202

[298] Andrich, J. et al. Hyperhomocysteinaemia in treated patients with Huntington’s disease homocysteine in HD. *Mov. Disord.* **19**, 226–228 (2004). 14978683 10.1002/mds.10629

[299] Zoccolella, S. et al. Elevated plasma homocysteine levels in patients with amyotrophic lateral sclerosis. *Neurology* **70**, 222–225 (2008). 18195267 10.1212/01.wnl.0000297193.53986.6f

[300] Hardiman, O. et al. Amyotrophic lateral sclerosis. *Nat. Rev. Dis. Prim.* **3**, 17085 (2017). 29052611 10.1038/nrdp.2017.85

[301] Tabassum, R. & Jeong, N. Y. Potential for therapeutic use of hydrogen sulfide in oxidative stress-induced neurodegenerative diseases. *Int J. Med. Sci.* **16**, 1386–1396 (2019). 31692944 10.7150/ijms.36516 PMC6818192

[302] Suchy, J., Lee, S., Ahmed, A. & Shea, T. B. Dietary supplementation with S-adenosyl methionine delays the onset of motor neuron pathology in a murine model of amyotrophic lateral sclerosis. *Neuromol. Med.* **12**, 86–97 (2010). 10.1007/s12017-009-8089-7 19757209

[303] Yamout, B. I. & Alroughani, R. Multiple Sclerosis. *Semin Neurol.* **38**, 212–225 (2018). 29791948 10.1055/s-0038-1649502

[304] Tollar, J. et al. Exercise effects on multiple sclerosis quality of life and clinical-motor symptoms. *Med Sci. Sports Exerc.* **52**, 1007–1014 (2020). 31876670 10.1249/MSS.0000000000002228

[305] Karussis, D. The diagnosis of multiple sclerosis and the various related demyelinating syndromes: a critical review. *J. Autoimmun.* **48–49**, 134–142 (2014). 24524923 10.1016/j.jaut.2014.01.022

[306] Bystricka, Z., Laubertova, L., Durfinova, M. & Paduchova, Z. Methionine metabolism and multiple sclerosis. *Biomarkers* **22**, 747–754 (2017). 28562101 10.1080/1354750X.2017.1334153

[307] Russo, C. et al. Hyperhomocysteinemia is associated with cognitive impairment in multiple sclerosis. *J. Neurol.* **255**, 64–69 (2008). 18080853 10.1007/s00415-007-0668-7

[308] Sahin, S. et al. Increased plasma homocysteine levels in multiple sclerosis. *Mult. Scler.* **13**, 945–946 (2007). 17881404 10.1177/1352458506075503

[309] Ghaiad, H. R. et al. Modulating miR-146a expression by hydrogen sulfide ameliorates motor dysfunction and axonal demyelination in cuprizone-induced multiple sclerosis. *ACS Chem. Neurosci.* **14**, 3047–3058 (2023). 37585620 10.1021/acschemneuro.3c00141

[310] Lazarevic, M. et al. Upregulation of tolerogenic pathways by the hydrogen sulfide donor GYY4137 and impaired expression of H(2)S-producing enzymes in multiple sclerosis. *Antioxidants (Basel)*. **9**, 608 (2020). 10.3390/antiox9070608 PMC7402185 32664399

[311] Li, M., Mao, J. C. & Zhu, Y. Z. Hydrogen sulfide: a novel immunoinflammatory regulator in rheumatoid arthritis. *Adv. Exp. Med. Biol.* **1315**, 161–179 (2021). 34302692 10.1007/978-981-16-0991-6_7

[312] Seki, T. et al. d-Cysteine promotes dendritic development in primary cultured cerebellar Purkinje cells via hydrogen sulfide production. *Mol. Cell Neurosci.* **93**, 36–47 (2018). 30342995 10.1016/j.mcn.2018.10.002

[313] Zhu, X. Y., Gu, H. & Ni, X. Hydrogen sulfide in the endocrine and reproductive systems. *Expert Rev. Clin. Pharm.* **4**, 75–82 (2011). 10.1586/ecp.10.125 22115350

[314] Hine, C., Zhu, Y., Hollenberg, A. N. & Mitchell, J. R. Dietary and endocrine regulation of endogenous hydrogen sulfide production: implications for longevity. *Antioxid. Redox. Signal* **28**, 1483–1502 (2018). 29634343 10.1089/ars.2017.7434 PMC5930795

[315] Wu, L. et al. Pancreatic islet overproduction of H2S and suppressed insulin release in Zucker diabetic rats. *Lab. Invest.* **89**, 59–67 (2009). 19002107 10.1038/labinvest.2008.109

[316] Bao, L., Vlcek, C., Paces, V. & Kraus, J. P. Identification and tissue distribution of human cystathionine beta-synthase mRNA isoforms. *Arch. Biochem. Biophys.* **350**, 95–103 (1998). 9466825 10.1006/abbi.1997.0486

[317] Nagahara, N. Multiple role of 3-mercaptopyruvate sulfurtransferase: antioxidative function, H(2) S and polysulfide production and possible SO(x) production. *Br. J. Pharm.* **175**, 577–589 (2018). 10.1111/bph.14100 PMC5786452 29156095

[318] Suzuki, K. et al. Clinical implication of plasma hydrogen sulfide levels in japanese patients with type 2 diabetes. *Intern. Med.* **56**, 17–21 (2017). 28049995 10.2169/internalmedicine.56.7403 PMC5313420

[319] Dutta, M. et al. Evaluation of plasma H2S levels and H2S synthesis in streptozotocin induced Type-2 diabetes-an experimental study based on Swietenia macrophylla seeds. *Asian Pac. J. Trop. Biomed.* **4**, S483–S487 (2014). 25183134 10.12980/APJTB.4.201414B58 PMC4025317

[320] Zhou, X., An, G. & Lu, X. Hydrogen sulfide attenuates the development of diabetic cardiomyopathy. *Clin. Sci. (Lond.)* **128**, 325–335 (2015). 25394291 10.1042/CS20140460

[321] Mursleen, M. T. & Riaz, S. Implication of homocysteine in diabetes and impact of folate and vitamin B12 in diabetic population. *Diab. Metab. Syndr.* **11**, S141–S146 (2017). 10.1016/j.dsx.2016.12.023 28024829

[322] Fotiou, P. et al. Vitamin status as a determinant of serum homocysteine concentration in type 2 diabetic retinopathy. *J. Diab. Res.* **2014**, 807209 (2014). 10.1155/2014/807209 PMC4071945 25006590

[323] Luo, W. M. et al. The association of homocysteine and diabetic retinopathy in homocysteine cycle in chinese patients with type 2 diabetes. *Front Endocrinol. (Lausanne)* **13**, 883845 (2022). 35846275 10.3389/fendo.2022.883845 PMC9276920

[324] Tuttle, K. R. et al. Molecular mechanisms and therapeutic targets for diabetic kidney disease. *Kidney Int.* **102**, 248–260 (2022). 35661785 10.1016/j.kint.2022.05.012

[325] Barrera-Chimal, J., Lima-Posada, I., Bakris, G. L. & Jaisser, F. Mineralocorticoid receptor antagonists in diabetic kidney disease - mechanistic and therapeutic effects. *Nat. Rev. Nephrol.* **18**, 56–70 (2022). 34675379 10.1038/s41581-021-00490-8

[326] Pereira, P. R. et al. Metabolomics as a tool for the early diagnosis and prognosis of diabetic kidney disease. *Med. Res. Rev.* **42**, 1518–1544 (2022). 35274315 10.1002/med.21883

[327] Poirier, L. A. et al. Blood S-adenosylmethionine concentrations and lymphocyte methylenetetrahydrofolate reductase activity in diabetes mellitus and diabetic nephropathy. *Metabolism* **50**, 1014–1018 (2001). 11555831 10.1053/meta.2001.25655

[328] Liu, X. Q. et al. Carnosine alleviates diabetic nephropathy by targeting GNMT, a key enzyme mediating renal inflammation and fibrosis. *Clin. Sci. (Lond.)* **134**, 3175–3193 (2020). 33241846 10.1042/CS20201207 PMC7726623

[329] Hussain Lodhi, A., Ahmad, F. U., Furwa, K. & Madni, A. Role of oxidative stress and reduced endogenous hydrogen sulfide in diabetic nephropathy. *Drug Des. Devel Ther.* **15**, 1031–1043 (2021). 33707940 10.2147/DDDT.S291591 PMC7943325

[330] Kitada, M. et al. Sirtuins and renal diseases: relationship with aging and diabetic nephropathy. *Clin. Sci. (Lond.)* **124**, 153–164 (2013). 23075334 10.1042/CS20120190 PMC3466784

[331] He, W. et al. Sirt1 activation protects the mouse renal medulla from oxidative injury. *J. Clin. Invest.* **120**, 1056–1068 (2010). 20335659 10.1172/JCI41563 PMC2846063

[332] Liang, J., Tian, S., Han, J. & Xiong, P. Resveratrol as a therapeutic agent for renal fibrosis induced by unilateral ureteral obstruction. *Ren. Fail* **36**, 285–291 (2014). 24152192 10.3109/0886022X.2013.844644

[333] Ahmed, H. H. et al. Hydrogen sulfide modulates SIRT1 and suppresses oxidative stress in diabetic nephropathy. *Mol. Cell Biochem.* **457**, 1–9 (2019). 30778838 10.1007/s11010-019-03506-x

[334] Iseki, K. Parathyroid hormone and the vascular response to norepinephrine. *Am. J. Hypertens.* **3**, 238S–240S (1990). 2222975 10.1093/ajh/3.8.238

[335] Caballero, B. Humans against obesity: who will win? *Adv. Nutr.* **10**, S4–S9 (2019). 30721956 10.1093/advances/nmy055 PMC6363526

[336] Pulgaron, E. R. & Delamater, A. M. Obesity and type 2 diabetes in children: epidemiology and treatment. *Curr. Diab Rep.* **14**, 508 (2014). 24919749 10.1007/s11892-014-0508-y PMC4099943

[337] Malone, J. I. & Hansen, B. C. Does obesity cause type 2 diabetes mellitus (T2DM)? Or is it the opposite? *Pediatr. Diab.* **20**, 5–9 (2019). 10.1111/pedi.12787 30311716

[338] Elshorbagy, A. K., Smith, A. D., Kozich, V. & Refsum, H. Cysteine and obesity. *Obes. (Silver Spring)* **20**, 473–481 (2012). 10.1038/oby.2011.93 21546934

[339] Elshorbagy, A. K. et al. Cysteine supplementation reverses methionine restriction effects on rat adiposity: significance of stearoyl-coenzyme A desaturase. *J. Lipid Res.* **52**, 104–112 (2011). 20871132 10.1194/jlr.M010215 PMC2999932

[340] McGavigan, A. K. et al. L-cysteine suppresses ghrelin and reduces appetite in rodents and humans. *Int. J. Obes. (Lond.)* **39**, 447–455 (2015). 25219528 10.1038/ijo.2014.172 PMC4276721

[341] Whiteman, M. et al. Adiposity is a major determinant of plasma levels of the novel vasodilator hydrogen sulphide. *Diabetologia* **53**, 1722–1726 (2010). 20414636 10.1007/s00125-010-1761-5

[342] Tsai, C. Y. et al. Hydrogen sulfide promotes adipogenesis in 3T3L1 cells. *PLoS ONE* **10**, e0119511 (2015). 25822632 10.1371/journal.pone.0119511 PMC4378953

[343] Mani, S., Yang, G. & Wang, R. A critical life-supporting role for cystathionine gamma-lyase in the absence of dietary cysteine supply. *Free Radic. Biol. Med.* **50**, 1280–1287 (2011). 21310231 10.1016/j.freeradbiomed.2011.01.038

[344] Gupta, S. & Kruger, W. D. Cystathionine beta-synthase deficiency causes fat loss in mice. *PLoS One* **6**, e27598 (2011). 22096601 10.1371/journal.pone.0027598 PMC3214081

[345] Geng, B. et al. Increase or decrease hydrogen sulfide exert opposite lipolysis, but reduce global insulin resistance in high fatty diet induced obese mice. *PLoS ONE* **8**, e73892 (2013). 24058499 10.1371/journal.pone.0073892 PMC3772803

[346] Beltowski, J. & Jamroz-Wisniewska, A. Hydrogen sulfide in the adipose tissue-physiology, pathology and a target for pharmacotherapy. *Molecules*. **22**, 63 (2016). 10.3390/molecules22010063 PMC6155731 28042862

[347] Nohara, T. et al. Antitumor allium sulfides. *Chem. Pharm. Bull. (Tokyo)* **65**, 209–217 (2017). 28250342 10.1248/cpb.c16-00844

[348] Khattak, S. et al. Hydrogen sulfide biology and its role in cancer. *Molecules*. **27**, 3389 (2022). 10.3390/molecules27113389 PMC9181954 35684331

[349] Majumder, A. Targeting homocysteine and hydrogen sulfide balance as future therapeutics in cancer treatment. *Antioxidants (Basel)*. **12**, (2023). 10.3390/antiox12081520 PMC10451792 37627515

[350] Santos, S. S. et al. Role of cystathionine beta-synthase and 3-mercaptopyruvate Sulfurtransferase in the regulation of proliferation, migration, and bioenergetics of murine breast cancer cells. *Antioxidants (Basel)*. **12**, 1520 (2023). 10.3390/antiox12030647 PMC10045476 36978895

[351] Fu, M. et al. Hydrogen sulfide (H2S) metabolism in mitochondria and its regulatory role in energy production. *Proc. Natl Acad. Sci. USA* **109**, 2943–2948 (2012). 22323590 10.1073/pnas.1115634109 PMC3287003

[352] Blanc, V. et al. Apobec1 complementation factor overexpression promotes hepatic steatosis, fibrosis, and hepatocellular cancer. *J. Clin. Invest*. **131**, e138699 (2021). 10.1172/JCI138699 PMC7773377 33445170

[353] Zhou, T. et al. DTYMK promote hepatocellular carcinoma proliferation by regulating cell cycle. *Cell Cycle* **20**, 1681–1691 (2021). 34369850 10.1080/15384101.2021.1958502 PMC8489954

[354] Zhu, H. et al. CCAT1 promotes hepatocellular carcinoma cell proliferation and invasion. *Int. J. Clin. Exp. Pathol.* **8**, 5427–5434 (2015). 26191246 PMC4503117

[355] Wu, D. et al. Hydrogen sulfide acts as a double-edged sword in human hepatocellular carcinoma cells through EGFR/ERK/MMP-2 and PTEN/AKT signaling pathways. *Sci. Rep.* **7**, 5134 (2017). 28698660 10.1038/s41598-017-05457-z PMC5506015

[356] Wang, S. S. et al. Hydrogen sulfide promotes autophagy of hepatocellular carcinoma cells through the PI3K/Akt/mTOR signaling pathway. *Cell Death Dis.* **8**, e2688 (2017). 28333142 10.1038/cddis.2017.18 PMC5386547

[357] Zhen, Y. et al. Exogenous hydrogen sulfide promotes hepatocellular carcinoma cell growth by activating the STAT3-COX-2 signaling pathway. *Oncol. Lett.* **15**, 6562–6570 (2018). 29725404 10.3892/ol.2018.8154 PMC5920354

[358] Lozano-Rosas, M. G. et al. Diminished S-adenosylmethionine biosynthesis and its metabolism in a model of hepatocellular carcinoma is recuperated by an adenosine derivative. *Cancer Biol. Ther.* **21**, 81–94 (2020). 31552788 10.1080/15384047.2019.1665954 PMC7012146

[359] Gou, D. et al. Gluconeogenic enzyme PCK1 supports S-adenosylmethionine biosynthesis and promotes H3K9me3 modification to suppress hepatocellular carcinoma progression. *J. Clin. Invest*. **133**, e161713 (2023). 10.1172/JCI161713 PMC10313362 37166978

[360] Wang, T. et al. Disulfidptosis classification of hepatocellular carcinoma reveals correlation with clinical prognosis and immune profile. *Int. Immunopharmacol.* **120**, 110368 (2023). 37247499 10.1016/j.intimp.2023.110368

[361] Mahmood, N. et al. S-adenosylmethionine in combination with decitabine shows enhanced anti-cancer effects in repressing breast cancer growth and metastasis. *J. Cell Mol. Med.* **24**, 10322–10337 (2020). 32720467 10.1111/jcmm.15642 PMC7521255

[362] Cave, D. D. et al. S-Adenosylmethionine-mediated apoptosis is potentiated by autophagy inhibition induced by chloroquine in human breast cancer cells. *J. Cell Physiol.* **233**, 1370–1383 (2018). 28518408 10.1002/jcp.26015

[363] Akilzhanova, A. et al. Genetic profile and determinants of homocysteine levels in Kazakhstan patients with breast cancer. *Anticancer Res.* **33**, 4049–4059 (2013). 24023349

[364] Lin, J. et al. Plasma homocysteine and cysteine and risk of breast cancer in women. *Cancer Res* **70**, 2397–2405 (2010). 20197471 10.1158/0008-5472.CAN-09-3648 PMC2840179

[365] Khan, N. H. et al. pharmacological inhibition of endogenous hydrogen sulfide attenuates breast cancer progression. *Molecules*. **27**, 4049 (2022). 10.3390/molecules27134049 PMC9268373 35807290

[366] Deepak, K. G. K. et al. Tumor microenvironment: challenges and opportunities in targeting metastasis of triple negative breast cancer. *Pharm. Res.* **153**, 104683 (2020). 10.1016/j.phrs.2020.104683 32050092

[367] Lev, S. Targeted therapy and drug resistance in triple-negative breast cancer: the EGFR axis. *Biochem. Soc. Trans.* **48**, 657–665 (2020). 32311020 10.1042/BST20191055

[368] Leon-Ferre, R. A. & Goetz, M. P. Advances in systemic therapies for triple negative breast cancer. *BMJ* **381**, e071674 (2023). 37253507 10.1136/bmj-2022-071674

[369] Liu, Y. et al. A novel cystathionine gamma-lyase inhibitor, I194496, inhibits the growth and metastasis of human TNBC via downregulating multiple signaling pathways. *Sci. Rep.* **11**, 8963 (2021). 33903672 10.1038/s41598-021-88355-9 PMC8076300

[370] Liu, Y. et al. Antimetastatic therapies of the polysulfide diallyl trisulfide against triple-negative breast cancer (TNBC) via suppressing MMP2/9 by blocking NF-kappaB and ERK/MAPK signaling pathways. *PLoS ONE* **10**, e0123781 (2015). 25927362 10.1371/journal.pone.0123781 PMC4415928

[371] Huang, J. et al. Diallyl disulfide inhibits growth and metastatic potential of human triple-negative breast cancer cells through inactivation of the beta-catenin signaling pathway. *Mol. Nutr. Food Res.* **59**, 1063–1075 (2015). 25755089 10.1002/mnfr.201400668

[372] Ferroni, P. et al. Determinants of homocysteine levels in colorectal and breast cancer patients. *Anticancer Res* **29**, 4131–4138 (2009). 19846961

[373] Akoglu, B., Milovic, V., Caspary, W. F. & Faust, D. Hyperproliferation of homocysteine-treated colon cancer cells is reversed by folate and 5-methyltetrahydrofolate. *Eur. J. Nutr.* **43**, 93–99 (2004). 15083316 10.1007/s00394-004-0446-6

[374] Wang, L. et al. PCSK9 promotes the progression and metastasis of colon cancer cells through regulation of EMT and PI3K/AKT signaling in tumor cells and phenotypic polarization of macrophages. *J. Exp. Clin. Cancer Res.* **41**, 303 (2022). 36242053 10.1186/s13046-022-02477-0 PMC9563506

[375] Stehr, A. M. et al. Neutrophil extracellular traps drive epithelial-mesenchymal transition of human colon cancer. *J. Pathol.* **256**, 455–467 (2022). 34939675 10.1002/path.5860

[376] Mao, L. et al. Decorin deficiency promotes epithelial-mesenchymal transition and colon cancer metastasis. *Matrix Biol.* **95**, 1–14 (2021). 33065248 10.1016/j.matbio.2020.10.001 PMC7870527

[377] Sun, L. et al. Wnt/beta-catenin signalling, epithelial-mesenchymal transition and crosslink signalling in colorectal cancer cells. *Biomed. Pharmacother.* **175**, 116685 (2024). 38710151 10.1016/j.biopha.2024.116685

[378] Peng, C. et al. Norcantharidin suppresses colon cancer cell epithelial-mesenchymal transition by inhibiting the alphavbeta6-ERK-Ets1 signaling pathway. *Sci. Rep.* **6**, 20500 (2016). 26846153 10.1038/srep20500 PMC4742802

[379] Pavlic, A. et al. Epithelial-mesenchymal transition in colorectal carcinoma: comparison between primary tumor, lymph node and liver metastases. *Front Oncol.* **11**, 662806 (2021). 34046357 10.3389/fonc.2021.662806 PMC8144630

[380] Ascencao, K. et al. Pharmacological induction of mesenchymal-epithelial transition via inhibition of H2S biosynthesis and consequent suppression of ACLY activity in colon cancer cells. *Pharm. Res.* **165**, 105393 (2021). 10.1016/j.phrs.2020.105393 33484818

[381] Wen, J. et al. ACLY facilitates colon cancer cell metastasis by CTNNB1. *J. Exp. Clin. Cancer Res.* **38**, 401 (2019). 31511060 10.1186/s13046-019-1391-9 PMC6740040

[382] Hu, G. et al. A bioinformatics approach to identify a disulfidptosis-related gene signature for prognostic implication in colon adenocarcinoma. *Sci. Rep.* **13**, 12403 (2023). 37524774 10.1038/s41598-023-39563-y PMC10390519

[383] Untereiner, A. A. et al. H(2)S-induced S-sulfhydration of lactate dehydrogenase a (LDHA) stimulates cellular bioenergetics in HCT116 colon cancer cells. *Biochem. Pharm.* **136**, 86–98 (2017). 28404377 10.1016/j.bcp.2017.03.025 PMC5494970

[384] Zhang, Y. et al. Overexpression of CBS/H(2)S inhibits proliferation and metastasis of colon cancer cells through downregulation of CD44. *Cancer Cell Int.* **22**, 85 (2022). 35172821 10.1186/s12935-022-02512-2 PMC8848668

[385] Hellmich, M. R., Coletta, C., Chao, C. & Szabo, C. The therapeutic potential of cystathionine beta-synthetase/hydrogen sulfide inhibition in cancer. *Antioxid. Redox Signal* **22**, 424–448 (2015). 24730679 10.1089/ars.2014.5933 PMC4307161

[386] Zheng, M. Classification and pathology of lung cancer. *Surg. Oncol. Clin. N. Am.* **25**, 447–468 (2016). 27261908 10.1016/j.soc.2016.02.003

[387] Ruiz-Cordero, R. & Devine, W. P. Targeted therapy and checkpoint immunotherapy in lung cancer. *Surg. Pathol. Clin.* **13**, 17–33 (2020). 32005431 10.1016/j.path.2019.11.002

[388] Vokes, N. I. et al. Concurrent TP53 mutations facilitate resistance evolution in EGFR-mutant lung adenocarcinoma. *J. Thorac. Oncol.* **17**, 779–792 (2022). 35331964 10.1016/j.jtho.2022.02.011 PMC10478031

[389] Huang, J. et al. Identification of a disulfidptosis-related genes signature for prognostic implication in lung adenocarcinoma. *Comput Biol. Med.* **165**, 107402 (2023). 37657358 10.1016/j.compbiomed.2023.107402

[390] Greenberg, A. K. et al. S-adenosylmethionine as a biomarker for the early detection of lung cancer. *Chest* **132**, 1247–1252 (2007). 17934114 10.1378/chest.07-0622 PMC2562751

[391] Wang, M. et al. Hydrogen sulfide modulates epithelial-mesenchymal transition and angiogenesis in non-small cell lung cancer via HIF-1alpha activation. *Biochem. Pharm.* **172**, 113775 (2020). 31870768 10.1016/j.bcp.2019.113775

[392] Vincent, A. et al. Pancreatic cancer. *Lancet* **378**, 607–620 (2011). 21620466 10.1016/S0140-6736(10)62307-0 PMC3062508

[393] Ren, B. et al. Tumor microenvironment participates in metastasis of pancreatic cancer. *Mol. Cancer* **17**, 108 (2018). 30060755 10.1186/s12943-018-0858-1 PMC6065152

[394] Wang, S., You, L., Dai, M. & Zhao, Y. Mucins in pancreatic cancer: a well-established but promising family for diagnosis, prognosis and therapy. *J. Cell Mol. Med.* **24**, 10279–10289 (2020). 32745356 10.1111/jcmm.15684 PMC7521221

[395] Citi, V. et al. Anticancer properties of erucin, an H(2) S-releasing isothiocyanate, on human pancreatic adenocarcinoma cells (AsPC-1). *Phytother. Res.* **33**, 845–855 (2019). 30632211 10.1002/ptr.6278

[396] Chen, X. et al. Activation of Nrf2 by sulforaphane inhibits high glucose-induced progression of pancreatic cancer via AMPK dependent signaling. *Cell Physiol. Biochem.* **50**, 1201–1215 (2018). 30355942 10.1159/000494547

[397] Montenegro, M. F. et al. Targeting protein methylation in pancreatic cancer cells results in KRAS signaling imbalance and inhibition of autophagy. *Cell Death Dis.* **14**, 761 (2023). 37996408 10.1038/s41419-023-06288-9 PMC10667277

[398] Yang, P. W. et al. Keep a watchful eye on methionine adenosyltransferases, novel therapeutic opportunities for hepatobiliary and pancreatic tumours. *Biochim. Biophys. Acta Rev. Cancer* **1877**, 188793 (2022). 36089205 10.1016/j.bbcan.2022.188793

[399] Kossai, M., Leary, A., Scoazec, J. Y. & Genestie, C. Ovarian cancer: a heterogeneous disease. *Pathobiology* **85**, 41–49 (2018). 29020678 10.1159/000479006

[400] O’Shea, A. S. Clinical staging of ovarian cancer. *Methods Mol. Biol.* **2424**, 3–10 (2022). 34918284 10.1007/978-1-0716-1956-8_1

[401] Jiang, Y., Wang, C. & Zhou, S. Targeting tumor microenvironment in ovarian cancer: Premise and promise. *Biochim Biophys. Acta Rev. Cancer* **1873**, 188361 (2020). 32234508 10.1016/j.bbcan.2020.188361

[402] Jin, M., Ni, D., Cai, J. & Yang, J. Identification and validation of immunity- and disulfidptosis-related genes signature for predicting prognosis in ovarian cancer. *Heliyon* **10**, e32273 (2024). 38952356 10.1016/j.heliyon.2024.e32273 PMC11215265

[403] Zhu, H. et al. Cystathionine beta-synthase in physiology and cancer. *Biomed. Res. Int.* **2018**, 3205125 (2018). 30050925 10.1155/2018/3205125 PMC6046153

[404] Bhattacharyya, S. et al. Cystathionine beta-synthase (CBS) contributes to advanced ovarian cancer progression and drug resistance. *PLoS ONE* **8**, e79167 (2013). 24236104 10.1371/journal.pone.0079167 PMC3827285

[405] Chakraborty, P. K. et al. Role of cystathionine beta synthase in lipid metabolism in ovarian cancer. *Oncotarget* **6**, 37367–37384 (2015). 26452259 10.18632/oncotarget.5424 PMC4741935

[406] Siegel, R. L., Miller, K. D. & Jemal, A. Cancer statistics, 2015. *CA. Cancer J. Clin.* **65**, 5–29 (2015). 25559415 10.3322/caac.21254

[407] Litwin, M. S. & Tan, H. J. The diagnosis and treatment of prostate cancer: a review. *JAMA* **317**, 2532–2542 (2017). 28655021 10.1001/jama.2017.7248

[408] Preisser, F. et al. Intermediate-risk prostate cancer: stratification and management. *Eur. Urol. Oncol.* **3**, 270–280 (2020). 32303478 10.1016/j.euo.2020.03.002

[409] Wang, Y. H. et al. Dysregulation of cystathionine gamma-lyase promotes prostate cancer progression and metastasis. *EMBO Rep.* **20**, e45986 (2019). 31468690 10.15252/embr.201845986 PMC6776913

[410] Schmidt, T. S-Adenosylmethionine affects ERK1/2 and STAT3 pathway in androgen-independent prostate cancer cells. *Mol. Biol. Rep.* **49**, 4805–4817 (2022). 35303200 10.1007/s11033-022-07331-2 PMC9262802

[411] Bracken, A. P. et al. EZH2 is downstream of the pRB-E2F pathway, essential for proliferation and amplified in cancer. *EMBO J.* **22**, 5323–5335 (2003). 14532106 10.1093/emboj/cdg542 PMC213796

[412] Varambally, S. et al. The polycomb group protein EZH2 is involved in progression of prostate cancer. *Nature* **419**, 624–629 (2002). 12374981 10.1038/nature01075

[413] Uchiyama, N., Tanaka, Y. & Kawamoto, T. Aristeromycin and DZNeP cause growth inhibition of prostate cancer via induction of mir-26a. *Eur. J. Pharm.* **812**, 138–146 (2017). 10.1016/j.ejphar.2017.07.023 28705714

[414] Francis, R. C. et al. Protective and detrimental effects of sodium sulfide and hydrogen sulfide in murine ventilator-induced lung injury. *Anesthesiology* **115**, 1012–1021 (2011). 21912243 10.1097/ALN.0b013e31823306cf PMC3752661

[415] Li, L. et al. Characterization of a novel, water-soluble hydrogen sulfide-releasing molecule (GYY4137): new insights into the biology of hydrogen sulfide. *Circulation* **117**, 2351–2360 (2008). 18443240 10.1161/CIRCULATIONAHA.107.753467

[416] Wang, Y. et al. Hydrogen sulfide alleviates particulate matter-induced emphysema and airway inflammation by suppressing ferroptosis. *Free Radic. Biol. Med.* **186**, 1–16 (2022). 35490984 10.1016/j.freeradbiomed.2022.04.014

[417] Wang, M. et al. Exogenous H(2)S initiating Nrf2/GPx4/GSH pathway through promoting Syvn1-Keap1 interaction in diabetic hearts. *Cell Death Discov.* **9**, 394 (2023). 37875467 10.1038/s41420-023-01690-w PMC10598017

[418] Kupai, K. et al. H(2)S confers colonoprotection against TNBS-induced colitis by HO-1 upregulation in rats. *Inflammopharmacology* **26**, 479–489 (2018). 28770475 10.1007/s10787-017-0382-8

[419] Torok, S. et al. Protective effects of H(2)S donor treatment in experimental colitis: a focus on antioxidants. *Antioxidants (Basel)*. **12**, 1025 (2023). 10.3390/antiox12051025 PMC10215296 37237891

[420] Lucetti, L. T. et al. Nitric oxide and hydrogen sulfide interact when modulating gastric physiological functions in rodents. *Dig. Dis. Sci.* **62**, 93–104 (2017). 27864656 10.1007/s10620-016-4377-x

[421] Nicolau, L. A. et al. The hydrogen sulfide donor, Lawesson’s reagent, prevents alendronate-induced gastric damage in rats. *Braz. J. Med Biol. Res.* **46**, 708–714 (2013). 23969974 10.1590/1414-431X20133030 PMC3854416

[422] Star, B. S. et al. GYY4137-derived hydrogen sulfide donates electrons to the mitochondrial electron transport chain via sulfide: quinone oxidoreductase in endothelial cells. *Antioxidants (Basel)*. **12**, 587 (2023). 10.3390/antiox12030587 PMC10044827 36978834

[423] Hasegawa, U. & van der Vlies, A. J. Design and synthesis of polymeric hydrogen sulfide donors. *Bioconjug. Chem.* **25**, 1290–1300 (2014). 24942989 10.1021/bc500150s

[424] Cai, F. F. et al. ADT-OH inhibits malignant melanoma metastasis in mice via suppressing CSE/CBS and FAK/Paxillin signaling pathway. *Acta Pharm. Sin.* **43**, 1829–1842 (2022). 10.1038/s41401-021-00799-x PMC9253130 34795411

[425] Dong, Q. et al. A novel hydrogen sulfide-releasing donor, HA-ADT, suppresses the growth of human breast cancer cells through inhibiting the PI3K/AKT/mTOR and Ras/Raf/MEK/ERK signaling pathways. *Cancer Lett.* **455**, 60–72 (2019). 31042588 10.1016/j.canlet.2019.04.031

[426] Montanaro, R. et al. Hydrogen sulfide donor AP123 restores endothelial nitric oxide-dependent vascular function in hyperglycemia via a CREB-dependent pathway. *Redox Biol.* **62**, 102657 (2023). 36913800 10.1016/j.redox.2023.102657 PMC10025109

[427] Lohakul, J. et al. Mitochondria-targeted hydrogen sulfide delivery molecules protect against UVA-induced photoaging in human dermal fibroblasts, and in mouse skin in vivo. *Antioxid. Redox Signal* **36**, 1268–1288 (2022). 34235951 10.1089/ars.2020.8255

[428] De Cicco, P. et al. ATB-346, a novel hydrogen sulfide-releasing anti-inflammatory drug, induces apoptosis of human melanoma cells and inhibits melanoma development in vivo. *Pharm. Res.* **114**, 67–73 (2016). 10.1016/j.phrs.2016.10.019 27777130

[429] Van Dingenen, J., Pieters, L., Vral, A. & Lefebvre, R. A. The H(2)S-releasing naproxen derivative ATB-346 and the slow-release H(2)S donor GYY4137 reduce intestinal inflammation and restore transit in postoperative ileuss. *Front Pharm.* **10**, 116 (2019). 10.3389/fphar.2019.00116 PMC6391894 30842737

[430] Dzielska-Olczak, M. Cyclooxygenases inhibitors and other compounds with antiinflammatory potential in osteoarthrosis–part II. *Pol. Merkur Lekarski* **30**, 82–86 (2011). 21542252

[431] Amagase, H. Clarifying the real bioactive constituents of garlic. *J. Nutr.* **136**, 716S–725S (2006). 16484550 10.1093/jn/136.3.716S

[432] Benavides, G. A. et al. Hydrogen sulfide mediates the vasoactivity of garlic. *Proc. Natl Acad. Sci. USA* **104**, 17977–17982 (2007). 17951430 10.1073/pnas.0705710104 PMC2084282

[433] Jeremic, J. N. et al. Garlic derived diallyl trisulfide in experimental metabolic syndrome: metabolic effects and cardioprotective Role. *Int. J. Mol. Sci*. **21**, 9100 (2020). 10.3390/ijms21239100 PMC7730157 33265949

[434] Gojon, G. & Morales, G. A. SG1002 and catenated divalent organic sulfur compounds as promising hydrogen sulfide prodrugs. *Antioxid. Redox Signal* **33**, 1010–1045 (2020). 32370538 10.1089/ars.2020.8060 PMC7578191

[435] Islam, R. K. et al. H(2)S prodrug, SG-1002, protects against myocardial oxidative damage and hypertrophy in vitro via induction of cystathionine beta-synthase and antioxidant proteins. *Biomedicines*. **11**, 612 (2023). 10.3390/biomedicines11020612 PMC9953594 36831146

[436] Polhemus, D. J. et al. A novel hydrogen sulfide prodrug, SG1002, promotes hydrogen sulfide and nitric oxide bioavailability in heart failure patients. *Cardiovasc. Ther.* **33**, 216–226 (2015). 25930144 10.1111/1755-5922.12128 PMC5034803

[437] Kondo, K. et al. H(2)S protects against pressure overload-induced heart failure via upregulation of endothelial nitric oxide synthase. *Circulation* **127**, 1116–1127 (2013). 23393010 10.1161/CIRCULATIONAHA.112.000855 PMC3670187

[438] Sonobe, T. & Haouzi, P. H2S concentrations in the heart after acute H2S administration: methodological and physiological considerations. *Am. J. Physiol. Heart Circ. Physiol.* **311**, H1445–H1458 (2016). 27638880 10.1152/ajpheart.00464.2016 PMC5206345

[439] Balan, H., Popescu, E. & Angelescu, G. Comparing different treatment schedules of Zomen (zofenopril). *Rom. J. Intern. Med.* **49**, 75–84 (2011). 22026256

[440] Macabrey, D. et al. Hydrogen sulphide release via the angiotensin converting enzyme inhibitor zofenopril prevents intimal hyperplasia in human vein segments and in a mouse model of carotid artery stenosis. *Eur. J. Vasc. Endovasc. Surg.* **63**, 336–346 (2022). 34916111 10.1016/j.ejvs.2021.09.032

[441] Donnarumma, E. et al. Zofenopril protects against myocardial ischemia-reperfusion injury by increasing nitric oxide and hydrogen sulfide bioavailability. *J. Am. Heart Assoc*. **5**, e003531 (2016). 10.1161/JAHA.116.003531 PMC5015391 27381758

[442] Pedre, B., Barayeu, U., Ezerina, D. & Dick, T. P. The mechanism of action of N-acetylcysteine (NAC): The emerging role of H(2)S and sulfane sulfur species. *Pharm. Ther.* **228**, 107916 (2021). 10.1016/j.pharmthera.2021.107916 34171332

[443] Buhimschi, C. S. et al. Antenatal N-acetylcysteine to improve outcomes of premature infants with intra-amniotic infection and inflammation (Triple I): randomized clinical trial. *Pediatr. Res.* **89**, 175–184 (2021). 32818949 10.1038/s41390-020-01106-w PMC7451831

[444] Tsikas, D. et al. S-Nitroso-N-acetyl-L-cysteine ethyl ester (SNACET) and N-acetyl-L-cysteine ethyl ester (NACET)-Cysteine-based drug candidates with unique pharmacological profiles for oral use as NO, H(2)S and GSH suppliers and as antioxidants: results and overview. *J. Pharm. Anal.* **8**, 1–9 (2018). 29568662 10.1016/j.jpha.2017.12.003 PMC5859134

[445] Foretz, M. et al. Metformin: from mechanisms of action to therapies. *Cell Metab.* **20**, 953–966 (2014). 25456737 10.1016/j.cmet.2014.09.018

[446] Ma, X., Jiang, Z., Wang, Z. & Zhang, Z. Administration of metformin alleviates atherosclerosis by promoting H2S production via regulating CSE expression. *J. Cell Physiol.* **235**, 2102–2112 (2020). 31338841 10.1002/jcp.29112

[447] Wilinski, B. et al. Metformin raises hydrogen sulfide tissue concentrations in various mouse organs. *Pharm. Rep.* **65**, 737–742 (2013). 10.1016/s1734-1140(13)71053-3 23950598

[448] Wierzbicki, A. S. Atorvastatin. *Expert Opin. Pharmacother.* **2**, 819–830 (2001). 11336625 10.1517/14656566.2.5.819

[449] Xu, Y. et al. Statins upregulate cystathionine gamma-lyase transcription and H2S generation via activating Akt signaling in macrophage. *Pharm. Res.* **87**, 18–25 (2014). 10.1016/j.phrs.2014.06.006 24951966

[450] Yan, L. et al. Atorvastatin protects against contrast-induced acute kidney injury via upregulation of endogenous hydrogen sulfide. *Ren. Fail* **42**, 270–281 (2020). 33685337 10.1080/0886022X.2020.1740098 PMC7144258

[451] Dali, M. M., Dansette, P. M., Mansuy, D. & Boucher, J. L. Comparison of various aryl-dithiolethiones and aryl-dithiolones as hydrogen sulfide donors in the presence of rat liver microsomes. *Drug Metab. Dispos.* **48**, 426–431 (2020). 32234734 10.1124/dmd.119.090274

[452] Dulac, M. et al. Mechanism of H(2)S formation from the metabolism of anetholedithiolethione and anetholedithiolone by rat liver microsomes. *Drug Metab. Dispos.* **47**, 1061–1065 (2019). 31213461 10.1124/dmd.119.087205

[453] Zhao, C. et al. Slow-release H(2)S donor anethole dithiolethione protects liver from lipotoxicity by improving fatty acid metabolism. *Front Pharm.* **11**, 549377 (2020). 10.3389/fphar.2020.549377 PMC7538629 33071780

[454] Beltowski, J. Hydrogen sulfide in pharmacology and medicine–an update. *Pharm. Rep.* **67**, 647–658 (2015). 10.1016/j.pharep.2015.01.005 25933982

[455] Liang, W. et al. Conductive hydrogen sulfide-releasing hydrogel encapsulating ADSCs for myocardial infarction treatment. *ACS Appl Mater. Interfaces* **11**, 14619–14629 (2019). 30939870 10.1021/acsami.9b01886

[456] Gero, D. et al. The novel mitochondria-targeted hydrogen sulfide (H(2)S) donors AP123 and AP39 protect against hyperglycemic injury in microvascular endothelial cells in vitro. *Pharm. Res.* **113**, 186–198 (2016). 10.1016/j.phrs.2016.08.019 PMC5113977 27565382

[457] Sadybekov, A. V. & Katritch, V. Computational approaches streamlining drug discovery. *Nature* **616**, 673–685 (2023). 37100941 10.1038/s41586-023-05905-z

[458] Macalino, S. J., Gosu, V., Hong, S. & Choi, S. Role of computer-aided drug design in modern drug discovery. *Arch. Pharm. Res.* **38**, 1686–1701 (2015). 26208641 10.1007/s12272-015-0640-5

[459] Kumar, A. & Zhang, K. Y. Hierarchical virtual screening approaches in small molecule drug discovery. *Methods* **71**, 26–37 (2015). 25072167 10.1016/j.ymeth.2014.07.007 PMC7129923

[460] Ma, X. H. et al. In-silico approaches to multi-target drug discovery : computer aided multi-target drug design, multi-target virtual screening. *Pharm. Res.* **27**, 739–749 (2010). 20221898 10.1007/s11095-010-0065-2

[461] Wang, J. & Skolnik, S. Recent advances in physicochemical and ADMET profiling in drug discovery. *Chem. Biodivers.* **6**, 1887–1899 (2009). 19937823 10.1002/cbdv.200900117

[462] Vucicevic, J., Nikolic, K. & Mitchell, J. B. O. Rational drug design of antineoplastic agents using 3D-QSAR, cheminformatic, and virtual screening approaches. *Curr. Med. Chem.* **26**, 3874–3889 (2019). 28707592 10.2174/0929867324666170712115411

[463] Pey, A. L., Majtan, T., Sanchez-Ruiz, J. M. & Kraus, J. P. Human cystathionine beta-synthase (CBS) contains two classes of binding sites for S-adenosylmethionine (SAM): complex regulation of CBS activity and stability by SAM. *Biochem. J.* **449**, 109–121 (2013). 22985361 10.1042/BJ20120731

[464] Majtan, T. et al. Active cystathionine beta-synthase can be expressed in heme-free systems in the presence of metal-substituted porphyrins or a chemical chaperone. *J. Biol. Chem.* **283**, 34588–34595 (2008). 18849566 10.1074/jbc.M805928200 PMC2596375

[465] Zuhra, K., Augsburger, F., Majtan, T. & Szabo, C. Cystathionine-beta-synthase: molecular regulation and pharmacological inhibition. *Biomolecules*. **10**, 697 (2020). 10.3390/biom10050697 PMC7277093 32365821

[466] Majtan, T., Pey, A. L. & Kraus, J. P. Kinetic stability of cystathionine beta-synthase can be modulated by structural analogs of S-adenosylmethionine: potential approach to pharmacological chaperone therapy for homocystinuria. *Biochimie* **126**, 6–13 (2016). 26805382 10.1016/j.biochi.2016.01.009

[467] Niu, W. et al. Discovery of selective cystathionine beta-synthase inhibitors by high-throughput screening with a fluorescent thiol probe. *Medchemcomm* **8**, 198–201 (2017). 30108705 10.1039/c6md00493h PMC6072345

[468] Niu, W. N., Yadav, P. K., Adamec, J. & Banerjee, R. S-glutathionylation enhances human cystathionine beta-synthase activity under oxidative stress conditions. *Antioxid. Redox Signal* **22**, 350–361 (2015). 24893130 10.1089/ars.2014.5891 PMC4307034

[469] Zhou, Y. et al. High-throughput tandem-microwell assay identifies inhibitors of the hydrogen sulfide signaling pathway. *Chem. Commun. (Camb.)* **49**, 11782–11784 (2013). 24213681 10.1039/c3cc46719h

[470] Hu, Y. et al. Discovery of a bioactive inhibitor with a new scaffold for cystathionine gamma-lyase. *J. Med. Chem.* **62**, 1677–1683 (2019). 30562026 10.1021/acs.jmedchem.8b01720

[471] Hanaoka, K. et al. Discovery and mechanistic characterization of selective inhibitors of H(2)S-producing enzyme: 3-mercaptopyruvate sulfurtransferase (3MST) targeting active-site cysteine persulfide. *Sci. Rep.* **7**, 40227 (2017). 28079151 10.1038/srep40227 PMC5228037

[472] Akahoshi, N. et al. Increased urinary 3-mercaptolactate excretion and enhanced passive systemic anaphylaxis in mice lacking mercaptopyruvate sulfurtransferase, a model of mercaptolactate-cysteine disulfiduria. *Int. J. Mol. Sci*. **21**, 818 (2020). 10.3390/ijms21030818 PMC7038117 32012740

[473] Alphey, M. S. et al. The crystal structure of Leishmania major 3-mercaptopyruvate sulfurtransferase. A three-domain architecture with a serine protease-like triad at the active site. *J. Biol. Chem.* **278**, 48219–48227 (2003). 12952945 10.1074/jbc.M307187200

[474] Kant, V. et al. In silico screening, molecular dynamic simulations, and in vitro activity of selected natural compounds as an inhibitor of Leishmania donovani 3-mercaptopyruvate sulfurtransferase. *Parasitol. Res.* **121**, 2093–2109 (2022). 35536513 10.1007/s00436-022-07532-5 PMC9085559

[475] Katane, M. et al. Identification of novel D-amino acid oxidase inhibitors by in silico screening and their functional characterization in vitro. *J. Med. Chem.* **56**, 1894–1907 (2013). 23391306 10.1021/jm3017865

[476] Katane, M. et al. Identification of novel D-aspartate oxidase inhibitors by in silico screening and their functional and structural characterization in vitro. *J. Med. Chem.* **58**, 7328–7340 (2015). 26322531 10.1021/acs.jmedchem.5b00871

[477] Terry-Lorenzo, R. T. et al. Novel human D-amino acid oxidase inhibitors stabilize an active-site lid-open conformation. *Biosci Rep*. **34**, e00133 (2014). 10.1042/BSR20140071 PMC4127593 25001371

[478] Jackson, M. R. et al. Discovery of a first-in-class inhibitor of sulfide:quinone oxidoreductase that protects against adverse cardiac remodelling and heart failure. *Cardiovasc Res.* **118**, 1771–1784 (2022). 34132787 10.1093/cvr/cvab206 PMC9215193

[479] Bozkurt, B. et al. Heart failure epidemiology and outcomes statistics: A report of the heart failure society of america. *J. Card. Fail* **29**, 1412–1451 (2023). 37797885 10.1016/j.cardfail.2023.07.006 PMC10864030

[480] Doiron, J. E. et al. Adjunctive therapy with an oral H(2)S donor provides additional therapeutic benefit beyond SGLT2 inhibition in cardiometabolic heart failure with preserved ejection fraction. *Br. J. Pharmacol.* **181**, 4294–4310 (2024). 10.1111/bph.16493 PMC12128175 38982742

[481] Datzmann, T. et al. Effects of sodium thiosulfate (Na(2)S(2)O(3)) during resuscitation from hemorrhagic shock in swine with preexisting atherosclerosis. *Pharm. Res* **151**, 104536 (2020). 10.1016/j.phrs.2019.104536 31734346

[482] Merz, T. et al. H(2)S in critical illness-a new horizon for sodium thiosulfate? *Biomolecules*. **12**, 543 (2022). 10.3390/biom12040543 PMC9029606 35454132

[483] de Koning, M. L. Y. et al. Safety and tolerability of sodium thiosulfate in patients with an acute coronary syndrome undergoing coronary angiography: a dose-escalation safety pilot study (SAFE-ACS). *J. Inter. Cardiol.* **2020**, 6014915 (2020). 10.1155/2020/6014915 PMC7532357 33041696

[484] Szczesny, B. et al. AP39, a novel mitochondria-targeted hydrogen sulfide donor, stimulates cellular bioenergetics, exerts cytoprotective effects and protects against the loss of mitochondrial DNA integrity in oxidatively stressed endothelial cells in vitro. *Nitric Oxide* **41**, 120–130 (2014). 24755204 10.1016/j.niox.2014.04.008 PMC4225488

[485] Abou-Hamdan, A. et al. Oxidation of H2S in mammalian cells and mitochondria. *Methods Enzymol.* **554**, 201–228 (2015). 25725524 10.1016/bs.mie.2014.11.042

[486] Magierowska, K. et al. The mitochondria-targeted sulfide delivery molecule attenuates drugs-induced gastropathy. Involvement of heme oxygenase pathway. *Redox Biol.* **66**, 102847 (2023). 37597422 10.1016/j.redox.2023.102847 PMC10458696

[487] Yan, X. et al. Endogenous H(2)S targets mitochondria to promote continual phagocytosis of erythrocytes by microglia after intracerebral hemorrhage. *Redox Biol.* **56**, 102442 (2022). 35998432 10.1016/j.redox.2022.102442 PMC9420393

[488] Fiorucci, S., Santucci, L. & Distrutti, E. NSAIDs, coxibs, CINOD and H2S-releasing NSAIDs: what lies beyond the horizon. *Dig. Liver Dis.* **39**, 1043–1051 (2007). 17997373 10.1016/j.dld.2007.09.001

[489] Vandiver, M. & Snyder, S. H. Hydrogen sulfide: a gasotransmitter of clinical relevance. *J. Mol. Med. (Berl.)* **90**, 255–263 (2012). 22314625 10.1007/s00109-012-0873-4 PMC3901014

[490] Costa, S. et al. Enhanced analgesic effects and gastrointestinal safety of a novel, hydrogen sulfide-releasing anti-inflammatory drug (ATB-352): a role for endogenous cannabinoids. *Antioxid. Redox Signal* **33**, 1003–1009 (2020). 32064887 10.1089/ars.2019.7884 PMC7578177

[491] Sasso, O. et al. Multitarget fatty acid amide hydrolase/cyclooxygenase blockade suppresses intestinal inflammation and protects against nonsteroidal anti-inflammatory drug-dependent gastrointestinal damage. *FASEB J.* **29**, 2616–2627 (2015). 25757568 10.1096/fj.15-270637 PMC4447230

[492] Wallace, J. L. et al. A proof-of-concept, phase 2 clinical trial of the gastrointestinal safety of a hydrogen sulfide-releasing anti-inflammatory drug. *Br. J. Pharm.* **177**, 769–777 (2020). 10.1111/bph.14641 PMC7024706 30834513

[493] Glanville, J. R. W. et al. Potent anti-inflammatory effects of an H(2) S-releasing naproxen (ATB-346) in a human model of inflammation. *FASEB J.* **35**, e21913 (2021). 34555204 10.1096/fj.201902918RR

[494] Schnitzer, T. J. et al. Comparison of the COX-inhibiting nitric oxide donator AZD3582 and rofecoxib in treating the signs and symptoms of Osteoarthritis of the knee. *Arthritis Rheum.* **53**, 827–837 (2005). 16342089 10.1002/art.21586

[495] Fiorucci, S. & Distrutti, E. COXIBs, CINODs and H(2)S-releasing NSAIDs: current perspectives in the development of safer non steroidal anti-inflammatory drugs. *Curr. Med. Chem.* **18**, 3494–3505 (2011). 21756233 10.2174/092986711796642508

[496] Fonseca, M. D., Cunha, F. Q., Kashfi, K. & Cunha, T. M. NOSH-aspirin (NBS-1120), a dual nitric oxide and hydrogen sulfide-releasing hybrid, reduces inflammatory pain. *Pharm. Res. Perspect.* **3**, e00133 (2015). 10.1002/prp2.133 PMC4492749 26236481

[497] Kodela, R., Chattopadhyay, M., Velazquez-Martinez, C. A. & Kashfi, K. NOSH-aspirin (NBS-1120), a novel nitric oxide- and hydrogen sulfide-releasing hybrid has enhanced chemo-preventive properties compared to aspirin, is gastrointestinal safe with all the classic therapeutic indications. *Biochem. Pharm.* **98**, 564–572 (2015). 26394025 10.1016/j.bcp.2015.09.014 PMC4656078

[498] Cenac, N. et al. A novel orally administered trimebutine compound (GIC-1001) is anti-nociceptive and features peripheral opioid agonistic activity and Hydrogen Sulphide-releasing capacity in mice. *Eur. J. Pain.* **20**, 723–730 (2016). 26541237 10.1002/ejp.798

[499] Paquette, J. M. et al. Safety, tolerability and pharmacokinetics of trimebutine 3-thiocarbamoylbenzenesulfonate (GIC-1001) in a randomized phase I integrated design study: single and multiple ascending doses and effect of food in healthy volunteers. *Clin. Ther.* **36**, 1650–1664 (2014). 25224876 10.1016/j.clinthera.2014.08.005

[500] Ullah, H. et al. The efficacy of S-Adenosyl methionine and probiotic supplementation on depression: a synergistic approach. *Nutrients*. **14**, 2751 (2022). 10.3390/nu14132751 PMC9268496 35807931

[501] Ullah, H. et al. Efficacy of a food supplement based on S-adenosyl methionine and probiotic strains in subjects with subthreshold depression and mild-to-moderate depression: a monocentric, randomized, cross-over, double-blind, placebo-controlled clinical trial. *Biomed. Pharmacother.* **156**, 113930 (2022). 36411659 10.1016/j.biopha.2022.113930

[502] Xu, F. et al. Integration of ATAC-Seq and RNA-Seq identifies key genes and pathways involved in the neuroprotection of S-adenosylmethionine against perioperative neurocognitive disorder. *Comput Struct. Biotechnol. J.* **21**, 1942–1954 (2023). 36942104 10.1016/j.csbj.2023.03.001 PMC10024148

[503] Bekdash, R. A. Methyl donors, epigenetic alterations, and brain health: understanding the connection. *Int. J. Mol. Sci*. **24**, 2346 (2023). 10.3390/ijms24032346 PMC9917111 36768667

[504] Pal, V. K. et al. Hydrogen sulfide blocks HIV rebound by maintaining mitochondrial bioenergetics and redox homeostasis. *Elife*. **10**, e68487 (2021). 10.7554/eLife.68487 PMC8660018 34792020

[505] Wang, X. B., Cui, H., Liu, X. & Du, J. B. Sulfur dioxide: foe or friend for life? *Histol. Histopathol.* **32**, 1231–1238 (2017). 28524210 10.14670/HH-11-904

